# Trifluoromethylcinnamanilide Michael Acceptors for Treatment of Resistant Bacterial Infections

**DOI:** 10.3390/ijms232315090

**Published:** 2022-12-01

**Authors:** Tomas Strharsky, Dominika Pindjakova, Jiri Kos, Lucia Vrablova, Pavel Smak, Hana Michnova, Tomas Gonec, Jan Hosek, Michal Oravec, Izabela Jendrzejewska, Alois Cizek, Josef Jampilek

**Affiliations:** 1 Department of Chemical Drugs, Faculty of Pharmacy, Masaryk University, Palackeho 1946/1, 612 00 Brno, Czech Republic; 2 Regional Centre of Advanced Technologies and Materials, Czech Advanced Technology and Research Institute, Palacky University, Slechtitelu 27, 783 71 Olomouc, Czech Republic; 3 Department of Analytical Chemistry, Faculty of Natural Sciences, Comenius University, Ilkovicova 6, 842 15 Bratislava, Slovakia; 4 Department of Biochemistry, Faculty of Medicine, Masaryk University, Kamenice 5, 625 00 Brno, Czech Republic; 5 Department of Infectious Diseases and Microbiology, Faculty of Veterinary Medicine, University of Veterinary Sciences Brno, Palackeho tr. 1946/1, 612 42 Brno, Czech Republic; 6 Department of Pharmacology and Toxicology, Veterinary Research Institute, Hudcova 296/70, 621 00 Brno, Czech Republic; 7 Global Change Research Institute CAS, Belidla 986/4a, 603 00 Brno, Czech Republic; 8 Institute of Chemistry, University of Silesia in Katowice, 40-007 Katowice, Poland; 9 Institute of Neuroimmunology, Slovak Academy of Sciences, Dubravska cesta 9, 845 10 Bratislava, Slovakia

**Keywords:** cinnamamides, Michael acceptors, antimicrobial activity, cytotoxicity, lipophilicity, structure–activity relationships, docking study

## Abstract

A series of thirty-two anilides of 3-(trifluoromethyl)cinnamic acid (series **1**) and 4-(trifluoromethyl)cinnamic acid (series **2**) was prepared by microwave-assisted synthesis. All the compounds were tested against reference strains *Staphylococcus aureus* ATCC 29213 and *Enterococcus faecalis* ATCC 29212 and resistant clinical isolates of methicillin-resistant *S. aureus* (MRSA) and vancomycin-resistant *E. faecalis* (VRE). All the compounds were evaluated in vitro against *Mycobacterium smegmatis* ATCC 700084 and *M. marinum* CAMP 5644. (2*E*)-3-[3-(Trifluoromethyl)phenyl]-*N*-[4-(trifluoromethyl)phenyl]prop-2-enamide (**1j**), (2*E*)-*N*-(3,5-dichlorophenyl)-3-[3-(trifluoromethyl)phenyl]prop-2-enamide (**1o**) and (2*E*)-*N*-[3-(trifluoromethyl)phenyl]-3-[4-(trifluoromethyl)-phenyl]prop-2-enamide (**2i**), (2*E*)-*N*-[3,5-bis(trifluoromethyl)phenyl]-3-[4-(trifluoromethyl)phenyl]-prop-2-enamide (**2p**) showed antistaphylococcal (MICs/MBCs 0.15–5.57 µM) as well as anti-enterococcal (MICs/MBCs 2.34–44.5 µM) activity. The growth of *M. marinum* was strongly inhibited by compounds **1j** and **2p** in a MIC range from 0.29 to 2.34 µM, while all the agents of series **1** showed activity against *M. smegnatis* (MICs ranged from 9.36 to 51.7 µM). The performed docking study demonstrated the ability of the compounds to bind to the active site of the mycobacterial enzyme InhA. The compounds had a significant effect on the inhibition of bacterial respiration, as demonstrated by the MTT assay. The compounds showed not only bacteriostatic activity but also bactericidal activity. Preliminary in vitro cytotoxicity screening was assessed using the human monocytic leukemia cell line THP-1 and, except for compound **2p**, all effective agents did show insignificant cytotoxic effect. Compound **2p** is an interesting anti-invasive agent with dual (cytotoxic and antibacterial) activity, while compounds **1j** and **1o** are the most interesting purely antibacterial compounds within the prepared molecules.

## 1. Introduction

Increasing microbial burden and the development of antimicrobial resistance (AMR) pose a major threat to human health worldwide. In 2019, it was estimated that there were almost 5 million deaths associated with bacterial AMR, including approx. 1.3 million deaths definitively caused by bacterial AMR. Lower respiratory tract infections accounted for more than 1.5 million resistance-related deaths in 2019, making it the most serious infectious syndrome. The six main pathogens for resistance-related deaths were *Staphylococcus aureus*, *Escherichia coli*, *Klebsiella pneumoniae*, *Streptococcus pneumoniae*, *Acinetobacter baumannii,* and *Pseudomonas aeruginosa*. Methicillin-resistant *S. aureus* caused more than 100,000 deaths attributed to AMR in 2019. Another high number of deaths were caused by resistant (not multidrug-resistant) *M. tuberculosis*, and third-generation cephalosporin-resistant *E. coli*, carbapenem-resistant *A. baumannii*, fluoroquinolone-resistant *E. coli*, carbapenem-resistant *K. pneumoniae*, and third-generation cephalosporin-resistant *K. pneumoniae* [1,2]. In addition to an increased risk of patient death, AMR represents a longer hospital stay and increased health care costs [1,2,3]. This state of affairs is extremely undesirable and, apart from various approaches [2,4], the most advantageous is of course the design of new entities with a new/innovative mechanism of action.

One design option of new drugs is inspiration from natural compounds with multiple activities [5,6,7,8]. One such interesting compound is cinnamic acid, which has a long history of human use as a component of plant fragrances and spices. Cinnamic acid and its derivatives occur naturally in larger quantities in plants. They are produced by a biochemical pathway providing phenylpropanoids, coumarins, lignins, isoflavonoids, flavonoids, stilbenes, aurones, anthocyanins, spermidines, and tannins and act as a plant hormone regulating cell growth and differentiation [9,10,11]. In the search for new pharmacologically active agents, cinnamic acid and its derivatives are promising substances with great potential for the development of new drugs. Cinnamic acid and its derivatives have attracted the attention of scientists in recent decades for their low toxicity and wide spectrum of biological activities, such as antibacterial/antimycobacterial, antiprotozoal, antiviral, anti-inflammatory, cytotoxic, antidiabetic, hepatoprotective, antioxidant, neuroprotective, and anxiolytic effects [12,13,14,15,16,17].

Our research team specializes in the design of multi-target anti-infective agents based on ring-substituted azanaphthalene bioisosteres; such hydroxynaphthanilides with activity against Gram-positive bacteria and mycobacteria [18,19,20]; and quinoline-like compounds (hydroxyquinolines, benzoxazoles, benzothiazoles) with antifungal, antibacterial, and antimycobacterial activities [21,22,23,24,25]. As part of the modifications to increase the solubility of mainly naphthalene derivatives, one ring was dismantled, resulting in the (*E*)-prop-1-en-1-ylbenzene scaffold. A number of cinnamic acid anilides were prepared and tested [26,27]. Subsequent chlorination of the benzene ring gave much more potent compounds [28], and as sufficient lipophilicity was found to be essential for the efficacy of these compounds, new series were prepared where the chlorine atoms were replaced by a trifluoromethyl group either in the *meta*- or *para*-position of the cinnamic acid benzene ring. Moieties with electron-withdrawing properties were also chosen as substituents on the anilide ring. Thus, 2 × 16 lipophilic compounds with electron-withdrawing substituents that cause electron deficiency throughout the conjugated double-bond system, including the amide group, were prepared. It can be stated that these designed agents meet the definition of so-called Michael acceptors, i.e., compounds in which generally double/triple bonds are conjugated with electron-withdrawing groups and which are able to react with nucleophiles, i.e., electron-rich substrates (e.g., NH or SH groups) [29], and thus have the ability to interact with many biological targets [12,13,30]. All the prepared compounds were tested against Gram-positive and mycobacterial strains and were also docked into the binding site of their potential intracellular target InhA, which has been previously reported to interact with similar compounds [31,32,33].

## 2. Results and Discussion

### 2.1. Synthesis and Physicochemical Properties

**Scheme 1:**
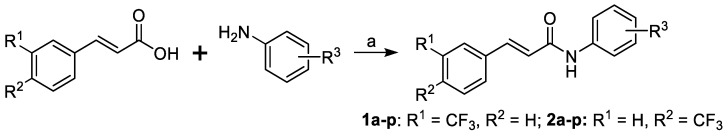
Synthesis of (2*E*)-*N*-phenyl-3-[3-(trifluoromethyl)phenyl]prop-2-enanilides **1a**–**p** and (2*E*)-*N*-phenyl-3-[4-(trifluoromethyl)phenyl]prop-2-enanilides **2a**–**p**. *Reagents and conditions:* (a) PCl_3_, chlorobenzene, MW, 45 min [28].

Anilides of 3-(trifluoromethyl)cinnamic acid (series **1**) and 4-(trifluoromethyl)cinnamic acid (series **2**) were synthesized according to Scheme 1 as described previously by Strharsky et al. [28]. Briefly, acid dissolved in dry chlorobenzene in the presence of phosphorus trichloride and the appropriate aniline in a microwave reactor yielded targeted *N*-arylcinnamamides (see Table 1).

**Figure 1:**
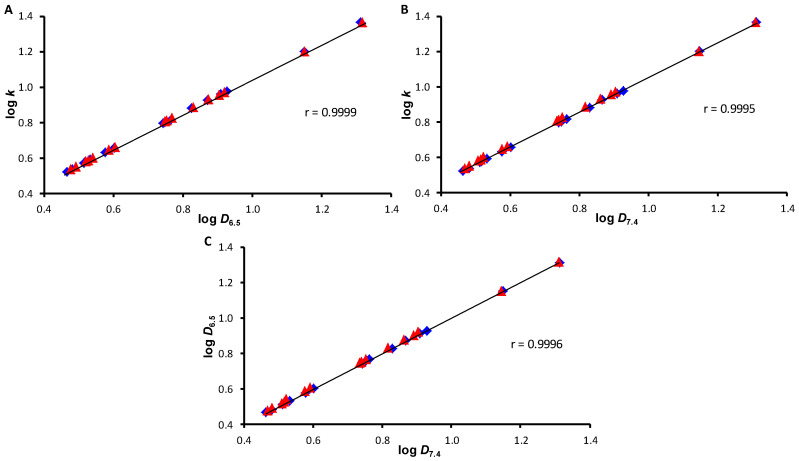
Mutual comparison of experimentally determined values of log *k*/log *D*_6.5_/log *D*_7.4_ (**A**–**C**) of prepared compounds **1a**–**2p**. (blue rhombuses = series **1**, red triangles = series **2**).

**Figure 2:**
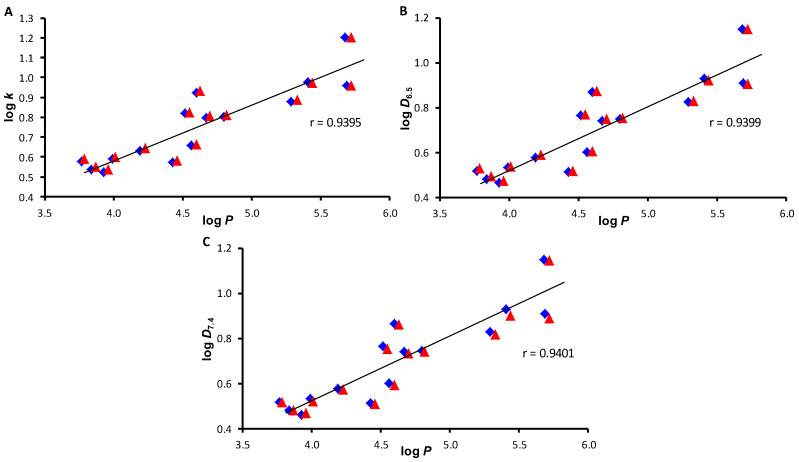
Correlation of experimental log *k*/log *D*_6.5_/log *D*_7.4_ (**A**–**C**) values with predicted log *P* (ACD/Percepta) values (**A**–**C**) of prepared compounds **1a**–**2p**. (blue rhombuses = series **1**, red triangles = series **2**).

Based on the previous results of our compounds, where lipophilicity was shown to significantly affect biological activities [26,27,28], lipophilicities were also experimentally determined for all the investigated compounds by RP-HPLC, as the logarithm of the capacity factor *k* and the logarithms of the distribution coefficients *D* at physiological pH values of 6.5 and 7.4 [34,35]. It is evident from the graphs in Figure 1 that the correlation coefficients of the dependences of log *k*/log *D*_6.5_/log *D*_7.4_ have a value of r = 0.999 (n = 32), which means that the investigated compounds behave insignificantly differently in aqueous media with different pH. Moreover, lipophilicity was calculated as log *P* and Clog *P* using two prediction programs. Unfortunately, ChemBioDrawUltra does not distinguish individual positional isomers, so *ortho*-, *meta*-, and *para*-isomers have the same values of lipophilicity, which are also the same for compounds from series **1** and **2**. Therefore, only log *P* values predicted by ACD/Percepta can be used for correlations with experimental values. These matches are shown in the graphs in Figure 2, and according to the correlation coefficient r~0.940 (n = 32), it can be argued that even the predicted lipophilicity values correlate with the experimentally measured ones. However, the largest variations were observed (no difference for series **1**, **2**) for compounds substituted with 2-Cl, 2-CF_3_, 2,5-Cl, 4-F, 4-CF_3_, and 3,5-F, 3,5-Cl. These are mainly substituents capable of forming hydrogen and other types of non-bonding interactions both with the surrounding environment and within the molecule. Overall, series **1** (3-CF_3_) is less lipophilic than series **2** (4-CF_3_), but the differences in the experimental data are insignificant. Unsubstituted derivatives **1a**/**2a** are the least lipophilic compounds, while derivatives **1o**/**2o** (3,5-Cl) and **1p**/**2p** (3,5-CF_3_) are the most lipophilic compounds. Any anilide substitution in the *ortho*-position significantly decreases lipophilicity compared to *para*- or *meta*-substitution.

In addition to lipophilicity, Table 1 also shows the predicted (ACD/Percepta) electronic σ parameters of the whole substituted anilide ring characterizing the ability to withdraw or donate electrons to molecule system.

### 2.2. In Vitro Antimicrobial Activity

All the investigated compounds were in vitro tested against Gram-positive bacteria and mycobacteria. First, universally sensitive collection strains of *Staphylococcus aureus* ATCC 29213 and *Enterococcus faecalis* ATCC 29212 were selected. The second aspect of strain selection was the current state of occurrence of strains with an epidemiologically significant type of resistance, represented by clinical isolates of human and veterinary origin, i.e., different sequence types limited to human and animal populations, e.g., methicillin-resistant *Staphylococcus aureus* (MRSA) SA 3202, SA 630, 63718 isolates carrying the *mecA* gene [36]. In the case of vancomycin-resistant *E. faecalis* (VRE) 342B, 368, and 725B isolates carrying the *vanA* gene [37], these were isolates from wild birds that were colonized from US hospital wastewater. In addition, all the compounds were in vitro tested against fast-growing *Mycobacterium smegmatis* ATCC 700084 [38] and slow-growing *Mycobacterium marinum* CAMP 5644 [39] as a safe alternative to *Mycobacterium tuberculosis*. Activities are expressed as the minimum inhibitory concentrations (MICs) and the minimum bactericidal concentrations (MBCs); see Table 2. To establish that a compound demonstrates a bactericidal effect against a particular tested strain, it must meet the condition MIC/MBC ≤ 4 [36,40]. MBC values that meet this requirement, i.e., the compound is bactericidal, are indicated in bold in Table 2.

Table 2 shows that out of 32 tested compounds, nine compounds showed activity against the tested microorganisms: seven from series **1** (R^1^ = 3-CF_3_) and two from series **2** (R^2^ = 4-CF_3_). All other compounds were inactive, i.e., their MICs were ≥256 µg/mL. The effective compounds are substituted in the *meta*-positions F, Cl, CF_3_, only **1g** and **1j** are substituted at C_(4)_ of the cinnamic scaffold. Compounds **1j** (R^3^ = 4-CF_3_), **1o** (R^3^ = 3,5-Cl) and **2i** (R^3^ = 3-CF3), **2p** (R^3^ = 3,5-CF_3_) demonstrated activity in the entire spectrum of tested microorganisms. Compounds **1c** (R^3^ = 3-F), **1f** (R^3^ = 3-Cl), **1g** (R^3^ = 4-Cl), **1i** (R^3^ = 3-CF_3_), and **1p** (R^3^ = 3,5-CF_3_) had only antistaphylococcal activity. The activity of the compounds significantly exceeded the MIC values of ampicillin against staphylococcal strains and even for **1o** and **2p** the activity was comparable to that of ampicillin against enterococcal strains. It should be noted that compounds showed comparable antistaphylococcal activities both against methicillin-susceptible *S. aureus* and MRSA isolates, therefore it can be assumed that the presence of the *mecA* gene (which encodes an alternative transpeptidase and causes methicillin resistance [41]) [36] in MRSA does not affect the activity of these compounds. Thus, it can be similarly speculated concerning the specific activity against *Staphylococcus* sp. that the close activity of the compounds against both *E. faecalis* and VRE indicates a mechanism of action unrelated to vancomycin resistance [37]. It is important to mention that all compounds demonstrated bactericidal activity; see bolded MBC values in Table 2.

The growth of *M. marinum* was inhibited only by four compounds **1j** (R^3^ = 4-CF_3_), **1o** (R^3^ = 3,5-Cl), **1p** (R^3^ = 3,5-CF_3_), **2i** (R^3^ = 3-CF_3_), and **2p** (R^3^ = 3,5-CF_3_) in a wide MIC range from 0.29 to 22.2 µM, but all compounds were more effective than standard isoniazid. On the other hand, only **2i** (R^3^ = 3-CF_3_) had no effect on fast-growing *M. smegnatis*; the other derivatives had MICs from 1.17 to 51.7 µM; again, they were more effective than isoniazid.

Since the compounds act more on aerobic staphylococci than on facultatively anaerobic enterococci [42], known among other things for high resistance to disinfection procedures and antibiotics [43,44,45], it was hypothesized that a possible mechanism of action could be inhibition of respiration, and therefore a standard MTT test was performed with the most active derivatives. The MTT assay can be used to assess cell growth by measuring respiration. Respiratory activity of bacterial cells (which is finally reflected in their viability) less than 70% after exposure to the MIC values for each tested compound is considered as a positive result of this assay. This low level of cell oxidative metabolism indicates inhibition of cell growth by inhibition of respiration [46,47]. The lowest multiples of MIC values that achieved more than 70% inhibition of *S. aureus* ATCC 29213 viability (%) are shown in Table 3. It can be noted that compounds **1o** (R^3^ = 3,5-Cl) and **1j** (R^3^ = 4-CF_3_) showed a decrease in viability of <70% at half and even a quarter of their MIC value and compounds **1f** (R^3^ = 3-Cl), **1i**/**2i** (R^3^ = 3-CF_3_), and **2p** (R^3^ = 3,5-CF_3_) showed a decrease in viability of <70% at their MICs, which suggests that these agents may act through inhibition of the respiratory chain. Compounds **1c** and **2p** inhibited respiratory chain by ca. 98% at a value of 2× MIC, so they are able to significantly affect it, compared to, e.g., ciprofloxacin or ampicillin.

### 2.3. In Vitro Cell Viability

Preliminary in vitro cytotoxicity screening of the antimicrobially effective compounds (Table 2) was performed using human monocytic leukemia cell line THP-1 in the culture medium containing 10% FBS, and it was expressed as IC_50_ values (see Table 2). The investigated anilides can be divided into three groups. Compounds **1p** and **2p** (R^3^ = 3,5-CF_3_) with IC_50_ of 6.7 and 8.9 µM, respectively, had the most pronounced activity on THP-1 cells. Insignificant cytotoxic effect (IC_50_ > 10 µM) was found for **1f** (R^3^ = 3-Cl), **1g** (R^3^ = 4-Cl), **1i**/**2i** (R^3^ = 3-CF_3_) and any cytotoxic effect (IC_50_ > 30 µM) was found for **1j** (R^3^ = 4-CF_3_) and **1o** (R^3^ = 3,5-Cl). Higher concentrations (than 10 or 30 µM) were not possible to evaluate due to precipitation of the tested compounds in water medium. Based on all these observations, it can be concluded that the high antimicrobial activity of 3,5-CF_3_-substituted compounds is associated with significant cytotoxic effect against eukaryotic cells. On the other hand, the other still effective compounds, especially agents **1j** and **1o**, have an insignificant toxic effect, which makes them interesting for further study.

### 2.4. Structure–Activity Relationships

**Figure 3:**
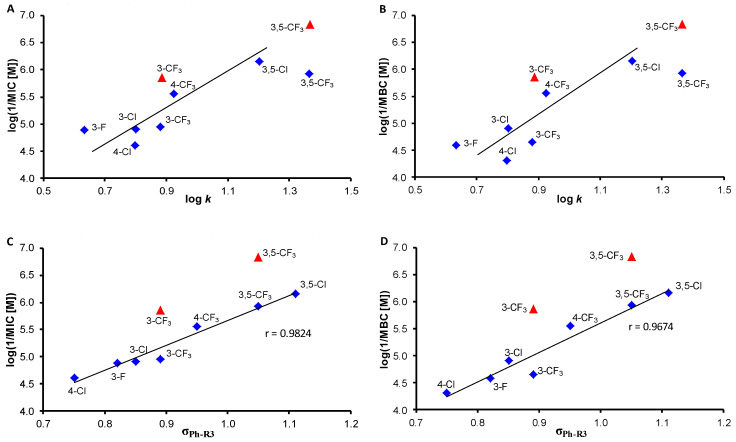
Dependences of in vitro bacteriostatic log(1/MIC [M]) (**A**,**C**) and bactericidal log(1/MBC [M]) (**B**,**D**) activity against *S. aureus* ATCC 29213 on lipophilicity expressed as log *k* and electronic σ parameters of whole substituted anilide of studied compounds (blue rhombuses = series **1**, red triangles = series **2**).

Since only nine (7 + 2) compounds expressed antimicrobial activity, extensive discussions of structure–activity relationships (SAR) are not possible, but certain trends can be found. The dependence of the activity against *S. aureus*, expressed as log(1/MIC [M]) or log(1/MBC [M], on lipophilicity (log *k*) is illustrated in the graphs in Figure 3A,B. It is possible to see a bilinear trend where the activity increases with increasing value of log *k* up to 1.2 and then decreases. The activity is also significantly affected by the electron-withdrawing properties of the anilide substituents, as shown in Figure 3C,D. This linear dependence has a correlation coefficient of r = 0.9824 (bacteriostatic activity) and r = 0.9674 (bactericidal activity) for the seven derivatives of series **1**. The dependences of activities on lipophilicity or electronic σ parameters for MRSA isolates are similar and therefore not shown.

**Figure 4:**
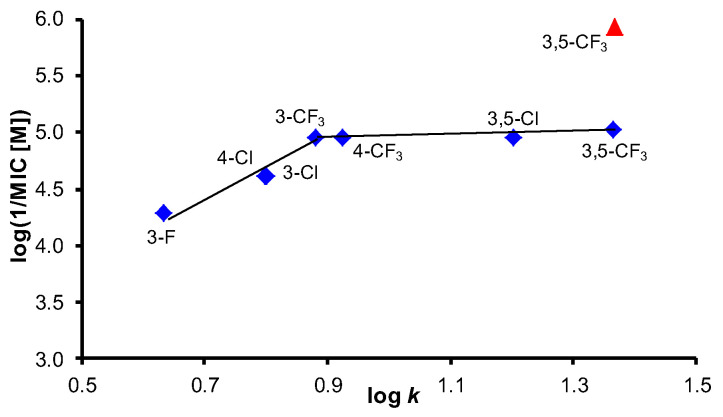
Dependences of in vitro antimycobacterial activity expressed as log(1/MIC [M]) against *M. smegamtis* on lipophilicity expressed as log *k* of studied compounds (blue rhombuses = series **1**, red triangles = series **2**).

Only two compounds from each series were effective against *E. faecalis*/VRE. Therefore, it can only be stated that compounds with significant lipophilicity and electron-withdrawing properties seem to be preferred for a stronger effect. The SAR discussion of activities against *M. marinum* is similarly brief. Substitution with CF_3_ groups on C_(3,5)_’ or C_(4)_’, lipophilicity (log *k*) in the interval from 0.9 to 1.3 and σ ca. 1 seems to be advantageous. The SAR discussion related to *M. smegmatis* seems to be more interesting. Only compound **2i** is not active, thus series **1** can be discussed. As can be seen from Figure 4, the dependence of activity expressed as log(1/MIC [M]) on lipophilicity (log *k*) increases with increasing value of log *k* value to approx. 0.9 and then is constant. The course of the dependence of activity on electronic σ parameters is the same as the dependence on lipophilicity: it increases with increasing σ value up to 0.9 and then is linear.

As mentioned above, this is a follow-up study dealing with the antimicrobial activity of ring-substituted cinnamanilides. In total, anilides of cinnamic acid [26,27], anilides of 4-chlorocinnamic acid/3,4-dichlorocinnamic acid [28], and the anilides of 3-(trifluoromethyl)cinnamic acid/4-(trifluoromethyl)cinnamic acid discussed here were prepared. The elimination of one of the aromatic rings from the dinuclear aromatic skeleton and the formation of the (*E*)-prop-1-en-1-ylbenzene structure improved the overall solubility and enabled, in addition to the modification of the anilide part, the substitution of the aromatic ring of cinnamic acid. The substitution of the phenyl core of cinnamic acid with lipophilic and electron-withdrawing substituents was inspired by our previous studies dealing with salicylamide derivatives [36,48,49,50]. Among these variously substituted cinnamic acid derivatives, several interesting compounds in terms of bioactivity were found.

**Figure 5:**
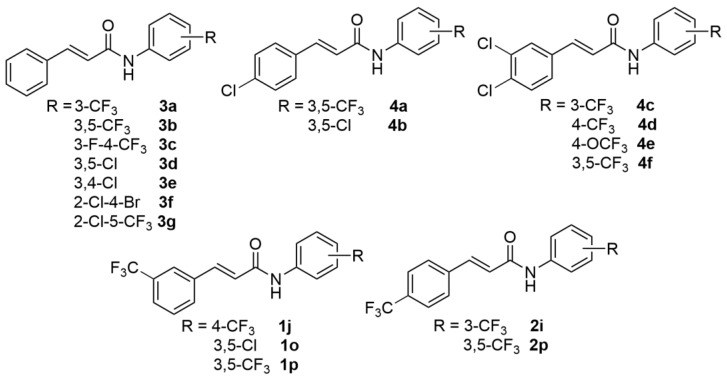
Structures of investigated ring-substituted cinnamanilides with significant antimicrobial (**3a**–**e**, **4a**–**f**, **1j**,**o**,**p**, **2i**,**p**) and antiplasmodial (**3f**) activity or anti-inflammatory (**3g**) potential [26,27,28,58,59].

Within the primary series with substitutions on the anilide ring only, compounds **3a**–**c** (Figure 5) were identified as active against *S. aureus*/MRSA (average MIC ~24 µM). The compounds were completely inactive against *E. faecalis*/VRE. The MTT assay showed 86% inhibition of *S. aureus* respiration by compound **3c** at its MIC value. Compounds **3a** and **3b** showed bactericidal effect in concentrations close to MICs and ability to inhibit the growth of staphylococcal biofilm and disrupted mature biofilm in concentrations close to MICs. In addition, these compounds were evaluated for their ability to potentiate clinically used antibiotics. Vancomycin, tetracycline, and ciprofloxacin were chosen, and it was found that compound **3b** showed a synergistic effect with tetracycline and ciprofloxacin and additivity with vancomycin against MRSA. These three antibiotics have different mechanisms of action, to which bacteria develop different mechanisms of resistance, so it can be expected that our compound works by its own, different mechanism of action [26,51]. Based on these results, it can be assumed that our compounds are able to act as inhibitor of the *S. aureus* NorA efflux pump, as was described for ferulic acid derivatives [52,53]. Removal of fluoroquinolones and tetracyclines from cells by efflux pumps represents one of the main mechanisms of resistance to them [3]. The additive effect with vancomycin may be associated with damage to the bacterial membrane [54] including possible inhibition of peptidoglycan synthesis by affecting the biosynthesis of the peptidoglycan precursor uridine-diphosphate-*N*-acetylglucosamine [55]. Compounds **3a**, **3b**, **3d**, and **3e** were active against *M. tuberculosis* H37Ra (average MIC ~24 µM). MTT assay showed 77% inhibition of *M. tuberculosis* respiration by compounds **3b** and **3d** at their MIC values [26]. Of the chlorinated compounds representing the next generation of cinnamanilide derivatives, 3,4-dichlorinated derivatives proved to be more effective. Compounds **4a**–**f** (both 4-Cl and 3,4-Cl derivatives) were active against *S. aureus*/MRSA (average MIC ~0.7 µM). The MTT assay demonstrated 95% inhibition of *S. aureus* respiration by these compounds at their MICs. The exception was **4f**, which inhibited the respiration of *S. aureus* by 96% at 0.25× MIC. Only derivatives **4c**–**f** (3,4-Cl series) were effective against *E. faecalis*/VRE (average MIC ~18 µM). In addition, only **4c**–**f** were active against *M. smegmatis* and *M. marinum* (average MIC ~4.1 µM) safe models mimicking *M. tuberculosis*. All the effective compounds showed bactericidal activity [28,56]. Of the compounds discussed in this paper, the majority of active derivatives were from series **1** (R^1^ = 3-CF_3_), although two of the effective derivatives from series **2** were more potent. Active against all tested microorganisms were compounds **1j**, **1o**, **2i**, and **2p** (average MIC ~1.4 µM against *S. aureus*/MRSA and ~15 µM against *E. faecalis*/VRE). The MTT assay demonstrated 98% inhibition of *S. aureus* respiration by compounds **1j**, **2i**, and **2p** at their MICs. The exception was **1o**, which inhibited the respiration of *S. aureus* by 98% at 0.25× MIC. All active compounds showed bactericidal activity. Inhibition of cellular respiration by inhibiting membrane-bound ATPases resulting in a subsequent bactericidal effect of cinnamaldehyde was studied, e.g., by Gill and Holley [57]. In addition, compounds **1j**, **1p**, and **2p** were active against *M. smegmatis* and *M. marinum* (average MIC ~4.1 µM).

Based on these observations, it can be concluded that the substitution of the anilide with Cl or CF_3_ in the *meta*-position (preferably in the C_(3,5)_′ positions) or in the *para*-position is essential for antibacterial/antimycobacterial activity. The simultaneous substitution of the phenyl ring of cinnamic acid with 3,4-Cl, 3-CF_3_, or 4-CF_3_ increases the effect and broadens the spectrum of activity. Overall, however, these are electron-deficient (σ range 0.9–1.2) and lipophilic compounds (log *k* range 1.2–1.4/log *P* range 5.5–6.5), which penetrate well through the membranes of pathogens and have the ability to bind to electron-rich targets.

However, in the recently published study describing the antiplasmodial activity of the basic series of cinnamanilides (type 3 compounds), the C_(3)_′/C_(3,5)_′ substituted derivatives showed only moderate activity and the compounds having at least one substituent in the C_(2)_′ anilide position were the most active [58], e.g., compound **3f** with IC_50_ = 0.58 µM against *Plasmodium falciparum* 3D7. Similar findings regarding the need for substitution at the C_(2)_′ anilide position were found for NF-κB inhibition, when compound **3g** showed the anti-inflammatory potential comparable to that prednisone [59]. However, even in these cases, the physicochemical characteristics are similar (a lipophilic electron-deficient molecule with a conjugated amide bond), only the position of the substituents for optimal activity differs, which may be due to the different target site and specific steric conditions of the binding pocket. Therefore, it can be stated that all these active compounds can be considered Michael acceptors [29,60,61,62].

Due to their structure (overall electronic parameters, electron-withdrawing substituents, and conjugated –C(=O)–N(H)– bond), ring-substituted cinnamanilides are able to interact with a variety of biological targets in many signaling pathways in cells, as reported recently [12,13,30,63,64,65], and thus meet the requirement of being therapeutically useful multi-target agents. The theory of the dependence of activity on the ability of compounds to act as Michael acceptors would be clearly confirmed by the preparation and biological evaluation of hydrogenated analogues of active cinnamanilides. There is also an additional question about activity modification if the configuration of the derivatives were not *trans* (*E*-isomers) but *cis* (*Z*-isomers), since the physiological activity of *cis*-cinnamic acid was higher than that of *trans*-cinnamic acid in many respects [66,67,68,69].

### 2.5. Docking Study

The compounds exhibiting antimicrobial activity against tested microorganisms (Table 3) were docked into the binding site of their potential target—enzyme InhA. The protein has been shown to interact with ligands that have similar size and structural features as our compounds [31,32,33,70]. In the study by Sabbah et al. [15], the binding mode and structural features of triclosan were employed in the fragment-based design of InhA inhibitors, of which the activity was also experimentally evaluated. Enzyme InhA, part of the fatty acid synthase II (FAS II) complex, is an NADH-dependent enzyme involved in the reduction of long-chain fatty acids, which are essential components of the unique cell envelope of mycobacteria [15]. Recently, the protein has become an interesting target for the development of antituberculotic drugs [15,31].

**Figure 6:**
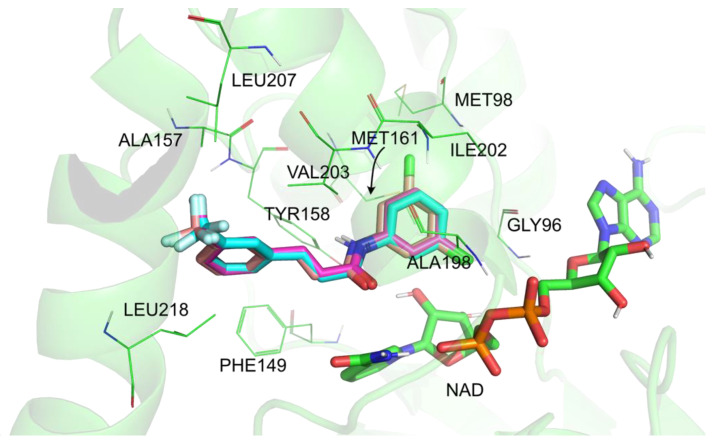
Compounds **1c** (cyan), **1f** (magenta), and **1o** (orange) docked into binding site of InhA with NAD (green).

**Figure 7:**
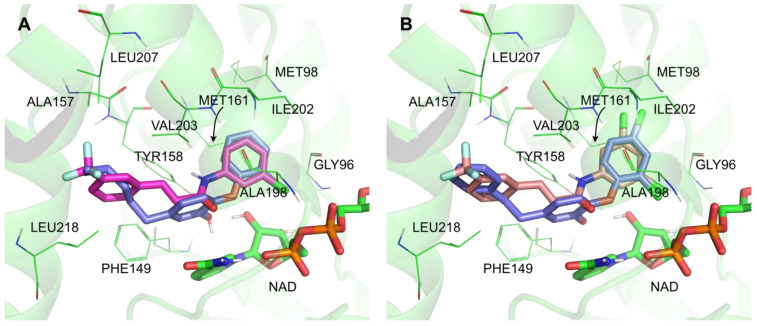
Superposition of compound **1f** (magenta) docked into the binding site of InhA with NAD (green) and ligand from X-ray structure 4OYR (violet) (**A**). Superposition of compound **1o** (orange) and X-ray structure 3FNH (violet) on the right (**B**). Structural alignment of the proteins was performed.

Software AutoDock Vina was employed for the docking; structure 4OYR was used for InhA. The source organism for the protein is *M. tuberculosis*. The suitability of the used docking procedure was verified on a set of InhA ligands with available experimental data and X-ray structures [31]. The details are provided in Supplementary Materials (Tables S1 and S2 and Figures S1 and S2). Figure 6 shows the binding mode of compounds **1c**, **1f**, and **1o** in the binding site of InhA. They show π-interactions with TYR158 and PHE149, and hydrophobic interactions with LEU218, VAL203, and ALA198. Comparisons of these complexes with X-ray structures of diaryl ether derivatives from Chollet and others [31,32,33] or triclosan X-ray structure [70] reveal that all these compounds have very similar binding mechanisms (Figure 7, Table S3 and Figure S3). This supports the theory that our compounds could interact with InhA.

**Figure 8:**
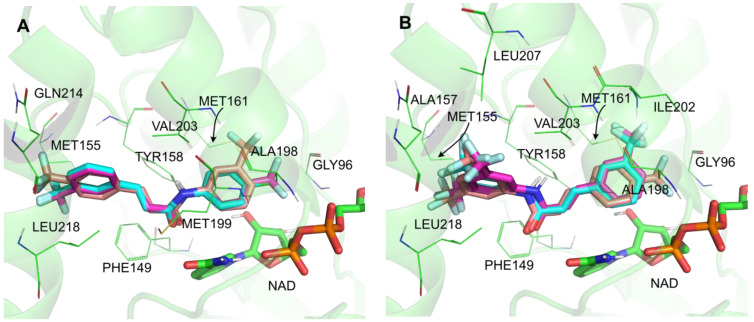
Compounds **1g** (cyan), **1j** (magenta), and **2i** (orange) docked into binding site of InhA with NAD (green) (**A**). Compounds **1i** (cyan), **1p** (magenta), and **2p** (orange) (**B**).

Figure 8A shows the binding mode of compounds **1g**, **1j**, and **2i** in the binding site of InhA, and Figure 8B shows the binding mode of compounds **1i**, **1p**, and **2p**. The binding modes for these six compounds are slightly different from those observed with compounds **1c**, **1f**, and **1o**. However, the compounds interact with similar residues.

The binding mode of our compounds in InhA is consistent with the published X-ray structures; therefore, InhA is a possible target of our compounds.

A different potential target of our compounds is subunit c of ATP synthase. It is the transmembrane portion of the protein (F_0_ rotor). Compounds binding to this protein have shown antituberculotic activity [71] but also activity against *Streptococcus* spp. [72]. Such an activity was also observed with our compounds. There are, however, only limited experimental and structural data available for this protein. Moreover, it is not surrounded by water since it is a transmembrane domain. Therefore, it is not suitable for docking (see Supplementary Materials and Figure S4 for details).

## 3. Materials and Methods

### 3.1. General Methods

All reagents were purchased from Merck (Sigma-Aldrich, St. Louis, MO, USA) and Alfa (Alfa-Aesar, Ward Hill, MA, USA). Reactions were performed using an Anton-Paar Monowave 50 microwave reactor (Graz, Austria). The melting points were determined on a Kofler hot-plate apparatus HMK (Franz Kustner Nacht KG, Dresden, Germany) and are uncorrected. All ^1^H- and ^13^C-NMR spectra were recorded on a JEOL ECZR 400 MHz NMR spectrometer (400 MHz for ^1^H and 100 MHz for ^13^C, Jeol, Tokyo, Japan) in dimethyl sulfoxide-*d_6_* (DMSO-*d_6_*). ^1^H and ^13^C chemical shifts (δ) are reported in ppm. High-resolution mass spectra were measured using a high-performance liquid chromatograph Dionex UltiMate^®^ 3000 (Thermo Scientific, West Palm Beach, FL, USA) coupled with an LTQ Orbitrap XL^TM^ Hybrid Ion Trap-Orbitrap Fourier Transform Mass Spectrometer (Thermo Scientific) equipped with a HESI II (heated electrospray ionization) source in the positive and negative mode.

### 3.2. Synthesis

#### General Procedure for Synthesis of Carboxamide Derivatives **1a**–**2q**

The 3-trifluoromethylcinnamic acid/4-trifluoromethylcinnamic acid (1.0 mM) was suspended in dry chlorobenzene (6.0 mL) at ambient temperature and phosphorus trichloride (0.5 mM, 0.5 eq.), and the corresponding substituted aniline (1.0 mM, 1 eq.) were added dropwise. The reaction mixture was transferred to the microwave reactor, where the synthesis was performed (30 min, 130 °C). Then the mixture was cooled to 40 °C, and then the solvent was removed to dryness under reduced pressure. The residue was washed with hydrochloride acid and water. The crude product was recrystallized from ethanol.

*(2E)-N-Phenyl-3-[3-(trifluoromethyl)phenyl]prop-2-enamide* (**1a**) [73]. Yield 68%; Mp 141–143 °C; ^1^H-NMR (DMSO-*d_6_*), δ: 10.25 (s 1H), 7.98 (s, 1H), 7.94 (d, J = 7.8 Hz, 1H), 7.94 (d, J = 7.8 Hz, 1H), 7.72–7.66 (m, 4H), 7.36–7.32 (m, 2H), 7.10–7.06 (m, 1H), 6.97 (d, J = 16.0 Hz, 1H); ^13^C-NMR (DMSO-*d_6_*), δ: 163.1, 139.1, 138.3, 135.9, 131.5, 130.2, 129.8, (q, J = 31.8 Hz), 128.8, 126.0, (q, J = 3.9 Hz), 124.0 (q, J = 272.6 Hz), 124.0 (q, J = 3.9 Hz), 119.2 (Figure S5); HR-MS: for C_16_H_11_ONF_3_, [M-H]^−^ calculated 290.0798 *m/z*, found 290.0802 *m/z*.

*(2E)-N-(2-Fluorophenyl)-3-[3-(trifluoromethyl)phenyl]prop-2-enamide* (**1b**). Yield 71%; Mp 115–117 °C; ^1^H-NMR (DMSO-*d_6_*), δ: 9.97 (s, 1H), 8.16 (td, J = 7.9, 2.1 Hz, 1H), 7.98 (s, 1H), 7.92 (d, J = 7.8 Hz, 1H), 7.76–7.65 (m, 3H), 7.30–7.12 (m, 4H); ^13^C-NMR (DMSO-*d_6_*), δ: 163.5, 153.2 (d, J = 244.7 Hz), 138.8, 135.9, 131.6, 130.1, 129.8 (q, J = 31.8 Hz), 126.4 (d, J = 11.6 Hz), 126.0 (q, J = 3.9 Hz), 125.0 (d, J = 7.7 Hz), 124.4 (d, J = 3.9 Hz), 124.0 (q, J = 272.6 Hz), 124.1, 123.9 (q, J = 3.9 Hz), 123.3, 115.4 (d, J = 19.3 Hz) (Figure S6); HR-MS: for C_16_H_10_ONF_4_ [M-H]^−^ calculated 308.0704 *m/z*, found 308.0708 *m/z*.

*(2E)-N-(3-Fluorophenyl)-3-[3-(trifluoromethyl)phenyl]prop-2-enamide* (**1c**) [74]. Yield 69%; Mp 113–115 °C; ^1^H-NMR (DMSO-*d_6_*), δ: 10.45 (s, 1H), 7.97 (s, 1H), 7.92 (d, J = 7.6 Hz, 1H), 7.74–7.65 (m, 4H), 7.38–7.34 (m, 2H), 6.94 (d, J = 15.8 Hz, 1H), 6.90–6.87 (m, 1H); ^13^C-NMR (DMSO-*d_6_*), δ: 163.4, 162.2 (d, J = 240.8 Hz), 140.9 (d, J = 10.6 Hz), 138.9, 135.8, 131.6, 130.5 (d, J = 8.7 Hz), 130.2, 129.8 (q, J = 31.8 Hz), 126.2 (q, J = 3.9 Hz), 124.0, 124.1 (q, J = 3.9 Hz), 124.0 (q, J = 271.7 Hz), 115.0 (d, J = 2.9 Hz), 110.0 (d, J = 21.2 Hz), 106.1 (d, J = 27.0 Hz) (Figure S7); HR-MS: for C_16_H_10_ONF_4_ [M-H]^−^ calculated 308.0704 *m/z*, found 308.0701 *m/z*.

*(2E)-N-(4-Fluorophenyl)-3-[3-(trifluoromethyl)phenyl]prop-2-enamide* (**1d**). Yield 75%; Mp 149–151 °C; ^1^H-NMR (DMSO-*d_6_*), δ: 10.32 (s, 1H), 7.98 (s, 1H), 7.94 (d, J= 7.6 Hz, 1H), 7.76 (d, J = 8.2 Hz, 1H), 7.73–7.67 (m, 4H), 7.20–7.17 (m, 2H), 6.93 (d, J = 15.8 Hz, 1H); ^13^C-NMR (DMSO-*d_6_*), δ: 163.0, 158.1 (d, J = 241.3 Hz), 138.4, 135.9, 135.5 (d, J = 2.9 Hz), 131.5, 130.2, 129.8 (q, J = 31.8 Hz), 126.0 (q, J = 2.9 Hz), 124.3, 124.0 (q, J = 4.3 Hz), 124.0 (q, J = 273.1 Hz), 120.9 (d, J = 7.2 Hz), 115.5 (d, J = 21.7 Hz) (Figure S8); HR-MS: for C_16_H_10_ONF_4_ [M-H]^−^ calculated 308.0704 *m/z*, found 308.0706 *m/z*.

*(2E)-N-(2-Chlorophenyl)-3-[3-(trifluoromethyl)phenyl]prop-2-enamide* (**1e**). Yield 72%; Mp 118–120 °C; ^1^H-NMR (DMSO-*d_6_*), δ: 9.68 (s, 1H), 8.02 (s, 1H), 7.99 (dd, J = 8.2, 1.4 Hz, 1H), 7.95 (d, J = 7.8 Hz, 1H), 7.77 (d, J = 7.8 Hz, 1H), 7.73–7.67 (m, 2H), 7.52 (dd, J = 7.8, 1.4 Hz, 1H), 7.36 (td, J = 7.8, 1.8 Hz, 1H), 7.28 (d, J = 15.6 Hz, 1H), 7.20 (td, J = 7.8, 1.8 Hz, 1H); ^13^C-NMR (DMSO-*d_6_*), δ: 163.5, 139.1, 135.9, 134.9, 131.8, 130.1, 129.8 (q, J = 31.8 Hz), 129.5, 127.5, 126.1 (q, J = 3.9 Hz), 126.1, 125.5, 125.2, 124.0, 124.0 (q, J = 271.7 Hz), 124.0 (q, J = 3.9 Hz) (Figure S9); HR-MS: for C_16_H_10_ONClF_3_, [M-H]^−^ calculated 324.0409 *m/z*, found 324.0412 *m/z*.

*(2E)-N-(3-Chlorophenyl)-3-[3-(trifluoromethyl)phenyl]prop-2-enamide* (**1f**). Yield 68%; Mp 126–128 °C; ^1^H-NMR (DMSO-*d_6_*), δ: 10.44 (s, 1H), 7.99–7.94 (m, 3H), 7.77 (d, J = 7.8 Hz, 1H), 7.72–7.67 (m, 2H), 7.53 (d, J = 8.7 Hz, 1H), 7.37 (t, J = 8.2 Hz, 1H), 7.14 (dd, J = 8.0, 1.1 Hz, 1H), 6.93 (d, J = 15.6 Hz, 1H); ^13^C-NMR (DMSO-*d_6_*), δ: 163.4, 140.6, 139.0, 135.8, 133.2, 131.6, 130.5, 130.2, 129.8 (q, J = 31.8 Hz), 126.1 (q, J = 3.9 Hz), 124.1 (q, J = 3.9 Hz), 124.0, 124.0 (q, J = 271.7 Hz), 123.21, 118.7, 117.6 (Figure S10); HR-MS: for C_16_H_10_ONClF_3_, [M-H]^−^ calculated 324.0409 *m/z*, found 324.0414 *m/z*.

*(2E)-N-(4-Chlorophenyl)-3-[3-(trifluoromethyl)phenyl]prop-2-enamide* (**1g**). Yield 62%; Mp 162–164 °C; ^1^H-NMR (DMSO-*d_6_*), δ: 10.39 (s, 1H), 7.99 (s, 1H), 7.94 (d, J = 7.8 Hz, 1H), 7.77–7.67 (m, 5H), 7.42–7.38 (m, 2H), 6.94 (d, J = 16.0 Hz, 1H); ^13^C-NMR (DMSO-*d_6_*), δ: 163.2, 138.7, 138.1, 135.8, 131.5, 130.2, 129.8 (q, J = 31.8 Hz), 128.8, 127.1, 126.1 (q, J = 3.9 Hz), 124.2, 124.0 (q, J = 272.6 Hz), 124.0 (q, J = 3.9 Hz), 120.7 (Figure S11); HR-MS: for C_16_H_10_ONClF_3_, [M-H]^−^ calculated 324.0409 *m/z*, found 324.0406 *m/z*.

*(2E)-N-[2-(Trifluoromethyl)phenyl]-3-[3-(trifluoromethyl)phenyl]prop-2-enamide* (**1h**). Yield 78%; Mp 111–113 °C; ^1^H-NMR (DMSO-*d_6_*), δ: 9.74 (s, 1H); 8.01 (s, 1H); 7.95 (d, J = 7.8 Hz, 1H); 7.77 (d, J = 7.8 Hz, 2H), 7.73–7.65 (m, 4H), 7.47 (t, J = 7.8 Hz, 1H); 7.16 (d, J = 16.0 Hz, 1H); ^13^C-NMR (DMSO-*d_6_*), δ: 164.2, 139.1, 135.8, 135.2, 133.0, 131.7, 130.1, 129.8 (q, J = 31.8 Hz), 129.5, 126.6, 126.3 (q, J = 4.8 Hz), 126.1 (q, J = 3.9 Hz), 124.1 (q, J = 29.9 Hz), 124.1 (q, J = 3.9 Hz), 124.0 (q, J = 271.7 Hz), 123.6 (q, J = 273.6 Hz), 123.6 (Figure S12); HR-MS: for C_17_H_10_ONF_6_, [M-H]^−^ calculated 358.0672 *m/z*, found 358.0668 *m/z*.

*(2E)-N,3-bis[3-(Trifluoromethyl)phenyl]prop-2-enamide* (**1i**) [75]. Yield 64%; Mp 136–138 °C; ^1^H-NMR (DMSO-*d_6_*), δ: 10.60 (s, 1H), 8.22 (s, 1H), 8.00 (s, 1H), 7.95 (d, J = 7.8 Hz, 1H), 7.86 (d, J = 8.7 Hz, 1H), 7.78–7.67 (m, 3H), 7.59 (t, J = 8.0 Hz, 1H), 7.44–7.42 (m, 1H), 6.94 (d, J = 15.6 Hz, 1H); ^13^C-NMR (DMSO-*d_6_*), δ: 163.6, 139.9, 139.1, 135.7, 131.6, 130.2, 130.1, 129.8 (q, J = 31.8 Hz), 129.5 (q, J = 31.8 Hz), 126.2 (q, J = 3.9 Hz), 124.2 (q, J = 3.9 Hz), 124.1 (q, J = 272.6 Hz), 124.0 (q, J = 272.6 Hz), 123.9, 122.8, 119.8 (q, J = 3.9 Hz), 115.2 (q, J = 3.9 Hz) (Figure S13); HR-MS: for C_17_H_10_ONF_6_, [M-H]^−^ calculated 358.0672 *m/z*, found 358.0669 *m/z*.

*(2E)-3-[3-(Trifluoromethyl)phenyl]-N-[4-(trifluoromethyl)phenyl]prop-2-enamide* (**1j**). Yield 72%; Mp 118–120 °C; ^1^H-NMR (DMSO-*d_6_*), δ: 10.61 (s, 1H), 8.00 (s, 1H), 7.95 (d, J = 7.8 Hz, 1H), 7.91 (d, J = 8.7 Hz, 2H), 7.76–7.67 (m, 5H), 6.97 (d, J = 16Hz, 1H); ^13^C-NMR (DMSO-*d_6_*), δ: 163.6, 142.7, 139.2, 135.7, 131.6, 130.2, 129.8 (q, J = 31.8 Hz), 126.2 (multiplet), 124.4 (q, J = 271.7 Hz), 124.1 (q, J = 3.9 Hz), 124.0 (q, J = 272.6 Hz), 124.0, 123.5 (q, J = 31.8 Hz), 119.1; (Figure S14); HR-MS: for C_17_H_10_ONF_6_, [M-H]^−^ calculated 358.0672 *m/z*, found 358.06738 *m/z*.

*(2E)-N-(2,4-Difluorophenyl)-3-[3-(trifluoromethyl)phenyl]prop-2-enamide* (**1k**). Yield 63%; Mp 143–145 °C; ^1^H-NMR (DMSO-*d_6_*), δ: 9.99 (s, 1H), 8.08 (td, J = 8.9, 6.4 Hz, 1H), 7.99 (s, 1H), 7.93 (d, J = 7.8 Hz, 1H), 7.76 (d, J = 7.8 Hz, 1H), 7.71–7.67 (m, 2H), 7.38–7.32 (m, 1H), 7.17 d, J = 15.6 Hz, 1H), 7.13–7.07 (m, 1H); ^13^C-NMR (DMSO-*d_6_*), δ: 163.5, 158.3 (dd, J = 243.7, 11.6 Hz), 153.5 (dd, J = 248.5, 12.5 Hz), 138.9, 135.9, 131.6, 130.2, 129.8 (q, J = 31.8 Hz), 126.1 (q, J = 3.9 Hz), 124.8 (dd, J = 9.6, 2.9 Hz), 124.0 (q, J = 272.6 Hz), 124.0 (q, J = 3.9 Hz), 123.8, 122.9 (dd, J = 11.6, 3.9 Hz), 111.2 (dd, J = 21.2, 3.9 Hz), 104.2 (dd, J = 27.0, 24.1 Hz) (Figure S15); HR-MS: for C_16_H_9_ONF_5_ [M-H]^−^ calculated 326.0610 *m/z*, found 326.0606 *m/z*.

*(2E)-N-(3,5-Difluorophenyl)-3-[3-(trifluoromethyl)phenyl]prop-2-enamide* (**1l**). Yield 61%; Mp 131–133°C; ^1^H-NMR (DMSO-*d_6_*), δ: 10.62 (s, 1H), 8.00 (s, 1H), 7.95 (d, J = 7.8 Hz, 1H), 7.77 (d, J = 7.8 Hz, 1H), 7.74–7.67 (m, 2H), 7.42–7.40 (m, 2H), 6.96–6.87 (m, 2H); ^13^C-NMR (DMSO-*d_6_*), δ: 163.6, 162.5 (dd, J = 242.8, 15.4 Hz), 141.6 (t, J = 13.5 Hz), 139.5, 135.6, 131.6, 130.2, 129.8 (q, J = 31.8 Hz), 126.3 (q, J = 3.9 Hz), 124.2 (q, J = 3.9 Hz), 124.0 (q, J = 272.6 Hz), 123.6, 102.3–102.0 (multiplet), 98.7 (t, J = 26.0 Hz) (Figure S16); HR-MS: for C_16_H_9_ONF_5_ [M-H]^−^ calculated 326.0610 *m/z*, found 326.0605 *m/z*.

*(2E)-N-(2,4-Dichlorophenyl)-3-[3-(trifluoromethyl)phenyl]prop-2-enamide* (**1m**). Yield 68%; Mp 134–136 °C; ^1^H-NMR (DMSO-*d_6_*), δ: 9.75 (s, 1H), 8.03 (d, J = 8.7 Hz, 1H), 8.01 (s, 1H), 7.95 (d, J = 7.8 Hz, 1H), 7.77 (d, J = 8.2 Hz, 1H), 7.73–7.67 (m, 3H), 7.45 (dd, J = 8.9, 2.5 Hz, 1H), 7.27 (d, J= J = 15.6 Hz, 1H); ^13^C-NMR (DMSO-*d_6_*), δ: 163.6, 139.4, 135.8, 134.1, 131.8, 130.2, 129.8 (q, J = 31.8 Hz), 129.1, 128.9, 127.6, 126.2, 126.2 (q, J = 3.9 Hz), 126.0, 124.0 (q, J = 272.6 Hz), 124.0 (q, 3.9 Hz), 123.7 (Figure S17); HR-MS: for C_16_H_9_ONCl_2_F_3_ [M-H]^−^ calculated 358.0019 *m/z*, found 358.0026 *m/z*.

*(2E)-N-(2,5-Dichlorophenyl)-3-[3-(trifluoromethyl)phenyl]prop-2-enamide* (**1n**). Yield 72%; Mp 145–147 °C; ^1^H-NMR (DMSO-*d_6_*), δ: 9.77 (s, 1H), 8.18 (d, J = 2.7 Hz, 1H), 8.02 (s, 1H), 7.95 (d, J = 7.8 Hz, 1H), 7.78 (d, J = 7.8 Hz, 1H), 7.72 (d, J = 16.0 Hz, 1H), 7.69 (t, J = 7.8 Hz, 1H), 7.56 (d, J = 8.7 Hz, 1H), 7.32 (d, J = 16.0 Hz, 1H), 7.27 (dd, J = 8.7, 2,7 Hz, 1H); ^13^C-NMR (DMSO-*d_6_*), δ: 163.8, 139.6, 136.1, 135.8, 131.9, 131.6, 130.9, 130.2, 129.8 (q, J = 31.8 Hz), 126.2 (q, J = 3.9 Hz), 125.5, 124.0 (q, J = 3.9 Hz), 124.0 (q, J = 272.6 Hz), 123.8, 123.7, 123.4 (Figure S18); HR-MS: for C_16_H_9_ONCl_2_F_3_ [M-H]^−^ calculated 358.0019 *m/z*, found 358.0021 *m/z*.

*(2E)-N-(3,5-Dichlorophenyl)-3-[3-(trifluoromethyl)phenyl]prop-2-enamide* (**1o**). Yield 65%; Mp 124–126 °C; ^1^H-NMR (DMSO-*d_6_*), δ: 10.59 (s, 1H), 8.00 (s, 1H), 7.96–7.94 (m, 1H), 7.79–7.75 (m, 3H), 7.74 (d, J = 15.6 Hz, 1H), 7.70 (t, J = 7.8 Hz, 1H), 7.30 (m, 1H), 7.24 (d, J = 15.6 Hz, 1H); ^13^C-NMR (DMSO-*d_6_*), δ: 163.7, 141.4, 139.6, 135.6, 134.2, 131.6, 130.2, 129.8 (q, J = 31.8 Hz), 126.3 (q, J = 3.9 Hz), 124.3 (q, J = 3.9 Hz), 124.0 (q, J = 271.7 Hz), 123.6, 122.7, 117.3 (Figure S19); HR-MS: for C_16_H_9_ONCl_2_F_3_ [M-H]^−^ calculated 358.0019 *m/z*, found 358.0012 *m/z*.

*(2E)-N-[3,5-bis(Trifluoromethyl)phenyl]-3-[3-(trifluoromethyl)phenyl]prop-2-enamide* (**1p**). Yield 70%; Mp 147–149 °C; ^1^H-NMR (DMSO-*d_6_*), δ: 10.90 (s, 1H), 8.34 (s, 2H), 7.99 (s, 1H), 7.95 (d, J = 7.8 Hz, 1H), 7.78–7.73 (m, 3H), 7.69 (t, J = 7.8 Hz, 1H), 6.89 (d, J = 16 Hz, 1H); ^13^C-NMR (DMSO-*d_6_*), δ: 164.0, 141.0, 139.9, 135.5, 131.6, 130.8 (q, J = 32.8 Hz), 130.2, 129.8 (q, J = 31.8 Hz), 126.4 (q, J = 3.9 Hz), 124.4 (q, J = 3.9 Hz), 124.0 (q, J = 272.6 Hz), 123.3, 123.2 (q, J = 272.6 Hz), 118.9 (q, J = 3.9 Hz), 116.2 (multiplet) (Figure S20); HR-MS: for C_18_H_9_ONF_9_, [M-H]^−^ calculated 426.0546 *m/z*, found 426.0552 *m/z*.

*(2E)-N-Phenyl-3-[3-(trifluoromethyl)phenyl]prop-2-enamide* (**2a**) [76]. Yield 69%; Mp 154–157 °C; ^1^H-NMR (DMSO-*d_6_*), δ: 10.30 (s, 1H), 7.85–7.79 (m, 4H), 7.71 (d, J = 7.8 Hz, 2H), 7.66 (d, J = 15.6 Hz, 1H), 7.35 (t, J = 8.0 Hz, 2H), 7.08 (t, J = 7.5 Hz, 1H), 6.97 (d, J = 15.6 Hz, 1H); ^13^C-NMR (DMSO-*d_6_*), δ: 163.0, 139.1, 138.8, 138.4, 129.4 (q, J = 31.8 Hz), 128.9, 128.4, 125.9 (q, J = 3.9 Hz), 125.1, 124.1 (q, J = 272.6 Hz), 123.6, 119.3 (Figure S21); HR-MS: for C_16_H_11_ONF_3_, [M-H]^−^ calculated 290.0798 *m/z*, found 290.0791 *m/z*.

*(2E)-N-(2-Fluorophenyl)-3-[4-(trifluoromethyl)phenyl]prop-2-enamide* (**2b**) [74]. Yield 68%; Mp 164–166 °C; ^1^H-NMR (DMSO-*d_6_*), δ: 10.03 (s, 1H), 8.11 (td, J = 7.8, 2.3 Hz, 1H), 7.85–7.80 (m, 4H), 7.68 (d, J = 15.6 Hz, 1H), 7.31–7.26 (m, 1H), 7.23–7.14 (m, 3H); ^13^C-NMR (DMSO-*d_6_*), δ: 163.4, 153.3 (d, J = 244.7 Hz), 138.9, 138.8, 129.5 (q, J = 32.8 Hz), 128.4, 126.2 (d, J = 11.6 Hz), 125.9 (q, J = 3.9 Hz), 125.2 (d, J = 6.7 Hz), 124.7, 124.4 (d, J = 3.9 Hz), 124.1 (q, J = 272.6 Hz), 123.6, 115.5 (d, J = 19.3 Hz) (Figure S22); HR-MS: for C_16_H_10_ONF_4_ [M-H]^−^ calculated 308.0704 *m/z*, found 308.0690 *m/z*.

*(2E)-N-(3-Fluorophenyl)-3-[4-(trifluoromethyl)phenyl]prop-2-enamide* (**2c**) [74]. Yield 66%; Mp 146–148 °C; ^1^H-NMR (DMSO-*d_6_*), δ: 10.53 (s, 1H), 7.86–7.80 (m, 4H), 7.74–7.72 (m, 1H), 7.68 (d, J = 15.6 Hz, 1H), 7.40–7.36 (m, 2H), 6.94 (d, J = 15.6 Hz, 1H), 6.93–6.90 (m, 1H); ^13^C-NMR (DMSO-*d_6_*), δ: 163.3, 162.1 (d, J = 241.6 Hz), 140.8 (d, J = 10.4 Hz), 139.0, 138.6, 130.5 (d, J = 9.2 Hz), 129.5 (q, J = 31.2 Hz), 128.4, 125.9 (q, J = 3.5 Hz), 124.7, 124.1 (q, J = 272.8 Hz), 115.1(d, J = 2.3 Hz), 110.0 (d, J = 20.8 Hz), 106.1 (d, J = 26.6 Hz) (Figure S23); HR-MS: for C_16_H_10_ONF_4_ [M-H]^−^ calculated 308.0704 *m/z*, found 308.0708 *m/z*.

*(2E)-N-(4-Fluorophenyl)-3-[4-(trifluoromethyl)phenyl]prop-2-enamide* (**2d**) [74]. Yield 72%; Mp 149–151 °C; ^1^H-NMR (DMSO-*d_6_*), δ: 10.35 (s, 1H), 7.85–7.79 (m, 4H), 7.75–7.71 (m, 2H), 7.66 (d, 15.6 Hz, 1H), 7.21–7.15 (m, 2H), 6.93 (d, J = 15.6 Hz, 1H); ^13^C-NMR (DMSO-*d_6_*), δ: 162.9, 158.2 (d, J = 239.9 Hz), 138.7 (d, J = 1.9 Hz), 138.4, 135.5 (d, J = 1.9 Hz), 129.4 (q, J = 31.8 Hz), 128.3, 125.8 (q, J = 3.9 Hz), 124.9, 124.0 (q, J = 271.7 Hz), 121.0 (d, J = 7.7 Hz), 115.4 (d, J = 22.2 Hz) (Figure S24); HR-MS: for C_16_H_10_ONF_4_ [M-H]^−^ calculated 308.0704 *m/z*, found 308.0701 *m/z*.

*(2E)-N-(2-Chlorophenyl)-3-[4-(trifluoromethyl)phenyl]prop-2-enamide* (**2e**). Yield 71%; Mp 171–173 °C; ^1^H-NMR (DMSO-*d_6_*), δ: 9.77 (s, 1H), 7.94 (dd, J = 8.1, 1.1 Hz, 1H), 7.87–7.80 (m, 4H), 7.69 (d, J = 15.6 Hz, 1H), 7.53 (dd, J = 8.2, 1.4 Hz, 1H), 7.37 (td, J = 7.8, 1.4 Hz, 1H), 7.25 (d, J = 15.6 Hz, 1H), 7.21 (td, J = 7.8, 1.4 Hz, 1H); ^13^C-NMR (DMSO-*d_6_*), δ: 163.5, 139.0, 138.8, 134.8, 129.5, 129.5 (q, J = 31.8 Hz), 128.4, 127.5, 126.2, 125.8 (q, J = 3.9 Hz), 125.5, 124.6, 124.0 (q, J = 272.6 Hz) (Figure S25); HR-MS: for C_16_H_10_ONClF_3_, [M-H]^−^ calculated 324.0409 *m/z*, found 324.0403 *m/z*.

*(2E)-N-(3-Chlorophenyl)-3-[4-(trifluoromethyl)phenyl]prop-2-enamide* (**2f**). Yield 64%; Mp 150–152 °C; ^1^H-NMR (DMSO-*d_6_*), δ: 10.49 (s, 1H), 7.94 (t, J = 2.1 Hz, 1H), 7.86–7.79 (m, 4H), 7.68 (d, J = 15.6 Hz, 1H), 7.55–7.52 (m, 1H), 7.37 (t, J = 8.0 Hz, 1H), 7.14 (ddd, J = 8.0, 2.1, 0.9 Hz, 1H), 6.93 (d, J = 15.6 Hz, 1H); ^13^C-NMR (DMSO-*d_6_*), δ: 163.3, 140.5, 139.0, 138.6, 133.2, 130.5, 129.6 (q, J = 31.8 Hz), 128.4, 125.9 (q, J = 3.9 Hz), 124.6, 124.1 (q, J = 272. 6 Hz), 123.3, 118.7, 117.7 (Figure S26); HR-MS: for C_16_H_10_ONClF_3_ [M-H]^−^ calculated 324.0409 *m/z*, found 324.0412 *m/z*.

*(2E)-N-(4-Chlorophenyl)-3-[4-(trifluoromethyl)phenyl]prop-2-enamide* (**2g**). Yield 61%; Mp 185–187 °C; ^1^H-NMR (DMSO-*d_6_*), δ: 10.44 (s, 1H), 7.86–7.79 (m, 4H), 7.73 (d, J = 9.1 Hz, 2H), 7.67 (d, J = 15.6 Hz, 1H), 7.40 (d, J = 8.7 Hz, 2H), 6.94 (d, J = 15.6 Hz, 1H); ^13^C-NMR (DMSO-*d_6_*), δ: 163.1, 138.7, 138.02, 129.5 (q, J = 31.8 Hz), 128.8, 128.4, 127.11, 125.9 (q, J = 3.9 Hz), 124.8, 124.1 (q, J = 271.7 Hz), 120.8 (Figure S27); HR-MS: for C_16_H_10_ONClF_3_ [M-H]^−^ calculated 324.0409 *m/z*, found 324.0409 *m/z*.

*(2E)-N-[2-(Trifluoromethyl)phenyl]-3-[4-(trifluoromethyl)phenyl]prop-2-enamide* (**2h**). Yield 67%; Mp 189–191 °C; ^1^H-NMR (DMSO-*d_6_*), δ: 9.84 (s, 1H), 7.86 (d, J = 8.7 Hz, 2H), 7.81 (d, J = 8.7 Hz, 2H), 7.77 (d, J = 8.2 Hz, 1H), 7.72 (t, J = 7.8 Hz, 1H), 7.67 (d, J = 15.6 Hz, 1H), 7.66–7.63 (m, 1H), 7.48 (t, J = 7.8 Hz, 1H), 7.14 (d, J = 15.6 Hz, 1H); ^13^C-NMR (DMSO-*d_6_*), δ: 164.2, 139.1, 138.7, 135.2, 133.0, 129.7, 129.5 (q, J = 31.8 Hz), 128.4, 126.7, 126.3 (q, J = 5.8 Hz), 125.9 (q, J = 3.9 Hz), 124.4 (q, J = 28.9 Hz), 124.2, 124.1 (q, J = 271.7 Hz), 123.6 (q, J = 272.6 Hz) (Figure S28); HR-MS: for C_17_H_10_ONF_6_, [M-H]^−^ calculated 358.0672 *m/z*, found 358.0662, *m/z*.

*(2E)-N-[3-(Trifluoromethyl)phenyl]-3-[4-(trifluoromethyl)phenyl]prop-2-enamide* (**2i**). Yield 69%; Mp 134–136 °C; ^1^H-NMR (DMSO-*d_6_*), δ: 10.64 (s, 1H), 8.21 (s, 1H), 7.88–7.84 (m, 3H), 7.79 (d, J = 7.9 Hz, 2H), 7.70 (d, J = 15.8 Hz, 1H), 7.58 (t, J = 8.1 Hz, 1H), 7.42 (d, J = 7.6 Hz, 1H), 6.94 (d, J = 15.8 Hz, 1H); ^13^C-NMR (DMSO-*d_6_*), δ: 163.5, 139.8, 139.1, 138.5, 130.1, 129.6 (q, J = 32.4 Hz), 129.6 (q, J = 32.4 Hz), 128.4, 125.9 (q, J = 3.5 Hz), 124.5, 124.1 (q, J = 272.8 Hz), 124.0 (q, J = 271.7 Hz), 122.8, 119.8 (q, J = 3.5 Hz), 115.3 (q, J = 3.5 Hz) (Figure S29); HR-MS: for C_17_H_10_ONF_6_ [M-H]^−^ calculated 358.0672 *m/z*, found 358.0667 *m/z*.

*(2E)-N,3-bis[4-(Trifluoromethyl)phenyl]prop-2-enamide* (**2j**). Yield 72%; Mp 192–194 °C; ^1^H-NMR (DMSO-*d_6_*), δ: 10.66 (s, 1H), 7.91 (d, J = 8.2 Hz, 2H), 7.86 (d, J = 8.2 Hz. 2H), 7.81 (d, J = 8.7 Hz, 2H), 7.71 (d, J = 16.0 Hz, 1H), 7.71 (d, J = 8.7 Hz, 2H), 6.97 (d, J = 16.0 Hz, 1H); ^13^C-NMR (DMSO-*d_6_*), δ: 163.6, 142.6, 139.3, 138.6, 129.6 (q, J = 31.8 Hz), 128.5, 126.2 (q, J = 3.9 Hz), 125.9 (q, J= 3.9 Hz), 124.6, 124.4 (q, J= 270.7 Hz), 124.1 (q, J= 272.6 Hz), 123.5 (q, J= 31.8 Hz), 119.2 (Figure S30); HR-MS: for C_17_H_10_ONF_6_ [M-H]^−^ calculated 358.0672 *m/z*, found 358.0667 *m/z*.

*(2E)-N-(2,4-Difluorophenyl)-3-[4-(trifluoromethyl)phenyl]prop-2-enamide* (**2k**). Yield 66%; Mp 141–143 °C; ^1^H-NMR (DMSO-*d_6_*), δ: 10.07 (s, 1H), 8.08–8.02 (m, 1H), 7.85–7.80 (4H), 7.67 (d, J = 15.6 Hz, 1H), 7.36 (ddd, J = 11.3, 8.8, 2.7 Hz, 1H), 7.16 (d, J = 15.6 Hz, 1H), 7.14–7.08 (m, 1H); ^13^C-NMR (DMSO-*d_6_*), δ: 163.5, 158.4 (dd, J = 243.7, 11.6 Hz), 153.7 (dd, J = 247.6, 12.5 Hz), 139.0, 138.7, 129.5 (q, J = 31.8 Hz), 128.4, 125.9 (q, J = 3.9 Hz), 125.0 (multiplet), 124.4, 124.1 (q, J = 272.6 Hz), 122.8 (dd, J = 11.6, 3.9 Hz), 111.2 (dd, J = 22.2, 2.9 Hz), 104.2 (dd, J = 27.0, 24.1 Hz) (Figure S31); HR-MS: for C_16_H_9_ONF_5_ [M-H]^−^ calculated 326.0610 *m/z*, found 326.0611 *m/z*.

*(2E)-N-(3,5-Difluorophenyl)-3-[4-(trifluoromethyl)phenyl]prop-2-enamide* (**2l**). Yield 63%; Mp 188–190 °C; ^1^H-NMR (DMSO-*d_6_*), δ: 10.67 (s, 1H), 7.85 (d, J = 8.2 Hz, 2H), 7.80 (d, J = 8.2 Hz, 2H), 7.70 (d, J = 15.6 Hz, 1H), 7.44–7.38 (m, 2H), 6.93 (tt, J = 9.6, 2.3 Hz, 1H), 6.89 (d, J = 15.6 Hz, 1H); ^13^C-NMR (DMSO-*d_6_*), δ: 163.6, 162.5 (dd, J = 242.8, 15.4 Hz), 141.5 (t, J = 13.5 Hz), 139.5, 138.5, 129.7 (q, J = 31.8 Hz), 128.5, 125.9 (q, J = 3.9 Hz), 124.2, 124.0 (q, J = 271.7 Hz), 102.3–102.0 (multiplet), 98.7 (t, J = 27.0 Hz) (Figure S32); HR-MS: for C_16_H_9_ONF_5_, [M-H]^−^ calculated 326.0610 *m/z*, found 326.0606 *m/z*.

*(2E)-N-(2,4-Dichlorophenyl)-3-[4-(trifluoromethyl)phenyl]prop-2-enamide* (**2m**). Yield 68%; Mp 188–190 °C; ^1^H-NMR (DMSO-*d_6_*), δ: 9.84 (s, 1H), 7.99 (d, J = 9.1 Hz, 1H), 7.87–7.80 (m, 4H), 7.69 (d, J = 15.6 Hz, 1H), 7.69 (d, J = 2.7 Hz, 1H), 7.45 (dd, J = 8.7, 2.3 Hz, 1H), 7.25 (d, J = 15.6 Hz, 1H); ^13^C-NMR (DMSO-*d_6_*), δ: 163.6, 139.4, 138.7, 134.0, 129.6 (q, J = 31.8 Hz), 129.2, 129.0, 128.5, 127.6, 126.5, 126.4, 125.9 (q, J = 3.9 Hz), 124.4, 124,1 (q, J = 271.7 Hz) (Figure S33); HR-MS: for C_16_H_9_ONCl_2_F_3_ [M-H]^−^ calculated 358.0019 *m/z*, found 358.0017 *m/z*.

*(2E)-N-(2,5-Dichlorophenyl)-3-[4-(trifluoromethyl)phenyl]prop-2-enamide* (**2n**). Yield 75%; Mp 180–182 °C; ^1^H-NMR (DMSO-*d_6_*), δ: 9.86 (s, 1H), 8.14 (d, J = 2.7 Hz, 1H), 7.86 (d, J = 8.7 Hz, 2H), 7.82 (d, J = 8.7 Hz, 2H), 7.70 (d, J = 15.6 Hz, 1H), 7.57 (d, J = 8.7 Hz, 1H), 7.30 (d, J = 15.6 Hz, 1H), 7.27 (dd, J = 8.7, 2.7 Hz, 1H); ^13^C-NMR (DMSO-*d_6_*), δ: 163.7, 139.6, 138.6, 136.1, 131.6, 130.9, 129.6 (q, J = 31.8 Hz), 128.5, 125.9 (q, J = 3.9 Hz), 125.6, 124.3, 124.2, 124.1 (q, J = 272.6 Hz), 123.7 (Figure S34); HR-MS: for C_16_H_9_ONCl_2_F_3_, [M-H]^−^ calculated 358.0019 *m/z*, found 358.0012 *m/z*.

*(2E)-N-(3,5-Dichlorophenyl)-3-[4-(trifluoromethyl)phenyl]prop-2-enamide* (**2o**). Yield 73%; Mp 177–179 °C; ^1^H-NMR (DMSO-*d_6_*), δ: 10.63 (s, 1H), 7.85 (d, J = 8.2 Hz, 2H), 7.80 (d, J = 8.2 Hz, 2H), 7.75 (d, J = 1.8 Hz, 2H), 7.70 (d, J = 16.0 Hz, 1H), 7.29 (t, J = 2.1 Hz, 1H), 6.87 (d, J = 16.0 Hz, 1H); ^13^C-NMR (DMSO-*d_6_*), δ: 163.6, 141.4, 139.6, 138.4, 134.2, 129.7 (q, J = 31.8 Hz), 128.5, 125.9 (q, J = 3.9 Hz), 124.0 (q, J = 272.6 Hz), 124.2, 122.7, 117.4 (Figure S35); HR-MS: for C_16_H_9_ONCl_2_F_3_ [M-H]^−^ calculated 358.0019 *m/z*, found 358.0018 *m/z*.

*(2E)-N-[3,5-bis(Trifluoromethyl)phenyl]-3-[4-(trifluoromethyl)phenyl]prop-2-enamide* (**2p**). Yield 65%; Mp 207–209 °C; ^1^H-NMR (DMSO-*d_6_*), δ: 10.95 (s, 1H), 8.34 (s, 2H), 7.87–7.85 (m, 2H), 7.81–7.77 (m, 3H), 7.73 (d, J = 15.6 Hz, 1H), 6.89 (d, J = 15.6 Hz, 1H); ^13^C-NMR (DMSO-*d_6_*), δ: 163.9, 140.9, 139.9, 138.3, 130.8 (q, J = 32.8 Hz), 129.8 (q, J = 31.8 Hz), 128.6, 125.9 (q, J = 3.9 Hz), 124.0 (q, J = 271.7 Hz), 123.9, 123.2 (q, J = 273.6 Hz), 118.9 (q, J = 3.9 Hz), 116.3 (multiplet) (Figure S36); HR-MS: for C_18_H_9_ONF_9_, [M-H]^−^ calculated 426.0546 *m/z*, found 426.0534 *m/z*.

### 3.3. Lipophilicity Determination by HPLC

Experimental determination of lipophilicity values (log *k*, log *D*_6.5_, and log *D*_7.4_) was performed under the same conditions and on the same device as described by Strharsky et al. [28]. The log *k* values of individual compounds are shown in Table 1.

### 3.4. In Vitro Antibacterial Evaluation

In vitro antibacterial activity of the synthesized compounds was investigated against reference strains *Staphylococcus aureus* ATCC 29213 and *Enterococcus faecalis* ATCC 29212 and representatives of multidrug-resistant clinical isolates of methicillin-resistant *S. aureus* [36,41] and vancomycin-resistant *E. faecalis* [37]. The minimum inhibitory concentrations (MICs) were evaluated by the microtitration broth method according to the CLSI [77,78] as described recently [50]. The results are summarized in Table 2.

### 3.5. Determination of Minimum Bactericidal Concentrations

For the above-mentioned strains/isolates, the agar aliquot subculture method [79,80] was used as a test for bactericidal agents, as described recently [50].

### 3.6. MTT Assay

The percent inhibition of respiration of *S. aureus* ATCC 29213 was determined through the MTT assay. The assay was performed as described recently [50]. The percent viability is calculated through the comparison of a measured value and that of the uninhibited control: % viability = OD_570E_/OD_570P_ × 100, where OD_570E_ is the reading from the compound-exposed cells, while OD_570P_ is the reading from the uninhibited cells (positive control). Cytotoxic potential is determined by a percent viability of <70% [46,47]. The results are summarized in Table 3.

### 3.7. In Vitro Antimycobacterial Evaluation

The evaluation of in vitro antimycobacterial activity of the compounds was performed against *Mycobacterium marinum* CAMP 5644 and *M. smegmatis* ATCC 700084 as previously described [28]. The MIC value is routinely and widely used in bacterial assays and is a standard detection limit according to the CLSI [78]. The results are summarized in Table 2.

### 3.8. In Vitro Cell Viability Analysis

Human monocytic leukemia THP-1 cells obtained from the European Collection of Cell Cultures (ECACC, Salisbury, UK) were used for in vitro antiproliferative assays. A recently described procedure [81] was used. The results are shown in Table 2.

### 3.9. Docking Study

Receptors preparation: The X-ray structure of InhA (4OYR) [31] was obtained from RCSB PDB. The structures were checked, and water residues and other small molecules were removed with the use of PyMOL 2.5.4 [82]. For InhA, nicotinamide adenine dinucleotide (NAD) was left in the structure as a part of the receptor since it is present in X-ray structures of InhA-ligand complexes [31]. Leap (AmberTools in version 22 [83]) was employed to fill missing atoms in the structures. Since HIS residues are not in the binding site of any of the used structures, the protonation state of HIS residues was not adjusted. The input files for docking were prepared with the use of ADFR suite 1.0 (Center for Computational Structural Biology, The Scripps Research Institute, La Jolla, CA, USA).

Ligands preparation: The structures of ligands published by Chollet et al. [31] were downloaded from BindingDB [84], processed with the use of Open Babel [85] and manually revised in Avogadro 1.2. The compounds from Table 3 were modeled in Avogadro. The input files for docking were subsequently prepared with the use of the ADFR software suite.

Docking: Autodock Vina in version 1.2.3 was employed for the docking [86,87]. The Vina scoring function was used. For InhA, the center of the box for generating Vina grid was placed at the center of mass of the ligand in chain A of structure 4OYR; a box size of 50 × 55 × 50 grid points was used with the grid spacing of 0.375 Å. To ensure that the minimum energy docking pose is found correctly, the exhaustiveness of the search was increased from the default value.

Visualization and verification: The structures of complexes from docking were visualized with the use of PyMOL. Each time, only the pose with the best docking score was considered. The interactions were identified with the help of AutoDock Tools. To verify that the procedure correctly identifies the binding modes of ligands, the complexes from docking were compared to the available X-ray structures of InhA from RCSB PDB: 4OHU, 4OYR, 4OXY, 5COQ, 4BNN, 4OXK, and 3FNH. For this purpose, structural alignment in PyMOL was used, and Cα atoms of the protein were aligned.

## 4. Conclusions

A series of 2 × 16 anilides was synthesized from 3-(trifluoromethyl)cinnamic acid (series **1**) and 4-(trifluoromethyl)cinnamic acid (series **2**). All the compounds were subjected to extensive antimicrobial screening against Gram-positive bacteria and two mycobacterial strains. Of the prepared compounds, nine derivatives demonstrated activity. Substitution with CF_3_ in C_(3)_ of the cinnamic scaffold appears to be more favorable. Compounds **1j** (R^1^ = CF_3_, R^2^ = H, R^3^ = 4-CF_3_), **1o** (R^1^ = CF_3_, R^2^ = H, R^3^ = 3,5-Cl), **2i** (R^1^ = H, R^2^ = CF_3_, R^3^ = 3-CF_3_), and **2p** (R^1^ = H, R^2^ = CF_3_, R^3^ = 3,5-CF_3_) showed antistaphylococcal (MICs/MBCs 0.146–5.57 µM) as well as anti-enterococcal (MICs/MBCs 2.34–44.5 µM) activity. Compounds **1c** (R^1^ = CF_3_, R^2^ = H, R^3^ = 3-F), **1f** (R^1^ = CF_3_, R^2^ = H, R^3^ = 3-Cl), **1g** (R^1^ = CF_3_, R^2^ = H, R^3^ = 4-Cl), **1i** (R^1^ = CF_3_, R^2^ = H, R^3^ = 3-CF_3_), and **1p** (R^1^ = CF_3_, R^2^ = H, R^3^ = 3,5-CF_3_) expressed only antistaphylococcal activity. The growth of *M. marinum* was strongly inhibited by compounds **1j**, **1p**, **2i**, and **2p** in an MIC range from 0.29 to 2.34 µM, while all the agents of series **1** showed activity against *M. smegnatis* (MICs ranged from 9.36 to 51.7 µM). The compounds had a significant effect on inhibiting proliferation by modulating bacterial respiration as demonstrated by the MTT assay; compounds **1o** and **1j** reducing *S. aureus* viability by more than 97% at half or even a quarter of their MIC values were the most active. The compounds showed not only bacteriostatic activity but also bactericidal activity, which is very valuable. Two of the most potent compounds **1p** and **2p** possessed cytotoxic effect against THP-1 cancer cells (IC_50_ 6.7 and 8.9 µM), making them at least from **2p** remarkable anti-invasive agents with dual (cytotoxic and antibacterial) activity. The other compounds did show insignificant cytotoxic effect (IC_50_ > 10 µM and >30 µM), thus compounds **1j** and **1o** are the most interesting purely antibacterial compounds within the prepared molecules. In general, it can be stated that the antimicrobial activity increases with lipophilicity (optimum log *k* ~1.2) and with increasing electron-withdrawing properties of the anilide substituent. The antiproliferative effect on THP-1 cancer cells is associated with 3,5-CF_3_-disubstitution of anilide. The performed docking study, together with the similarity of our compounds with known InhA inhibitors, indicate that our compounds may target the mycobacterial enzyme InhA, which is involved in the biosynthesis of mycolic and other long-chain fatty acids contained in the mycobacterial cell wall, and thereby prevent the proliferation of mycobacteria.

## Figures and Tables

**Table 1:** Structures of ring-substituted (2*E*)-3-[3-(trifluoromethyl)phenyl]-*N*-arylprop-2-enanilides **1a**–**p**, (2*E*)-3-[4-(trifluoromethyl)phenyl]-*N*-arylprop-2-enanilides **2a**–**p**, experimentally determined (log *k*, log *D*_6.5_ and log *D*_7.4_), calculated lipophilicities (log *P*/Clog *P*) values of investigated compounds and electronic σ parameters of substituted anilide ring.

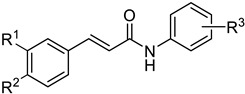										
No.	R^1^	R^2^	R^3^	log *k*	log *D*_6.5_	log *D*_7.4_	log *P* ^1^	log *P* ^2^	Clog *P* ^2^	σ_Ph-R3_ ^1^
**1a**	CF_3_	H	H	0.5234	0.4663	0.4633	3.93	4.10	4.5470	0.60
**1b**	CF_3_	H	2-F	0.5375	0.4804	0.4805	3.84	4.26	4.3476	1.02
**1c**	CF_3_	H	3-F	0.6333	0.5768	0.5775	4.19	4.26	4.9476	0.82
**1d**	CF_3_	H	4-F	0.5760	0.5178	0.5175	3.77	4.26	4.9476	0.62
**1e**	CF_3_	H	2-Cl	0.6568	0.6010	0.6012	4.56	4.66	4.6676	1.02
**1f**	CF_3_	H	3-Cl	0.8016	0.7477	0.7468	4.80	4.66	5.5176	0.85
**1g**	CF_3_	H	4-Cl	0.7982	0.7431	0.7413	4.67	4.66	5.5176	0.75
**1h**	CF_3_	H	2-CF_3_	0.5710	0.5135	0.5119	4.43	5.02	4.4308	0.91
**1i**	CF_3_	H	3-CF_3_	0.8807	0.8249	0.8287	5.29	5.02	5.8808	0.89
**1j**	CF_3_	H	4-CF_3_	0.9247	0.8706	0.8666	4.60	5.02	5.8808	0.95
**1k**	CF_3_	H	2,4-F	0.5906	0.5318	0.5326	3.99	4.42	4.5808	1.04
**1l**	CF_3_	H	3,5-F	0.8186	0.7657	0.7636	4.52	4.42	5.1808	1.12
**1m**	CF_3_	H	2,4-Cl	0.9782	0.9284	0.9279	5.41	5.22	5.4708	1.12
**1n**	CF_3_	H	2,5-Cl	0.9612	0.9096	0.9091	5.69	5.22	5.4708	1.22
**1o**	CF_3_	H	3,5-Cl	1.2028	1.1506	1.1488	5.68	5.22	6.3208	1.11
**1p**	CF_3_	H	3,5-CF_3_	1.3648	1.3126	1.3121	6.75	5.94	6.9216	1.05
**2a**	H	CF_3_	H	0.5351	0.4751	0.4684	3.96	4.10	4.5470	0.60
**2b**	H	CF_3_	2-F	0.5515	0.4924	0.4811	3.87	4.26	4.3476	1.02
**2c**	H	CF_3_	3-F	0.6462	0.5877	0.5750	4.23	4.26	4.9476	0.82
**2d**	H	CF_3_	4-F	0.5887	0.5283	0.5159	3.79	4.26	4.9476	0.62
**2e**	H	CF_3_	2-Cl	0.6614	0.6050	0.5917	4.60	4.66	4.6676	1.02
**2f**	H	CF_3_	3-Cl	0.8095	0.7526	0.7402	4.82	4.66	5.5176	0.85
**2g**	H	CF_3_	4-Cl	0.8054	0.7483	0.7351	4.70	4.66	5.5176	0.75
**2h**	H	CF_3_	2-CF_3_	0.5798	0.5187	0.5081	4.46	5.02	4.4308	0.91
**2i**	H	CF_3_	3-CF_3_	0.8867	0.8300	0.8177	5.33	5.02	5.8808	0.89
**2j**	H	CF_3_	4-CF_3_	0.9319	0.8743	0.8617	4.63	5.02	5.8808	0.95
**2k**	H	CF_3_	2,4-F	0.6011	0.5396	0.5225	4.01	4.42	4.5808	1.04
**2l**	H	CF_3_	3,5-F	0.8253	0.7678	0.7516	4.55	4.42	5.1808	1.12
**2m**	H	CF_3_	2,4-Cl	0.9735	0.9196	0.9033	5.44	5.22	5.4708	1.12
**2n**	H	CF_3_	2,5-Cl	0.9583	0.9037	0.8903	5.72	5.22	5.4708	1.22
**2o**	H	CF_3_	3,5-Cl	1.2024	1.1511	1.1440	5.72	5.22	6.3208	1.11
**2p**	H	CF_3_	3,5-CF_3_	1.3665	1.3169	1.3098	6.80	5.94	6.9216	1.05

^1^ ACD/Percepta ver. 2012 (Advanced Chemistry Development. Inc., Toronto, ON, Canada, 2012); ^2^ ChemBioDrawUltra 13.0 (CambridgeSoft, PerkinElmer Inc., Waltham, MA, USA).

**Table 2:** In vitro antistaphylococcal, anti-enterococcal, and antimycobacterial activities (MIC/MBC [μM]) compared to ampicillin (AMP), isoniazid (INH), and in vitro cell viability (IC_50_ [µM] ± SD, *n* = 6) on human monocytic leukemia cell line (THP-1).

No.	MIC [µM] MBC [µM]										IC_50_ [µM] THP-1 24 h
	SA	MRSA1	MRSA2	MRSA3	EF	VRE1	VRE2	VRE3	MM	MS	
**1c**	12.9 **25.9**	25.9 **25.9**	12.9 **12.9**	12.9 **12.9**	n.a. –	n.a. –	n.a. –	n.a. –	n.a. –	51.7 –	>30
**1f**	12.3 **12.3**	6.14 **6.14**	6.14 **6.14**	6.14 **12.3**	n.a. –	n.a. –	n.a. –	n.a. –	n.a. –	24.6 –	>10
**1g**	24.6 **49.1**	12.3 **24.6**	6.14 **6.14**	12.3 **24.6**	n.a. –	n.a. –	n.a. –	n.a. –	n.a. –	24.6 –	>10
**1i**	11.1 **22.3**	11.1 **11.1**	2.78 **2.78**	2.78 22.3	n.a. –	n.a. –	n.a. –	n.a. –	n.a. –	11.1 –	>10
**1j**	2.78 **2.78**	2.78 **2.78**	1.39 **1.39**	2.78 **5.57**	22.3 **22.3**	22.3 **22.3**	44.5 **44.5**	22.3 **22.3**	0.348 –	11.1 –	>30
**1o**	0.694 **0.694**	1.39 **2.78**	0.694 **0.694**	0.694 **1.39**	11.1 **22.2**	22.2 **22.2**	22.2 **22.2**	22.2 **22.2**	22.2 –	11.1 –	>30
**1p**	1.17 **1.17**	2.34 **2.34**	1.17 **1.17**	0.585 **1.17**	n.a. –	n.a. –	n.a. –	n.a. –	2.34 –	9.36 –	6.7 ± 1.1
**2i**	1.39 **1.39**	2.78 **2.78**	1.39 **1.39**	2.78 **2.78**	11.1 **11.1**	11.1 **22.3**	11.1 **44.5**	11.1 **11.1**	2.78 –	n.a. –	>10
**2p**	0.146 **0.146**	0.585 **0.585**	0.293 **0.585**	0.293 **0.293**	2.34 **4.68**	2.34 **4.68**	4.68 **9.36**	2.34 **2.34**	0.293 –	1.17 –	8.9 ± 1.3
**AMP**	5.72 >5.72	45.8 >45.8	45.8 >45.8	45.8 >45.8	2.81 2.81	11.5 11.5	11.5 11.5	11.5 11.5	–	–	–
**INH**	–	–	–	–	–	–	–	–	36.6 –	117 –	–

SA *= Staphylococcus aureus* ATCC 29213; MRSA1–3 *=* clinical isolates of methicillin-resistant *S. aureus* SA 3202, SA 630 (National Institute of Public Health, Prague, Czech Republic), and 63718 (Department of Infectious Diseases and Microbiology, Faculty of Veterinary Medicine, University of Veterinary Sciences Brno, Czech Republic); EF = *Enterococcus faecalis* ATCC 29213, and vancomycin-resistant enterococci VRE1–3 = VRE 342B, VRE 368, VRE 725B, MM = *Mycobacterium marinum* CAMP 5644; MS = *M. smegmatis* ATCC 700084; FSB = fetal bovine serum; n.a. = no activity. The real bactericidal values required by the MBC/MIC ≤ 4 rule are in bold.

**Table 3:** Lowest MIC values with at least 70% inhibition of *S. aureus* ATCC 29213 respiratory activity.

No.	Conc.	*S. aureus* Respiration Inhibition (%)
**1c**	2× MIC (1× MBC)	97.9
**1f**	1× MIC (1× MBC)	96.0
**1g**	4× MIC (2× MBC)	71.1
**1i**	1× MIC (0.5× MBC)	98.0
**1j**	0.5× MIC (0.5× MBC)	97.9
**1o**	0.25× MIC (0.25× MBC)	97.8
**1p**	1× MIC (1× MBC)	97.7
**2i**	1× MIC (1× MBC)	98.1
**2p**	2× MIC (2× MBC)	98.0
**AMP**	8× MIC (>8× MBC)	90.0
**CPX**	32× MIC (32× MBC)	92.8

## Data Availability

The data presented in this study are available on request from the corresponding author.

## Supplementary Materials

The supporting information can be downloaded at: https://www.mdpi.com/article/10.3390/ijms232315090/s1.
