# Supplementary material for: Trifluoromethylcinnamanilide Michael Acceptors for Treatment of Resistant Bacterial Infections
Source: Int J Mol Sci. 2022 Dec 1;23(23):15090. doi: 10.3390/ijms232315090 (PMC9737391; doi:10.3390/ijms232315090)
Supplement: Supplementary file 1 [file ijms-23-15090-s001.zip › ijms-2035571-supplementary.pdf]

## Supplementary Materials

# Trifluoromethylcinnamanilide Michael Acceptors for Treatment of Resistant Bacterial Infections

**Tomas Strharsky [1,2], Dominika Pindjakova [3], Jiri Kos [3,4,*], Lucia Vrablova [3], Pavel Smak [4], Hana Michnova [5], Tomas Gonec [1], Jan Hosek [6], Michal Oravec [7], Izabela Jendrzejewska [8], Alois Cizek [5] and Josef Jampilek [3,9]**

[1] Department of Chemical Drugs, Faculty of Pharmacy, Masaryk University, Palackeho 1946/1, 612 00 Brno, Czech Republic; strharsky.t@gmail.com (T.S.); t.gonec@seznam.cz (T.G.)
[2] Regional Centre of Advanced Technologies and Materials, Czech Advanced Technology and Research Institute, Palacky University, Slechtitelu 27, 783 71 Olomouc, Czech Republic
[3] Department of Analytical Chemistry, Faculty of Natural Sciences, Comenius University, Ilkovicova 6, 842 15 Bratislava, Slovakia; pindjakova.dominika@gmail.com (D.P.); lucia.vrablova26@gmail.com (L.V.); josef.jampilek@gmail.com (J.J.)
[4] Department of Biochemistry, Faculty of Medicine, Masaryk University, Kamenice 5, Brno 625 00, Czech Republic; pavel.smak@gmail.com (P.S.)
[5] Department of Infectious Diseases and Microbiology, Faculty of Veterinary Medicine, University of Veterinary Sciences Brno, Palackeho tr. 1946/1, 612 42 Brno, Czech Republic; michnova.hana@gmail.com (H.M.); cizeka@vfu.cz (A.C.)
[6] Department of Pharmacology and Toxicology, Veterinary Research Institute, Hudcova 296/70, 621 00 Brno, Czech Republic; jan.hosek@vri.cz
[7] Global Change Research Institute CAS, Belidla 986/4a, 603 00 Brno, Czech Republic; oravec.m@czechglobe.cz
[8] Institute of Chemistry, University of Silesia in Katowice, 40-007 Katowice, Poland; izabela.jendrzejewska@us.edu.pl
[9] Institute of Neuroimmunology, Slovak Academy of Sciences, Dubravska Cesta 9, 845 10 Bratislava, Slovakia
* Correspondence: jirikos85@gmail.com

## 1. Docking of Known InhA Ligands

With the studied ligands from Chollet et al. [1], two distinct conformations of the InhA binding site are available. In the article, these are referred to as open and closed conformation. We chose to proceed with the closed conformation for docking since the ligands observed with this conformation have higher structural similarity to our compounds. The structure with PDB ID 4OYR was prepared as a receptor for docking, and the ligands were retrieved from the BindingDB. InhA shows two binding sites: one for nicotinamide adenine dinucleotide (NAD), the other for another smaller ligand. The ligands were docked into the binding site of the smaller ligand, and the NAD was left as a part of the receptor.

Table S1 shows the comparison between X-ray structures and complexes from the rigid receptor docking algorithm. Only ligands of similar size to our compounds having available X-ray structures were used. For each complex, structural alignment of the receptor and protein from the X-ray structure was performed, and the complexes were visualized in PyMOL. The comparison indicates that the docking algorithm can reliably predict the correct binding mode for a tested set of ligands.

**Table S1.** Comparison between binding mode from docking and X-ray structures. Receptor and ligand from docking are shown in green and cyan, respectively, and X-ray structure is shown in magenta.

51030557 and PDB ID 4OHU (chain A)

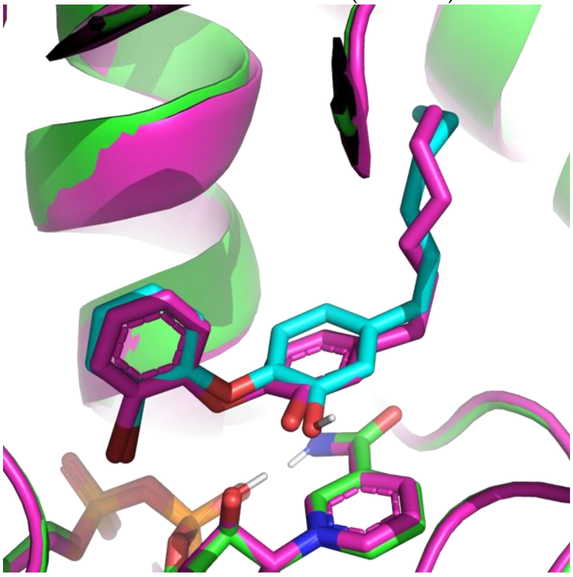

51030560 and PDB ID 4OYR (chain B)

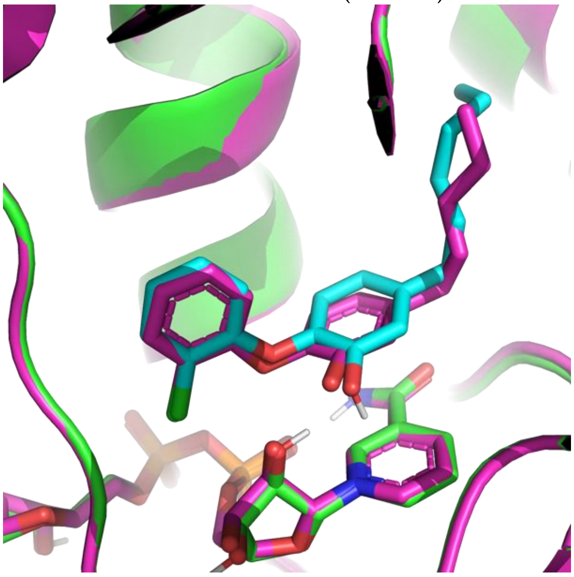

51030559 and PDB ID 4OXY (chain A)

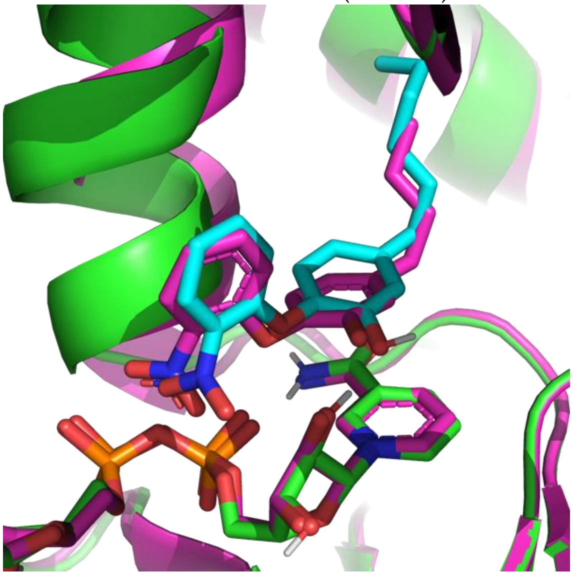

51030539 and PDB ID 5COQ (chain A)

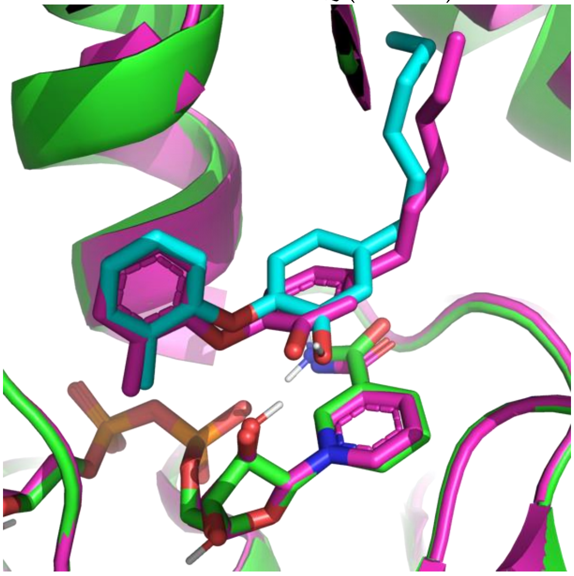

51030556 and PDB ID 4BNN (chain A)

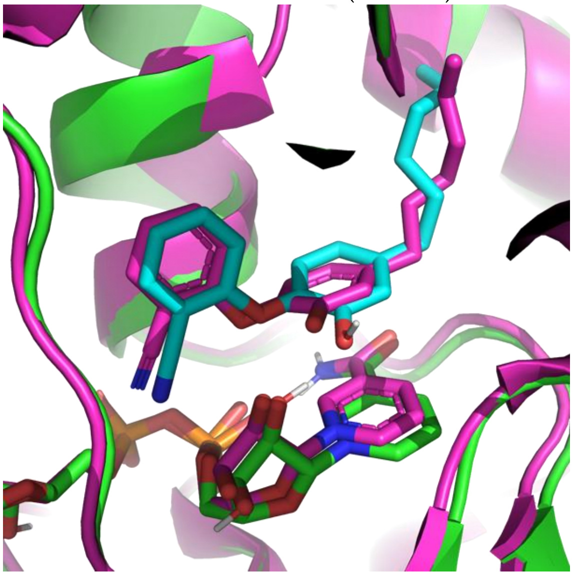

51030367 and PDB ID 4OXK (chain A)

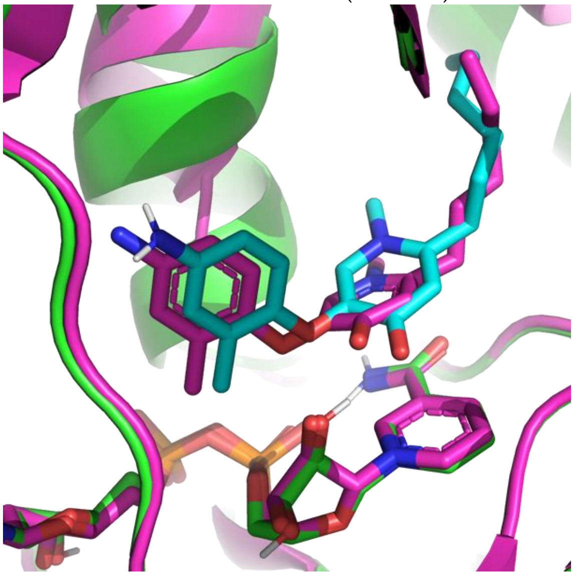

51030555 and PDB ID 3FNH (chain A)

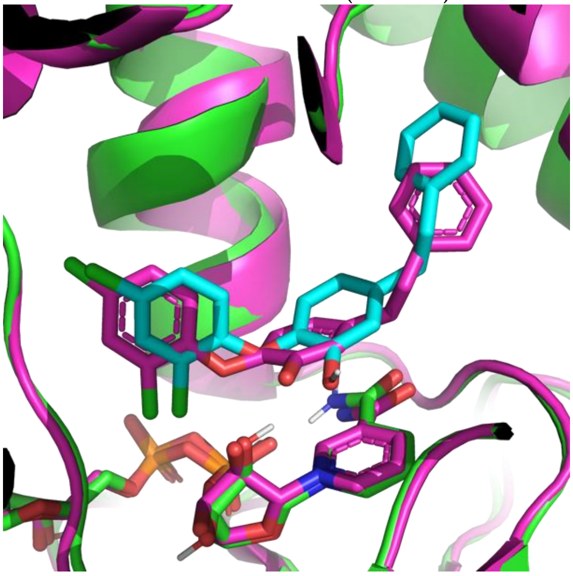

The obtained docking scores were compared to the experimental values of $K_i$. The comparison is shown in Figure S1 and Table S2. Only the ligands of similar size to our compounds were included. Furthermore, the ligands containing -N=N- or $NO_2$ group in their structure were excluded due to a poor parametrization of these structural features in the used force field. The figure shows a qualitative agreement between experimental values of log(Ki) and vina docking score. There are, however, four outliers (highlighted in orange). Within the preparation of the structures for docking, the hydrogens on nonpolar groups are removed, and the charges are summed to the carbon, which partially compensates for protein flexibility. Nonetheless, it also conceals the size difference between, e.g., a halogenide substituent and a methyl group. In our study, this is the case with ligands 51030362, 51030365, 51030543, which is demonstrated in Figure S2: ligand 51030362 has a better docking score than ligand 51030560. However, according to the experimental data, InhA shows a higher affinity to ligand 51030560.

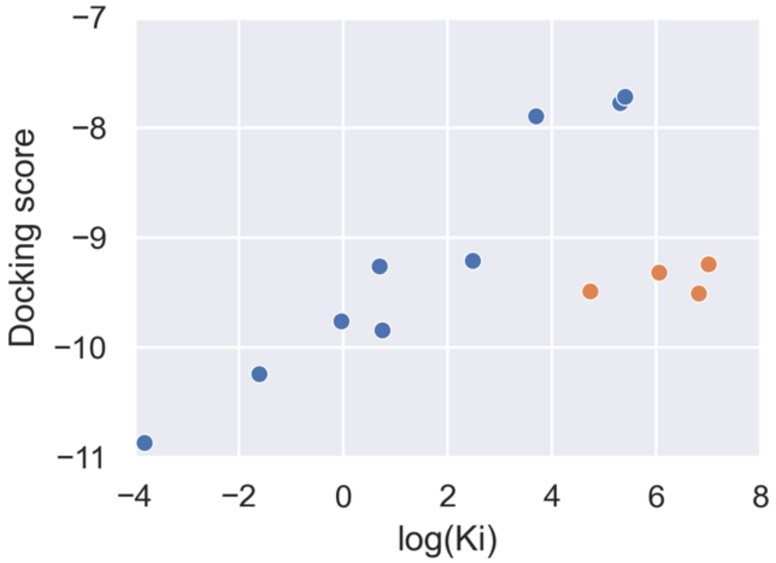


**Figure S1.** Correlation between the vina docking score and experimental Ki [1]. Ligands with outlying docking scores are shown in orange. These are 51030362, 51030365, 51030543, and 51030553.

**Table S2.** Experimental data for docked ligands [1] and their docking scores.

| BindingDB ID | Ki (nM) | log(Ki) | Docking score |
|---|---|---|---|
| **51030361** | 200.000 | 5.30 | -7.8 |
| **51030362** | 114.000 | 4.74 | -9.5 |
| **51030365** | 913.000 | 6.82 | -9.5 |
| **51030367** | 40.000 | 3.69 | -7.9 |
| **51030369** | 2.000 | 0.69 | -9.3 |
| **51030539** | 0.022 | -3.82 | -10.9 |
| **51030543** | 427.000 | 6.06 | -9.3 |
| **51030550** | 220.000 | 5.39 | -7.7 |
| **51030552** | 12.000 | 2.48 | -9.2 |
| **51030553** | 1100.000 | 7.00 | -9.2 |
| **51030556** | 2.100 | 0.74 | -9.8 |
| **51030557** | 0.200 | -1.61 | -10.2 |
| **51030560** | 0.960 | -0.04 | -9.8 |

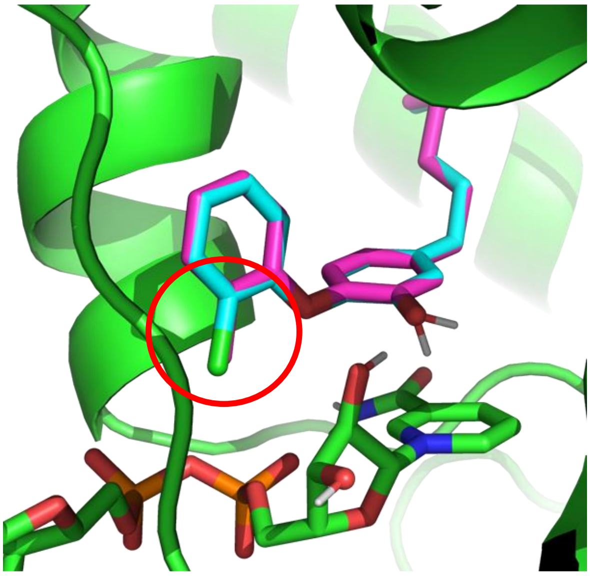

**Figure S2.** Comparison of ligands 51030560 (in cyan) and 51030362 (in magenta) docked into the binding site of InhA (in green). The latter ligand features a methyl group instead of a chloride group (in the circle), otherwise, their binding mode is identical.

## 2. Docking of our compounds

The docking scores for our compounds (complexes presented in Figures 5–7 of the main text) are provided in Table S3. The comparison between the binding mode of our compounds and triclosan [2] is shown in Figure S3.

**Table S3.** Docking scores for our compounds (complexes presented in Figures 5-7 of the main text).

| Compound ID | Docking score |
|---|---|
| **1c** | -9.833 |
| **1f** | -9.707 |
| **1g** | -9.735 |
| **1i** | -10.3 |
| **1j** | -10.39 |
| **1o** | -10.21 |
| **1p** | -9.921 |
| **2i** | -10.3 |
| **2p** | -9.573 |

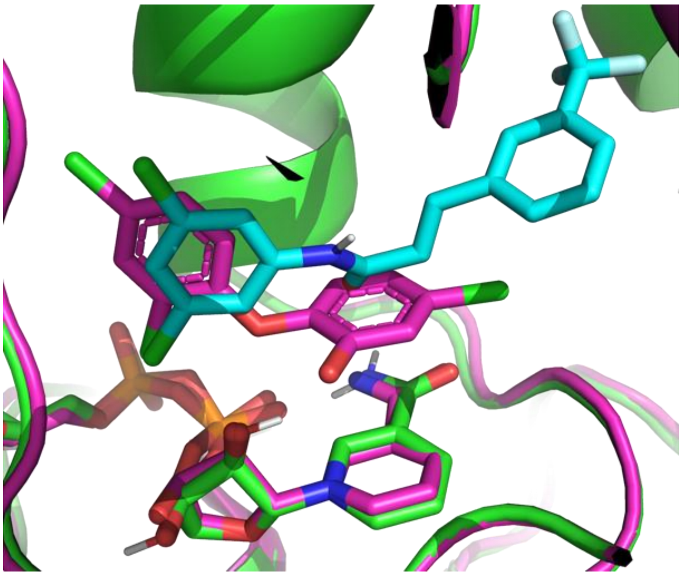

**Figure S3.** Superposition of compound **1o** (cyan) docked into the binding site of InhA with NAD (green) and triclosan in X-ray structure 2B35 (magenta). Structural alignment of the proteins was performed.

## 3. Structural comparison of subunit c of ATP synthase obtained from the different experiments

The structures of three available structures of subunit c of ATP synthase complex with antituberculotic drug bedaquiline (TMC207) [3] were mutually compared (Figure S4). The comparison shows a high structural similarity between the two EM structures (PDB ID 7NJV and 7JGC) and the X-ray structure (PDB ID 4V1F). Also, all three structures show the same binding mode of bedaquiline.

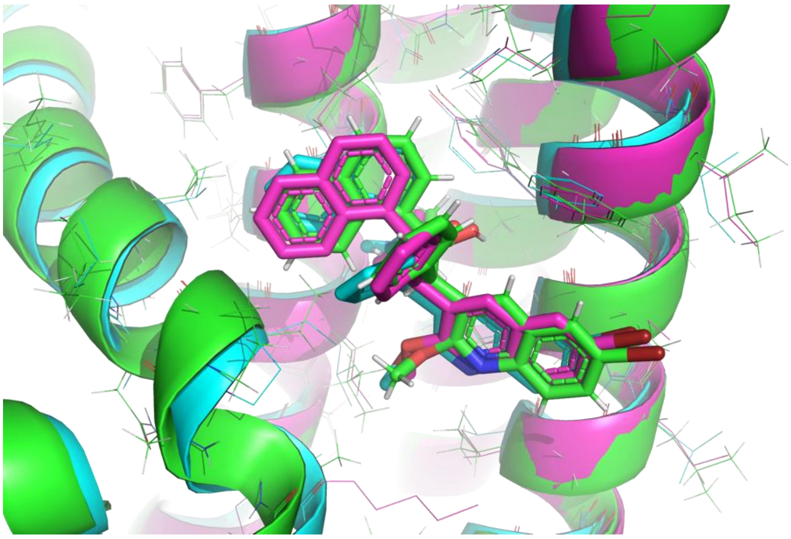

**Figure S4.** Comparison of the structure of ATP synthase subunit c complexes with bedaquiline; PDB ID 7NJV is shown in green, 7JGC in cyan, and 4V1F in magenta.

## References


1. Chollet, A.; Maveyraud, L.; Lherbet, C.; Bernardes-Genisson, V. An overview on crystal structures of inha protein: Apo-form, in complex with its natural ligands and inhibitors. *Eur. J. Med. Chem.* **2018**, *146*, 318–343.
2. Sullivan, T.J.; Truglio, J.J.; Boyne, M.E.; Novichenok, P.; Zhang, X.; Stratton, C.F.; Li, H. J.; Kaur, T.; Amin, A.; Johnson, F.; et al. High Affinity InhA inhibitors with activity against drug-resistant strains of Mycobacterium tuberculosis. *ACS Chem. Biol.* **2006**, 1, 43–53.
3. Koul, A.; Dendouga, N.; Vergauwen, K.; Molenberghs, B.; Vranckx, L.; Willebrords, R.; Ristic, Z.; Lill, H.; Dorange, I.; Guillemont, J.; et al. Diarylquinolines target subunit c of mycobacterial ATP synthase. *Nat. Chem. Biol.* **2007**, *3*, 323–324.

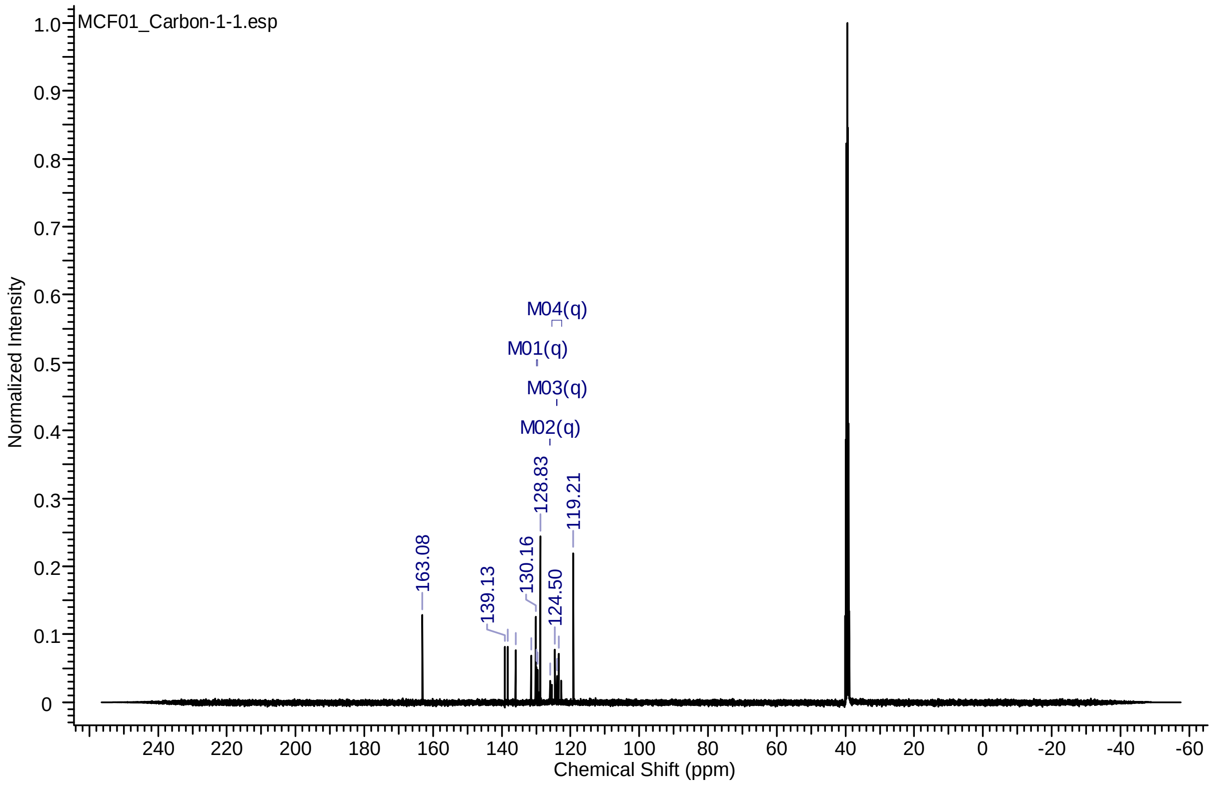


**Figure S5.** $^{13}$C-NMR (DMSO-$d_6$) spectrum of (2*E*)-*N*-phenyl-3-[3-(trifluoromethyl)phenyl]prop-2-enamide (**1a**).

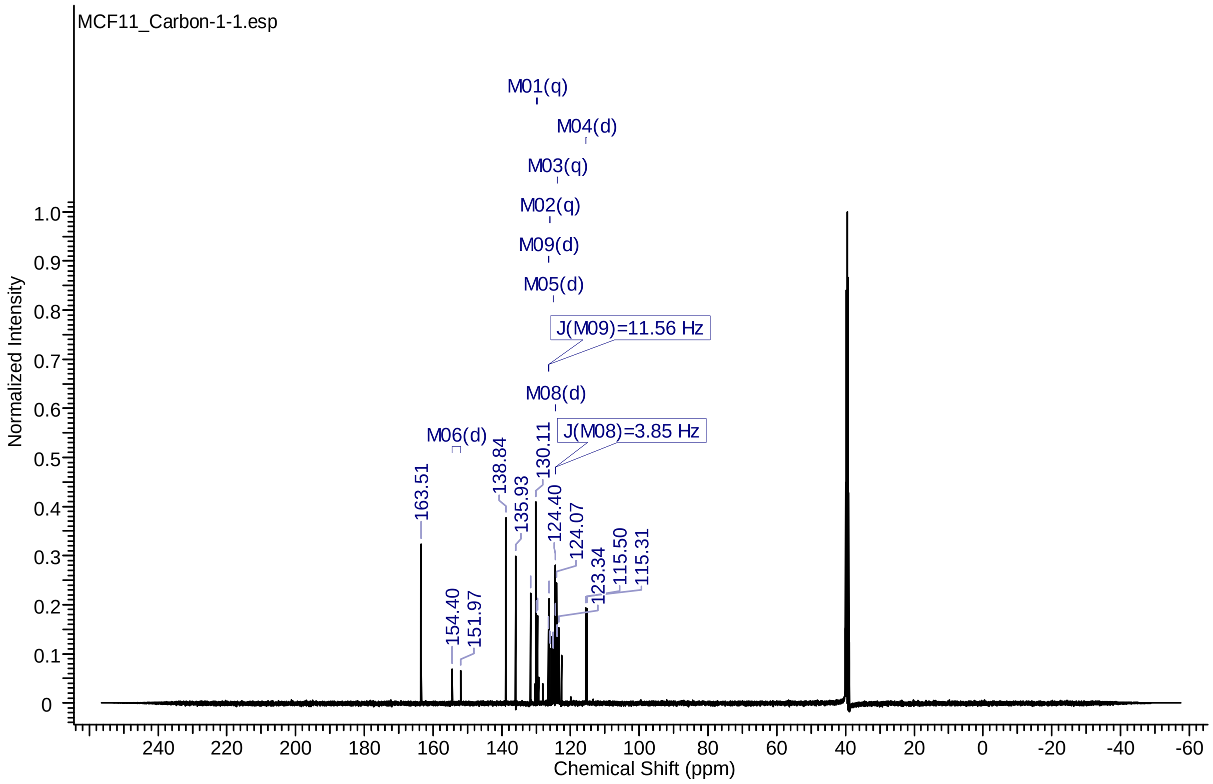


**Figure S6.** $^{13}$C-NMR (DMSO-$d_6$) spectrum of (2*E*)-*N*-(2-fluorophenyl)-3-[3-(trifluoromethyl)phenyl]prop-2-enamide (**1b**).

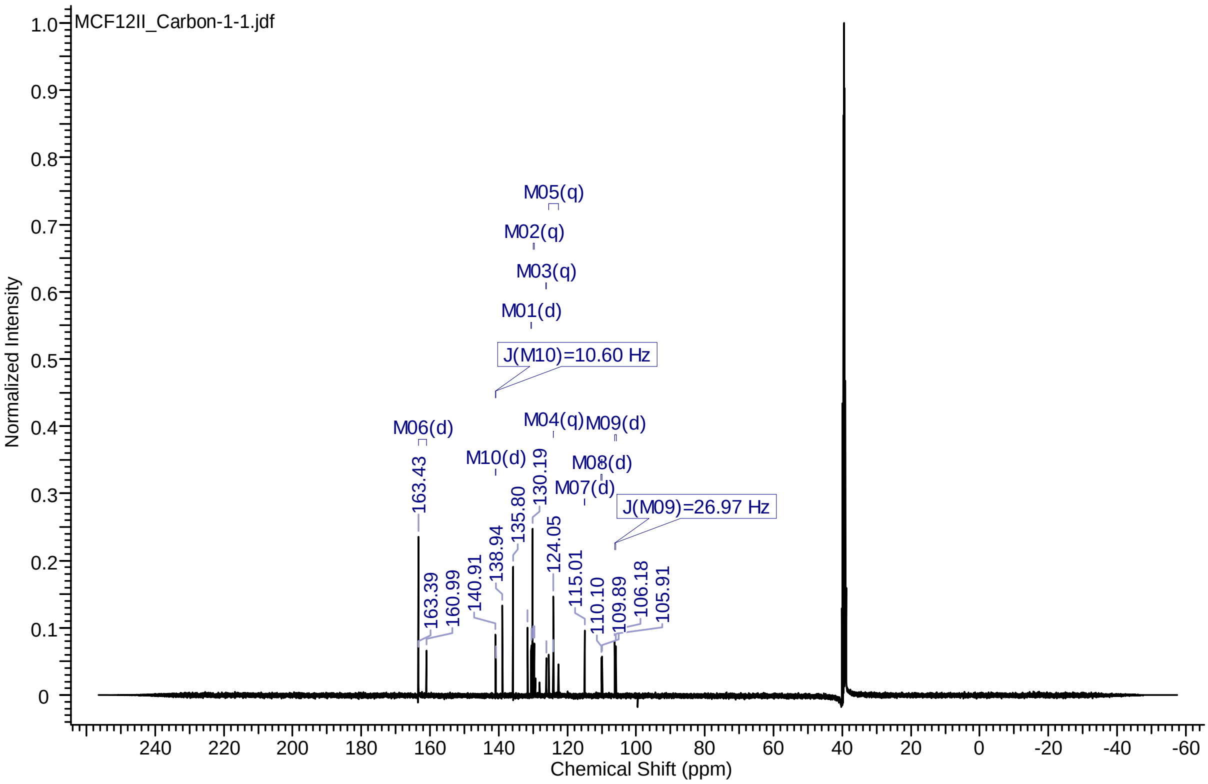


**Figure S7.** $^{13}$C-NMR (DMSO-$d_6$) spectrum of (2*E*)-*N*-(3-fluorophenyl)-3-[3-(trifluoromethyl)phenyl]prop-2-enamide (**1c**).

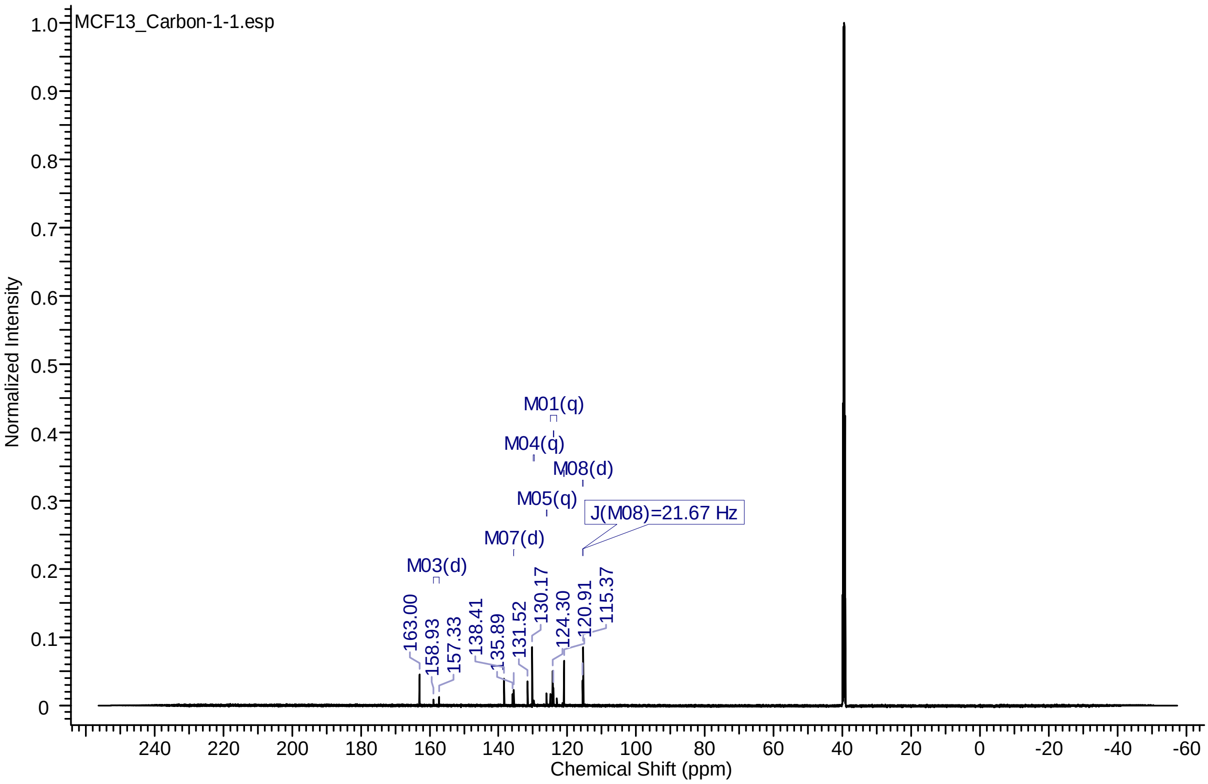


**Figure S8.** $^{13}$C-NMR (DMSO-$d_6$) spectrum of (2*E*)-*N*-(4-fluorophenyl)-3-[3-(trifluoromethyl)phenyl]prop-2-enamide (**1d**).

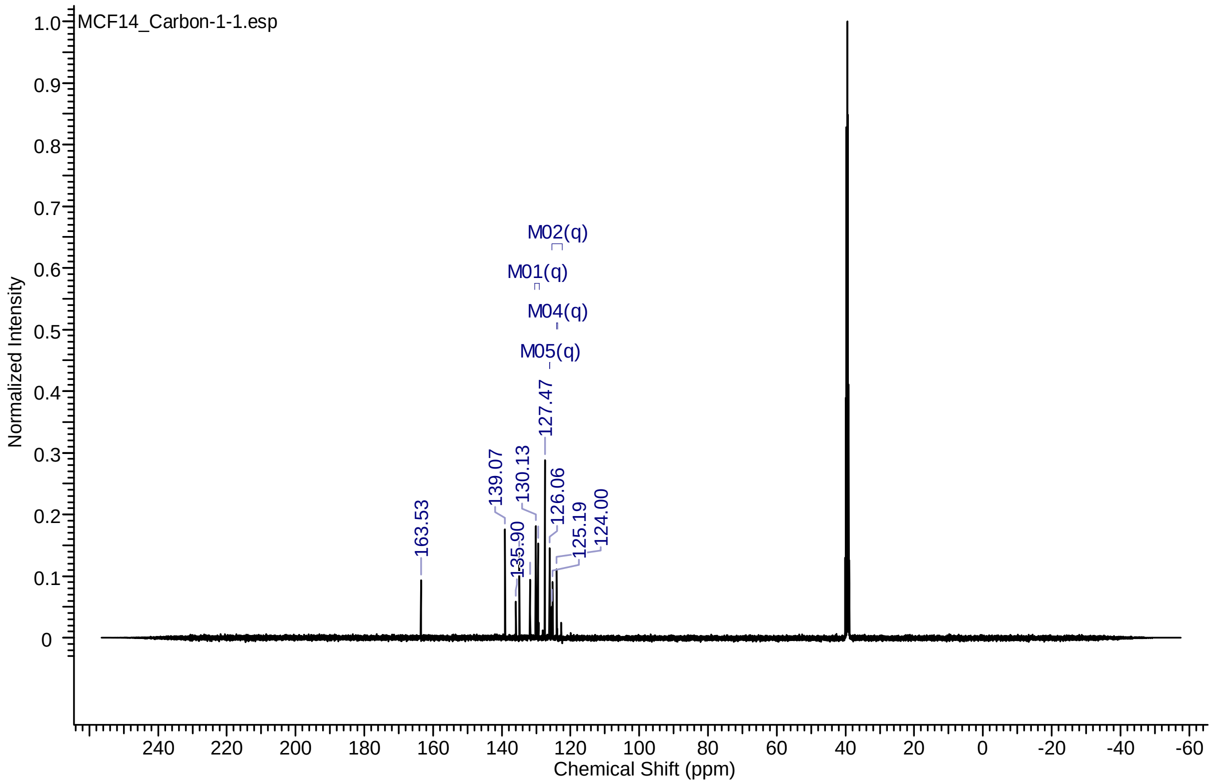


**Figure S9.** $^{13}$C-NMR (DMSO-$d_6$) spectrum of (2*E*)-*N*-(2-chlorophenyl)-3-[3-(trifluoromethyl)phenyl]prop-2-enamide (**1e**).

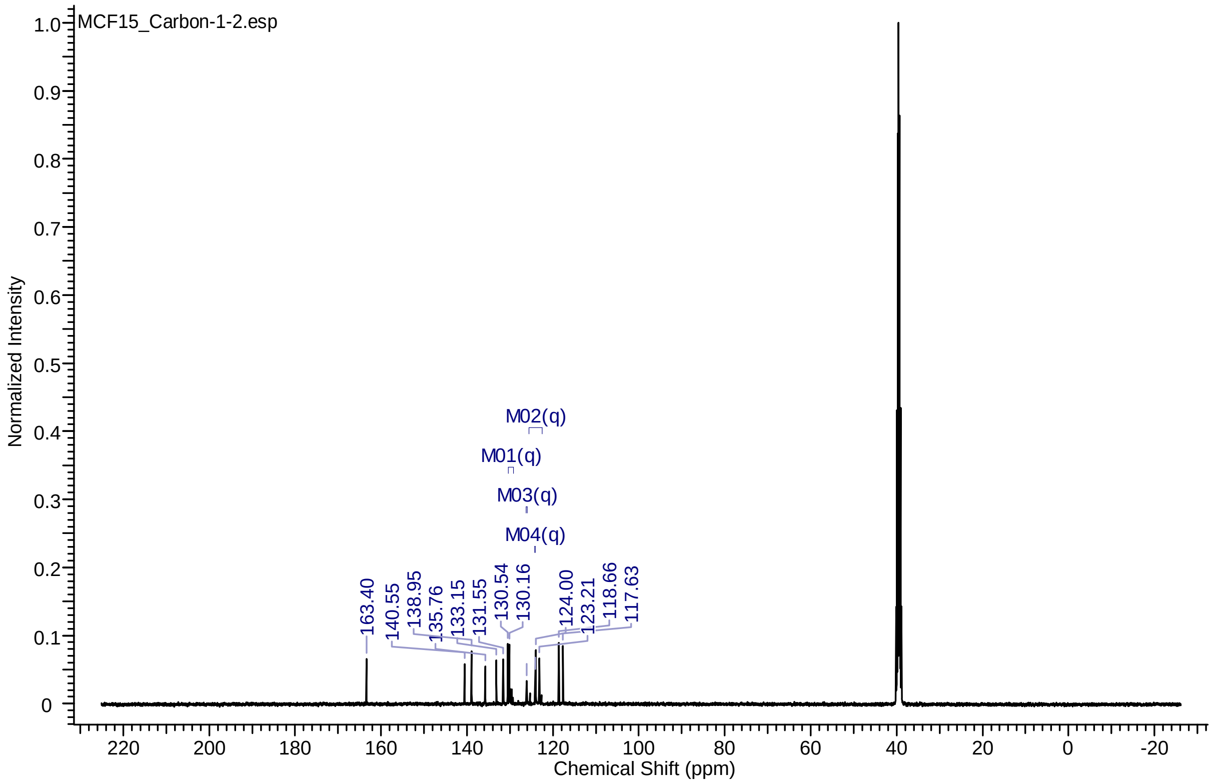


**Figure S10.** $^{13}$C-NMR (DMSO-d$_6$) spectrum of (2*E*)-*N*-(3-chlorophenyl)-3-[3-(trifluoromethyl)phenyl]prop-2-enamide (**1f**).

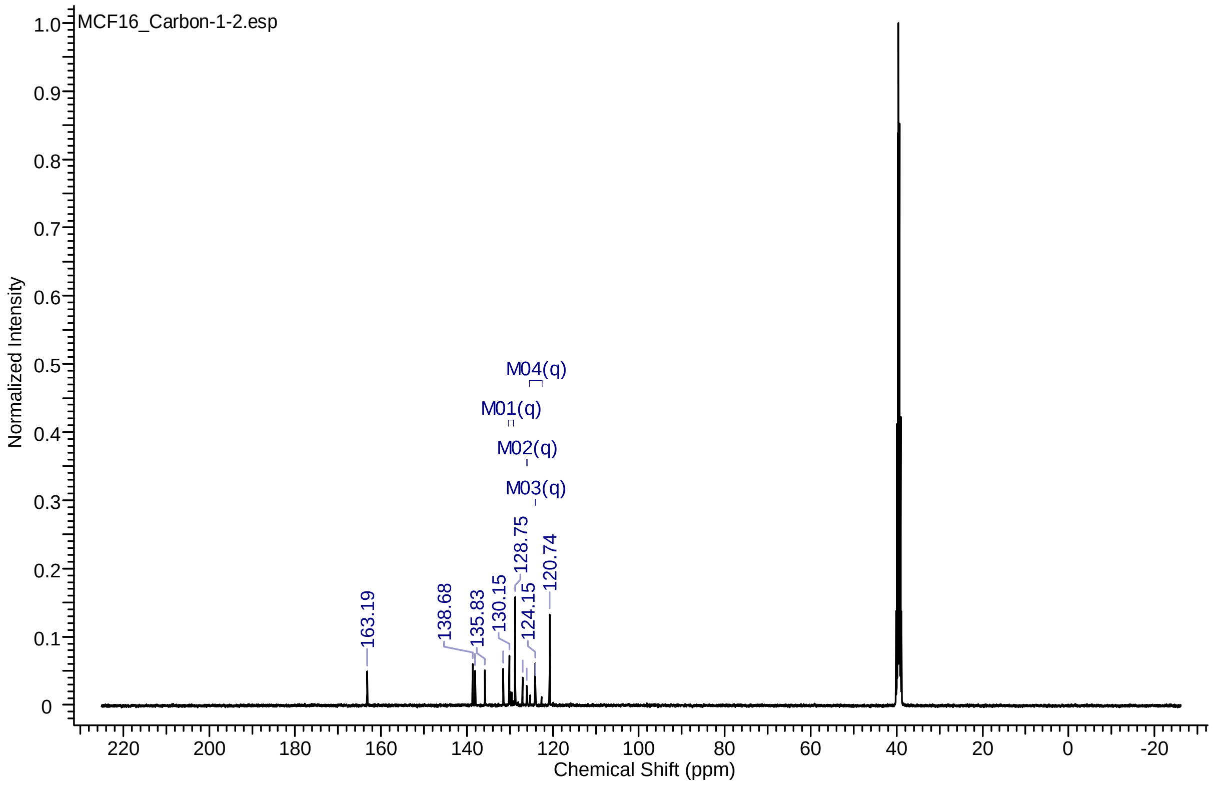


**Figure S11.** $^{13}$C-NMR (DMSO-d6) spectrum of (2*E*)-*N*-(4-chlorophenyl)-3-[3-(trifluoromethyl)phenyl]prop-2-enamide (**1g**).

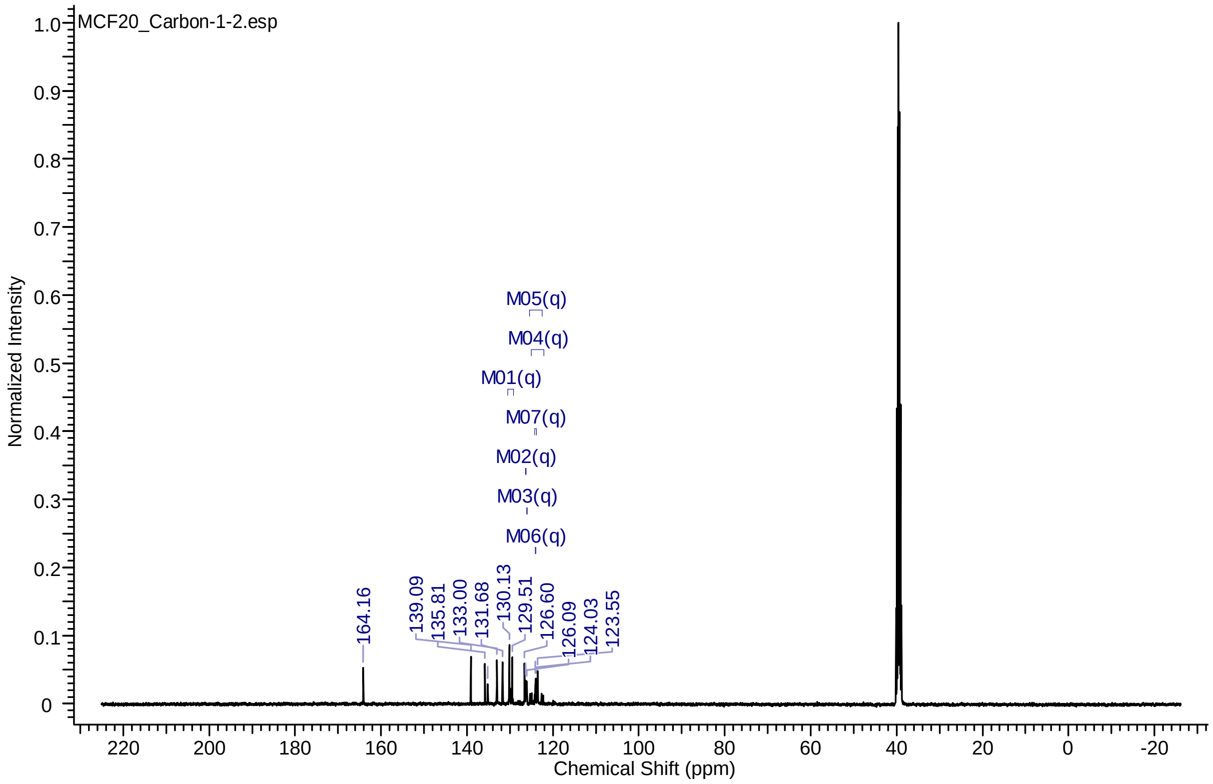


**Figure S12.** $^{13}$C-NMR (DMSO-d6) spectrum of (2*E*)-*N*-[2-(trifluoromethyl)phenyl]-3-[3-(trifluoromethyl)phenyl]prop-2-enamide (**1h**).

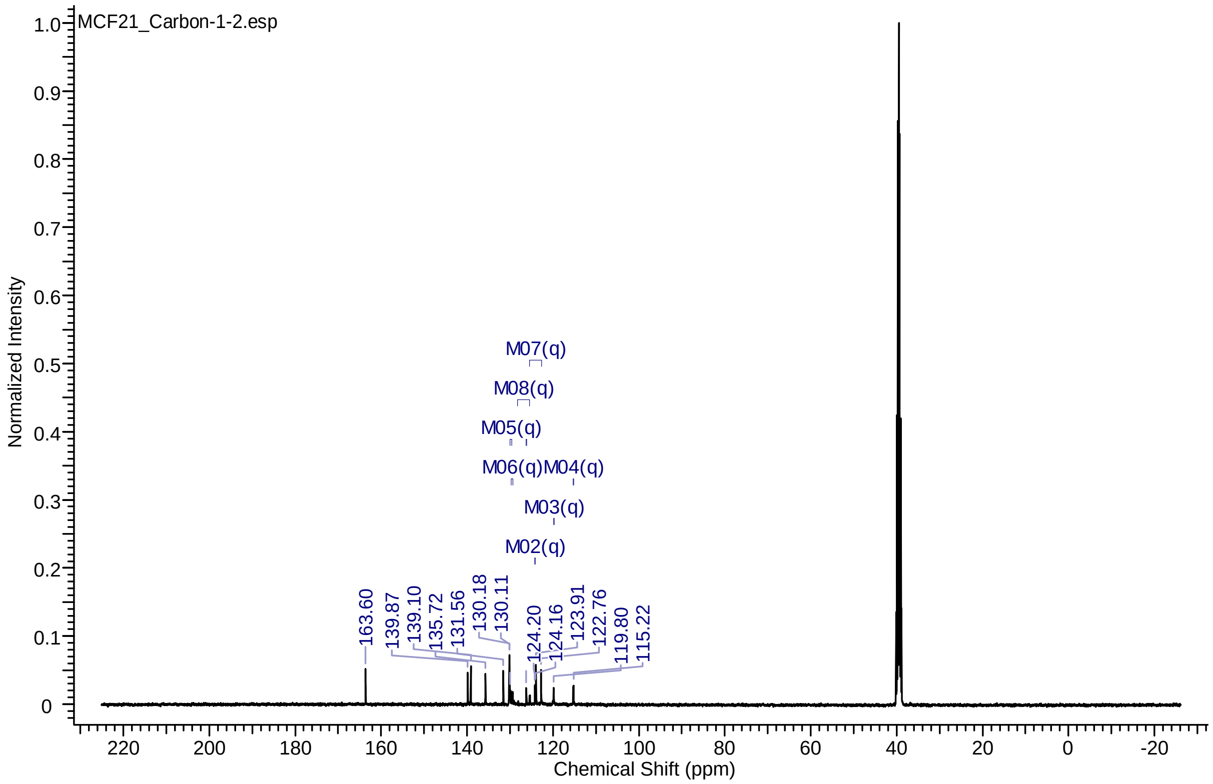


**Figure S13.** $^{13}$C-NMR (DMSO-d$_6$) spectrum of (2*E*)-*N*,3-bis[3-(trifluoromethyl)phenyl]prop-2-enamide (**1i**).

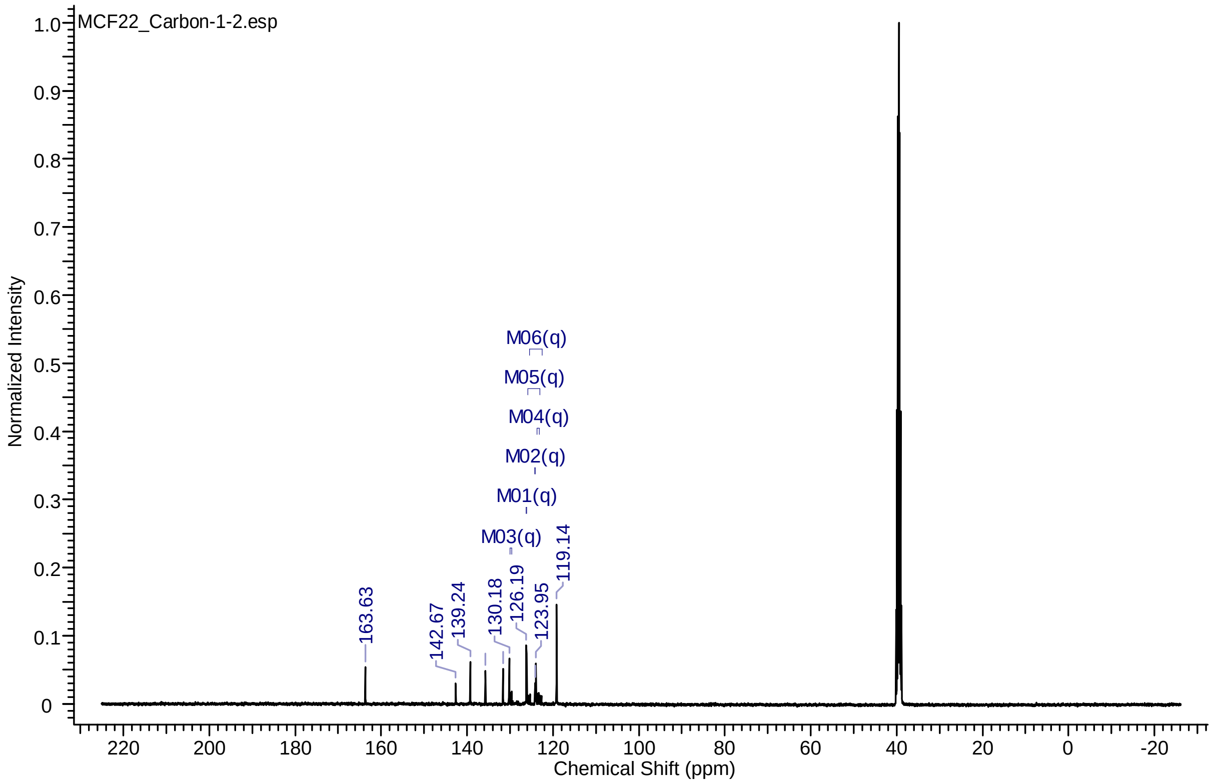


**Figure S14.** $^{13}$C-NMR (DMSO-d$_6$) spectrum of (2*E*)-3-[3-(trifluoromethyl)phenyl]-*N*-[4-(trifluoromethyl)phenyl]prop-2-enamide (**1j**).

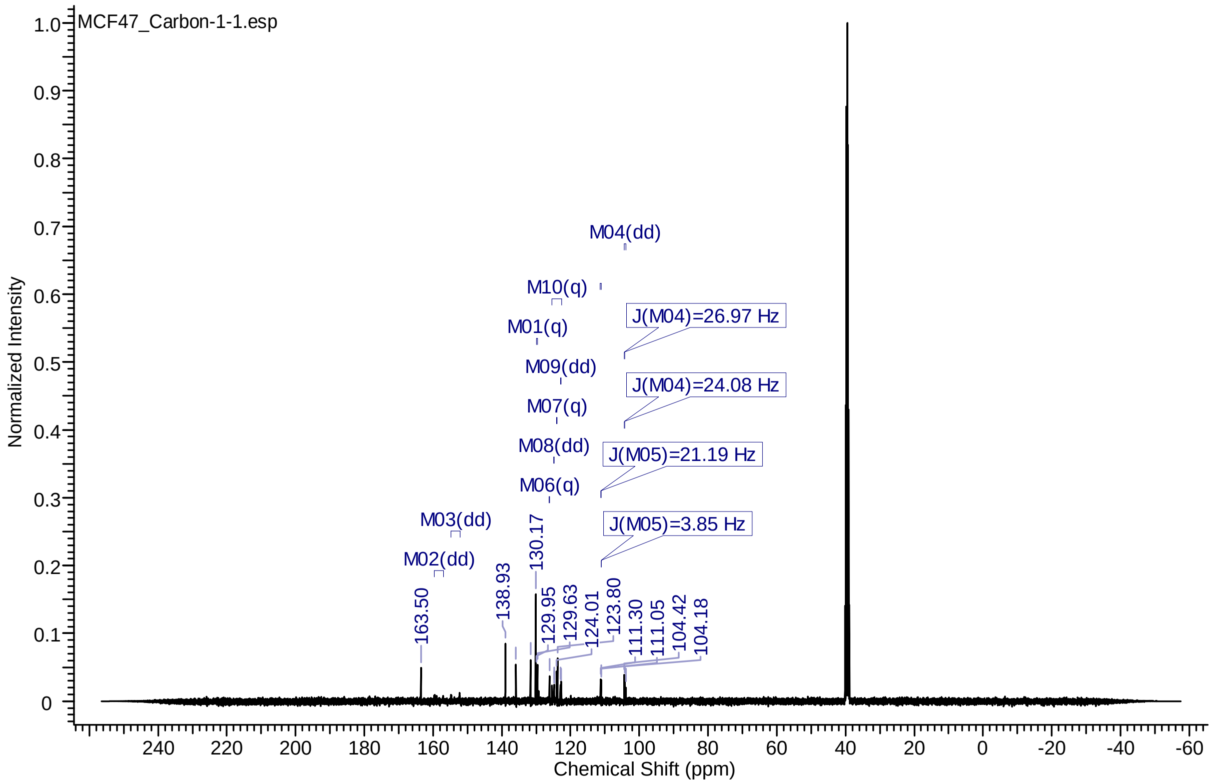


**Figure S15.** $^{13}$C-NMR (DMSO-d$_6$) spectrum of (2*E*)-*N*-(2,4-difluorophenyl)-3-[3-(trifluoromethyl)phenyl]prop-2-enamide (**1k**).

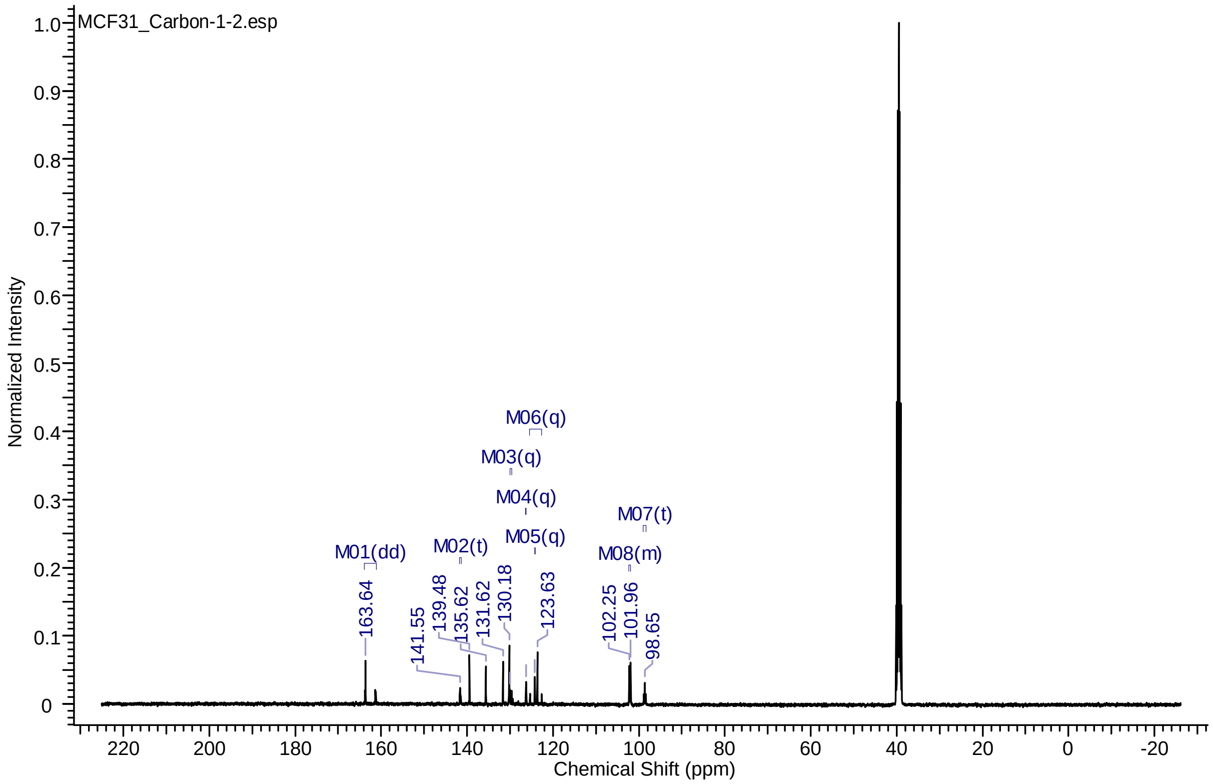


**Figure S16.** $^{13}$C-NMR (DMSO-d$_6$) spectrum of (2*E*)-*N*-(3,5-difluorophenyl)-3-[3-(trifluoromethyl)phenyl]prop-2-enamide (**1l**).

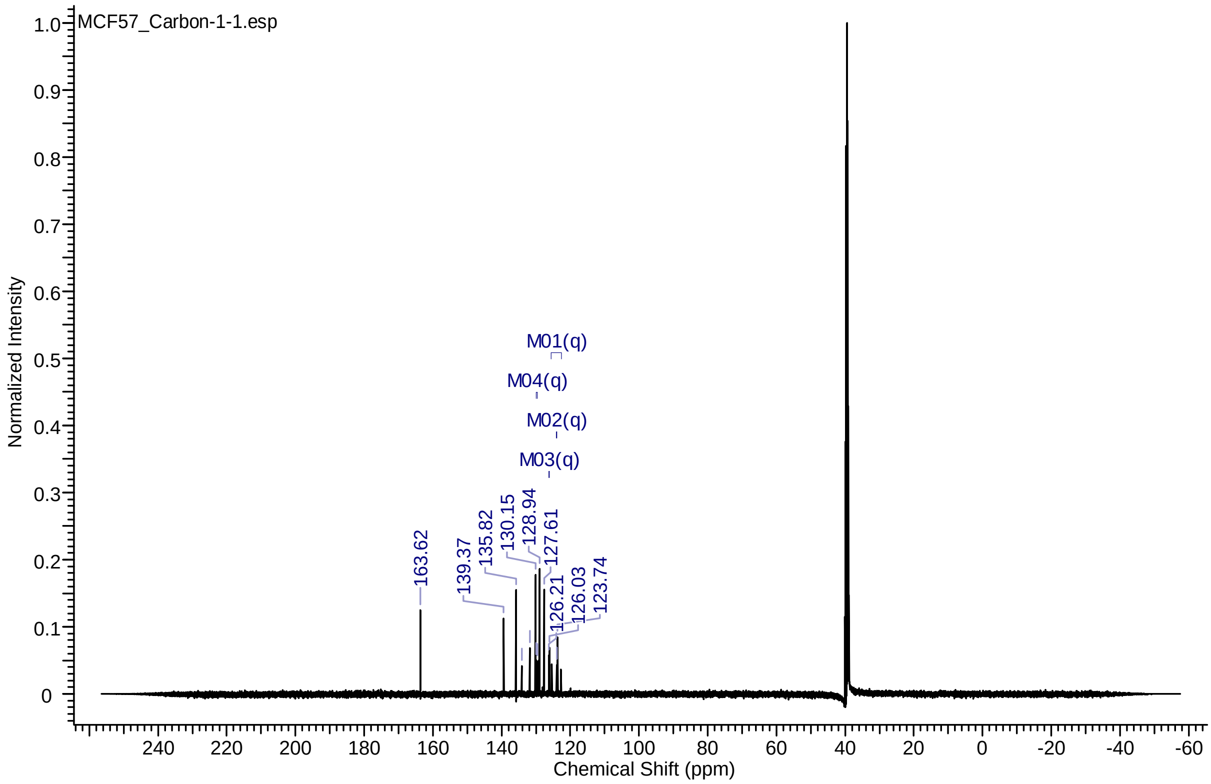


**Figure S17.** $^{13}$C-NMR (DMSO-d$_6$) spectrum of (2*E*)-*N*-(2,4-dichlorophenyl)-3-[3-(trifluoromethyl)phenyl]prop-2-enamide (**1m**).

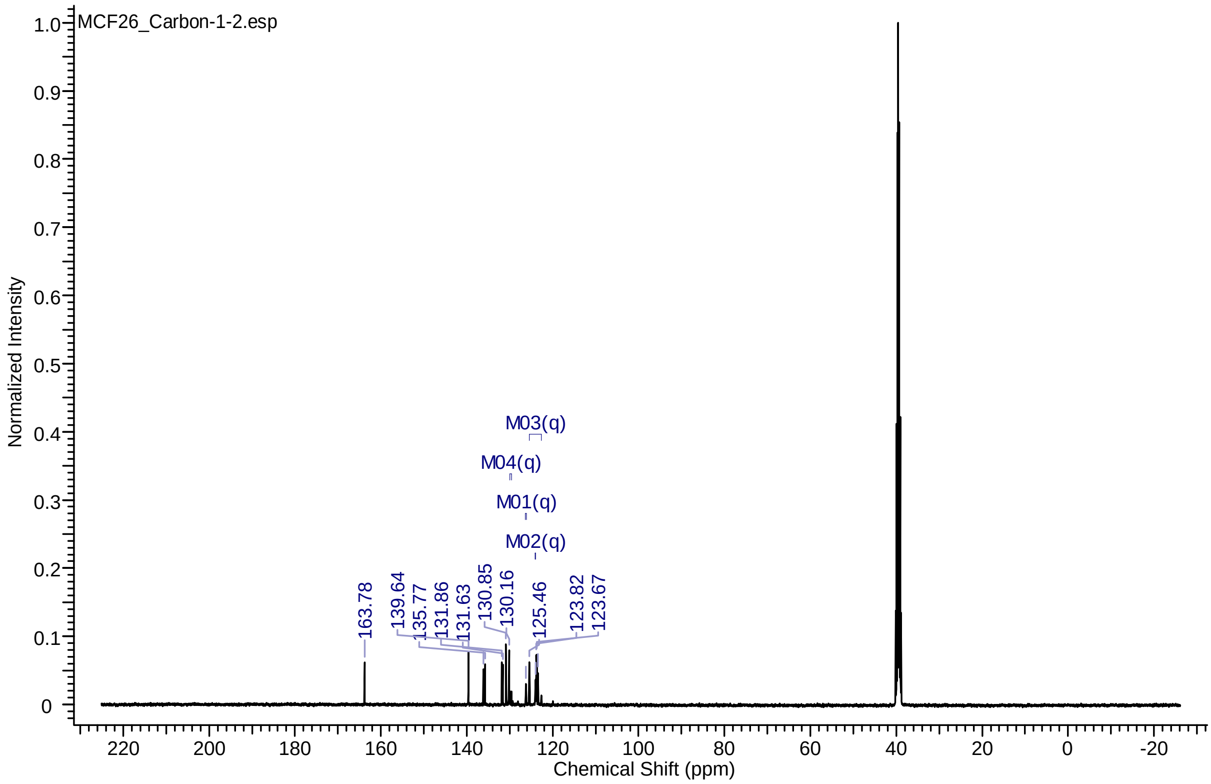


**Figure S18.** $^{13}$C-NMR (DMSO-d$_6$) spectrum of (2*E*)-*N*-(2,5-dichlorophenyl)-3-[3-(trifluoromethyl)phenyl]prop-2-enamide (**1n**).

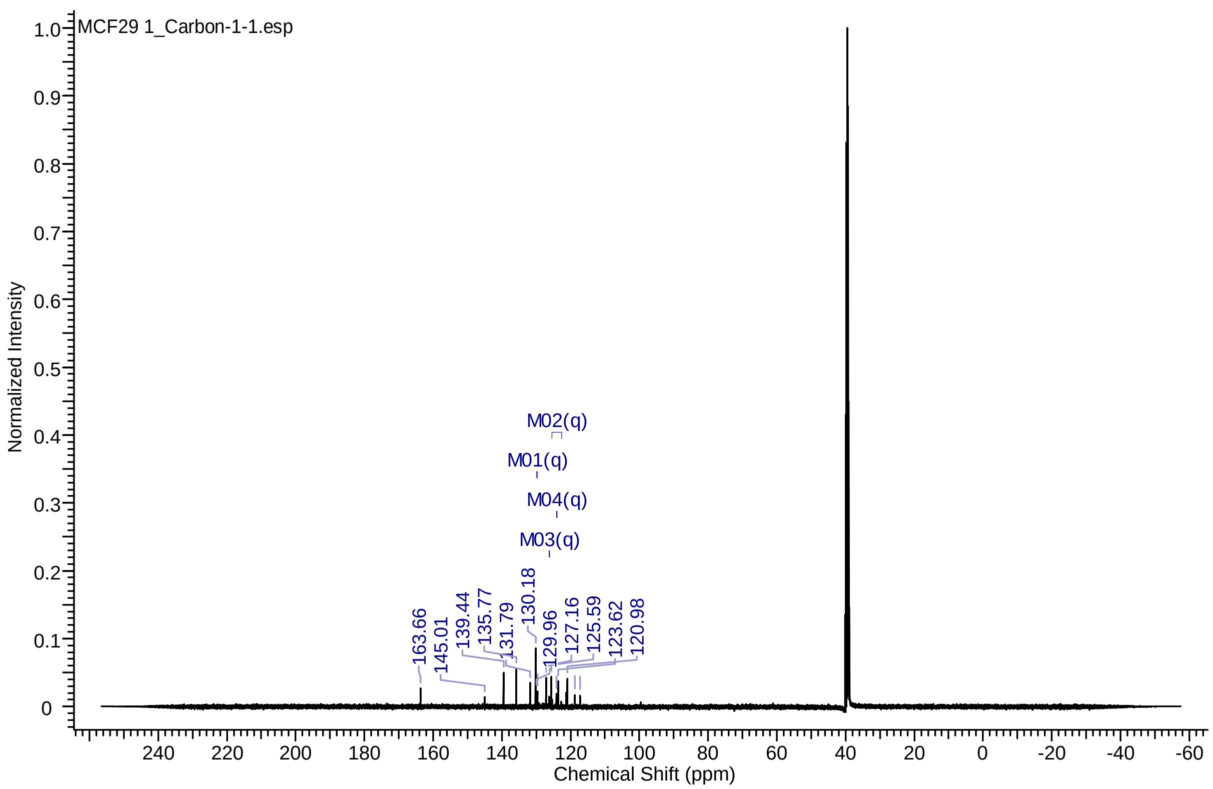


**Figure S19.** $^{13}$C-NMR (DMSO-$d_6$) spectrum of (2*E*)-*N*-(3,5-dichlorophenyl)-3-[3-(trifluoromethyl)phenyl]prop-2-enamide (**1o**).

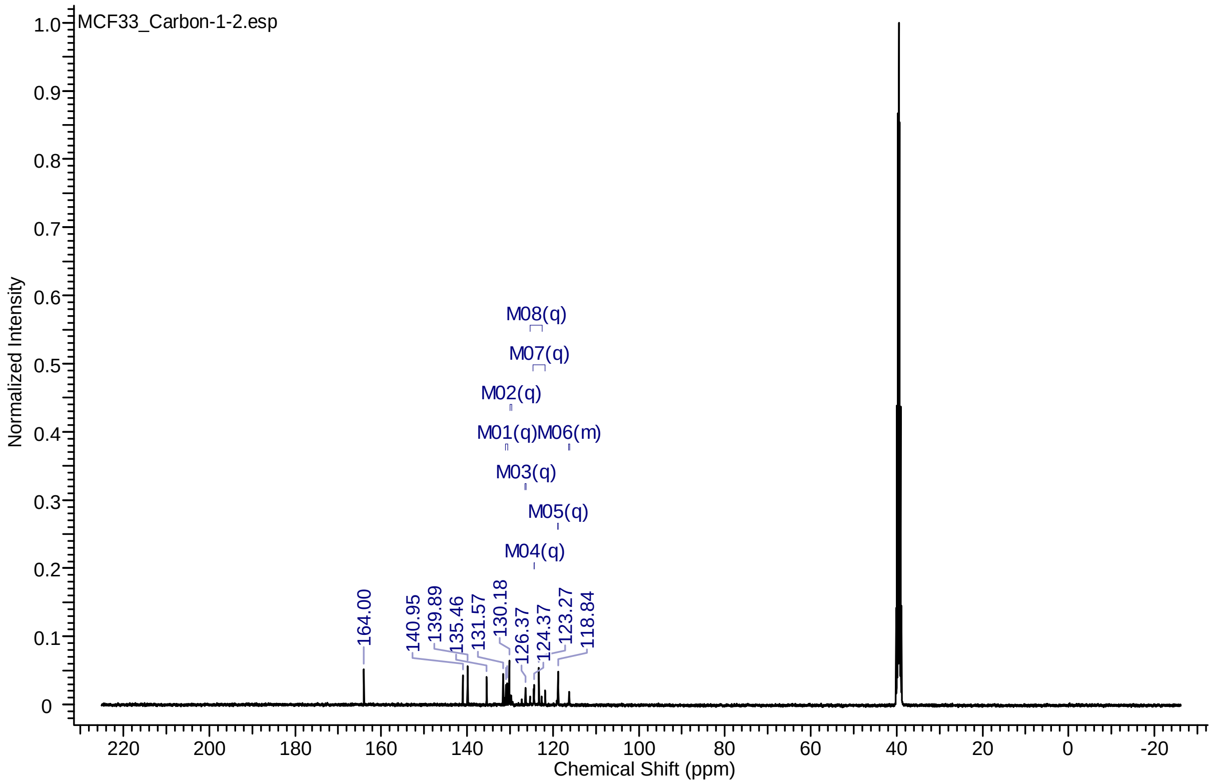


**Figure S20.** $^{13}$C-NMR (DMSO-$d_6$) spectrum of (2*E*)-*N*-[3,5-bis(trifluoromethyl)phenyl]-3-[3-(trifluoromethyl)phenyl]prop-2-enamide (**1p**).

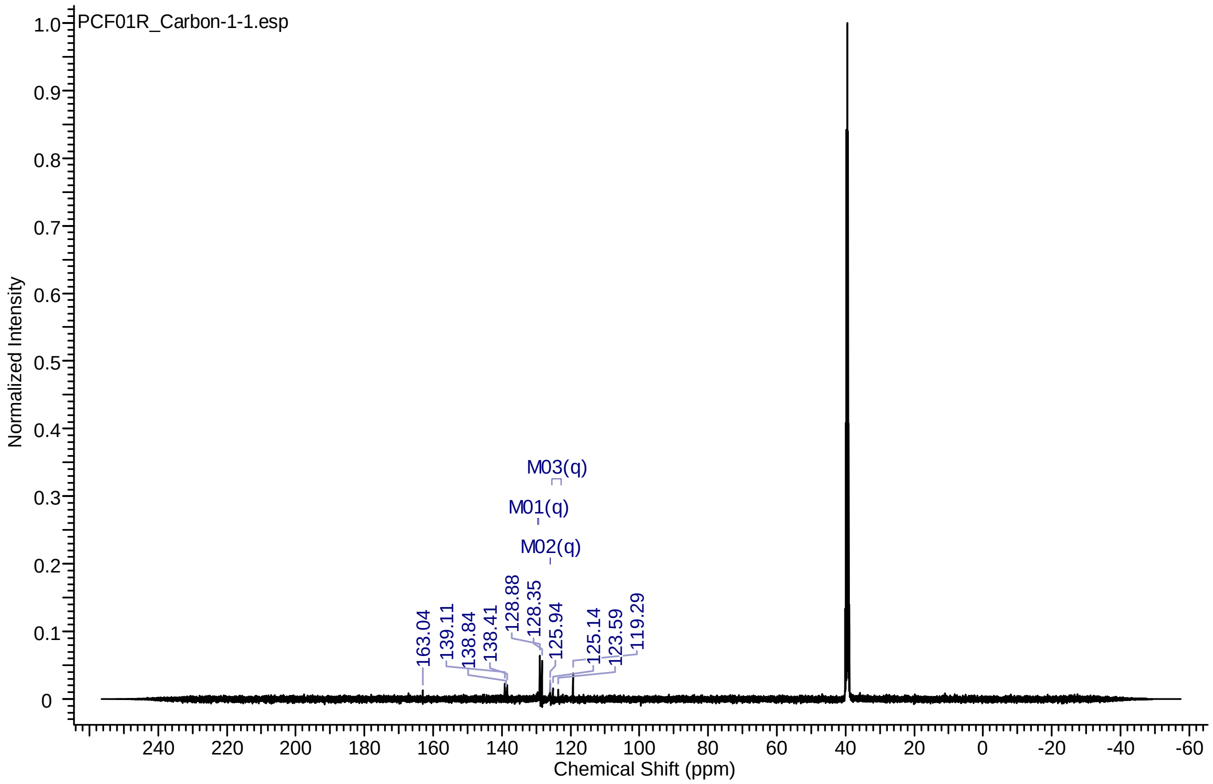


**Figure S21.** $^{13}$C-NMR (DMSO-$d_6$) spectrum of (2*E*)-*N*-phenyl-3-[3-(trifluoromethyl)phenyl]prop-2-enamide (**2a**).

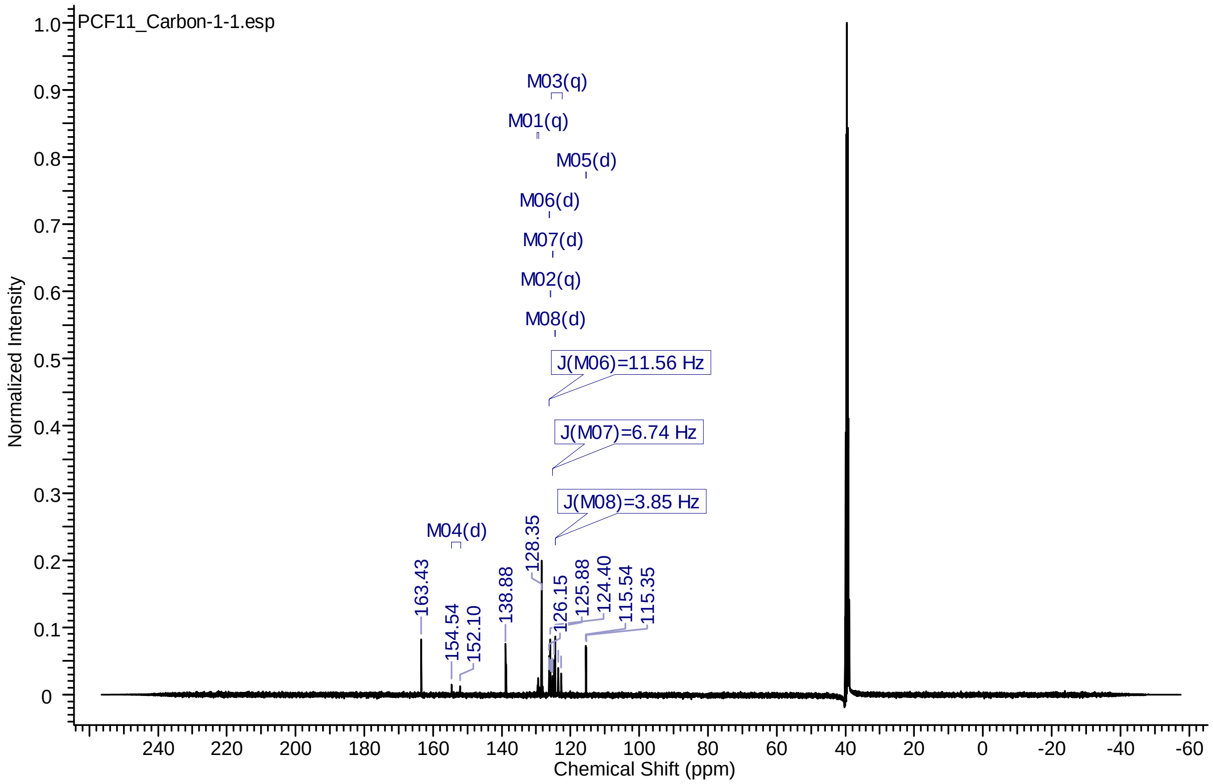


**Figure S22.** $^{13}$C-NMR (DMSO-$d_6$) spectrum of (2*E*)-*N*-(2-fluorophenyl)-3-[4-(trifluoromethyl)phenyl]prop-2-enamide (**2b**).

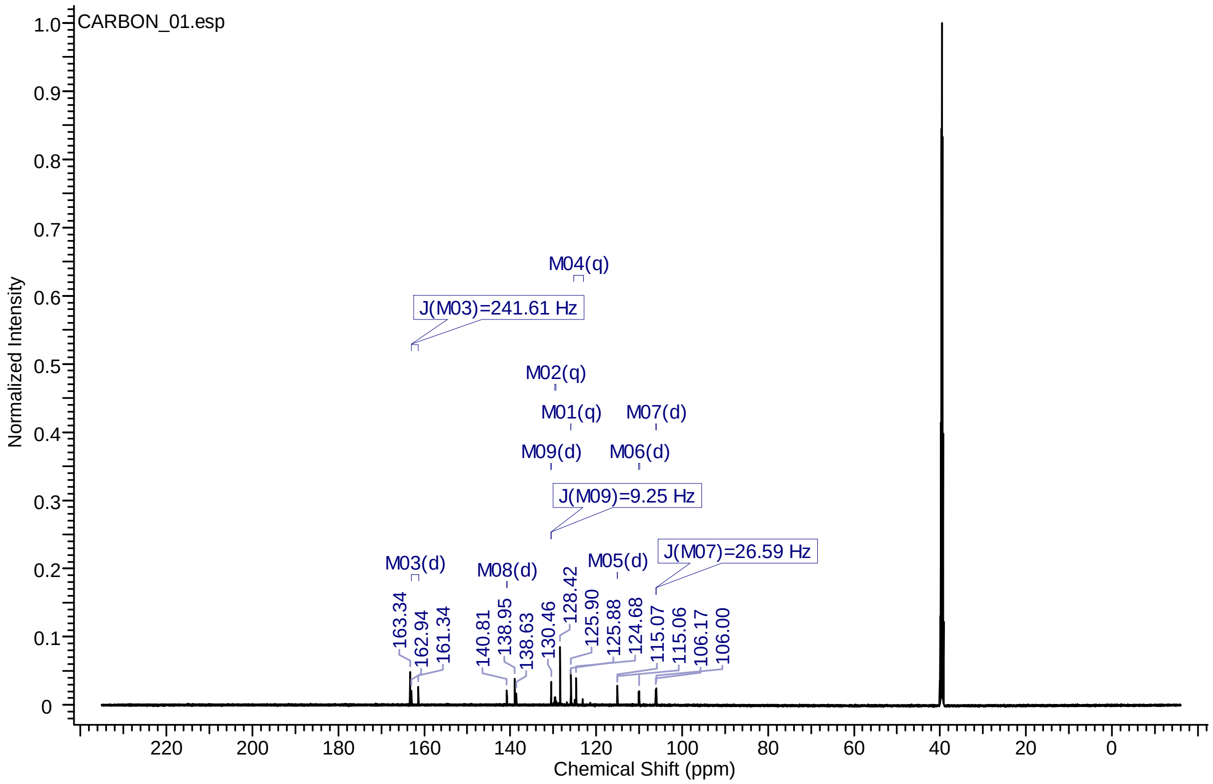


**Figure S23.** $^{13}$C-NMR (DMSO-d$_6$) spectrum of (2*E*)-*N*-(3-fluorophenyl)-3-[4-(trifluoromethyl)phenyl]prop-2-enamide (**2c**).

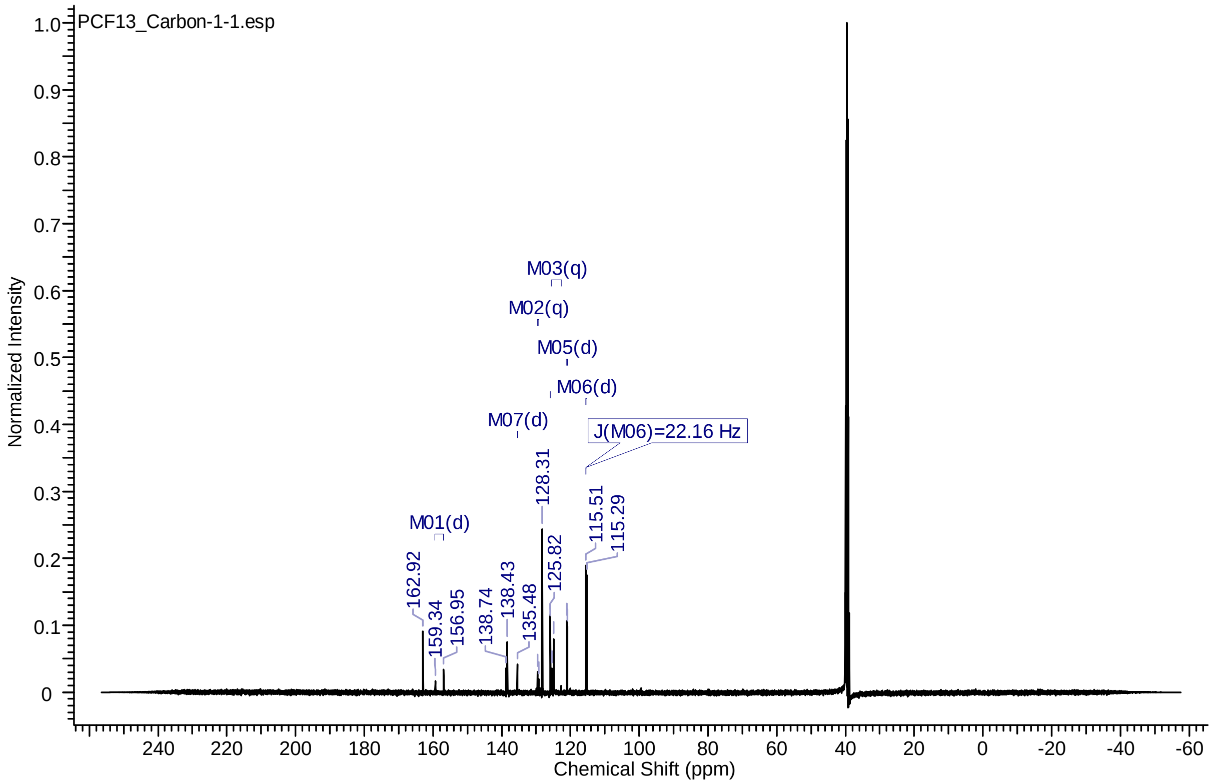


**Figure S24.** $^{13}$C-NMR (DMSO-d$_6$) spectrum of (2*E*)-*N*-(4-fluorophenyl)-3-[4-(trifluoromethyl)phenyl]prop-2-enamide (**2d**).

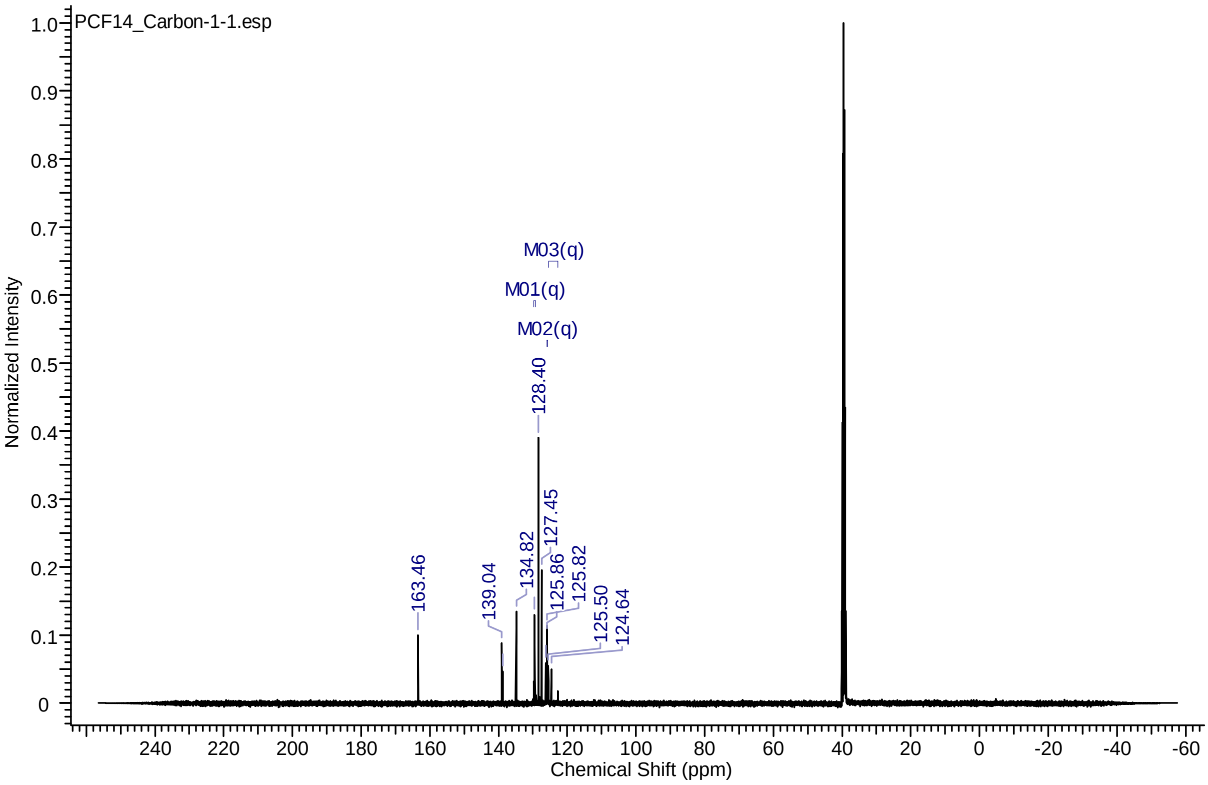


**Figure S25.** $^{13}$C-NMR (DMSO-d$_6$) spectrum of (2*E*)-*N*-(2-chlorophenyl)-3-[4-(trifluoromethyl)phenyl]prop-2-enamide (**2e**).

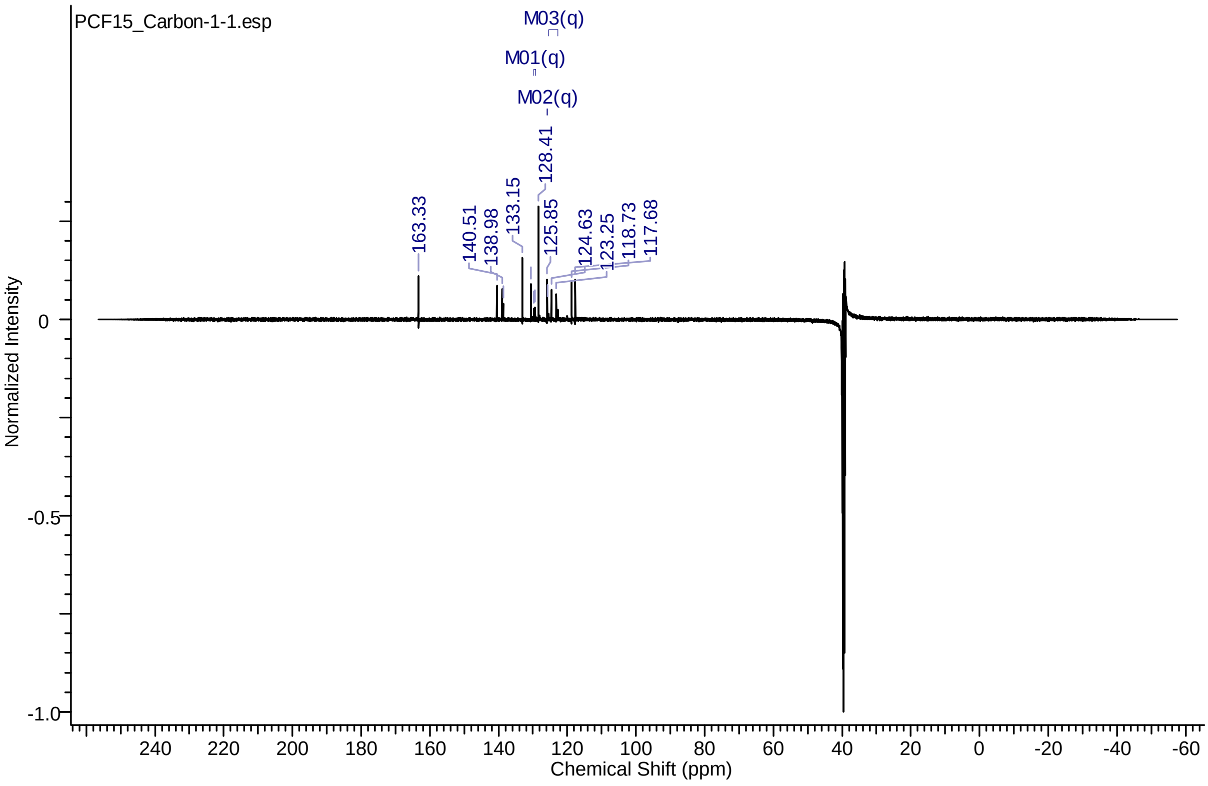


**Figure S26.** $^{13}$C-NMR (DMSO-d$_6$) spectrum of (2*E*)-*N*-(3-chlorophenyl)-3-[4-(trifluoromethyl)phenyl]prop-2-enamide (**2f**).

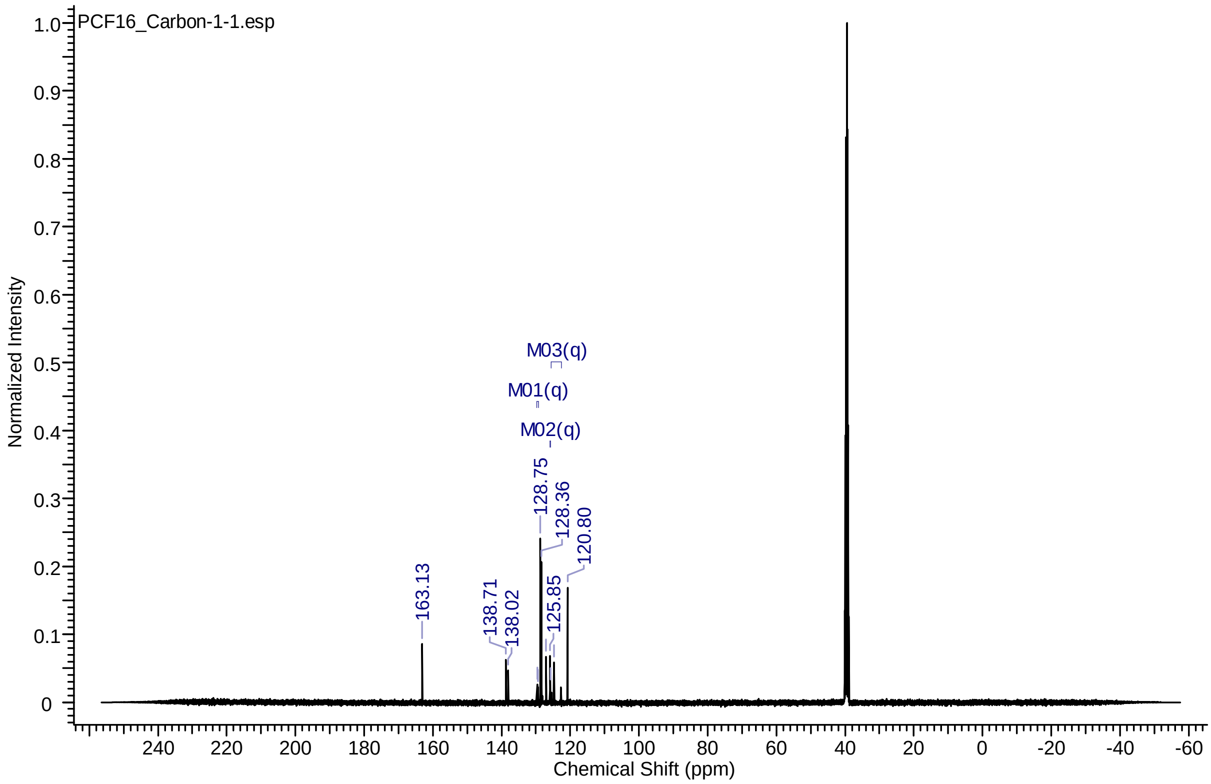


**Figure S27.** $^{13}$C-NMR (DMSO-$d_6$) spectrum of (2*E*)-*N*-(4-chlorophenyl)-3-[4-(trifluoromethyl)phenyl]prop-2-enamide (**2g**).

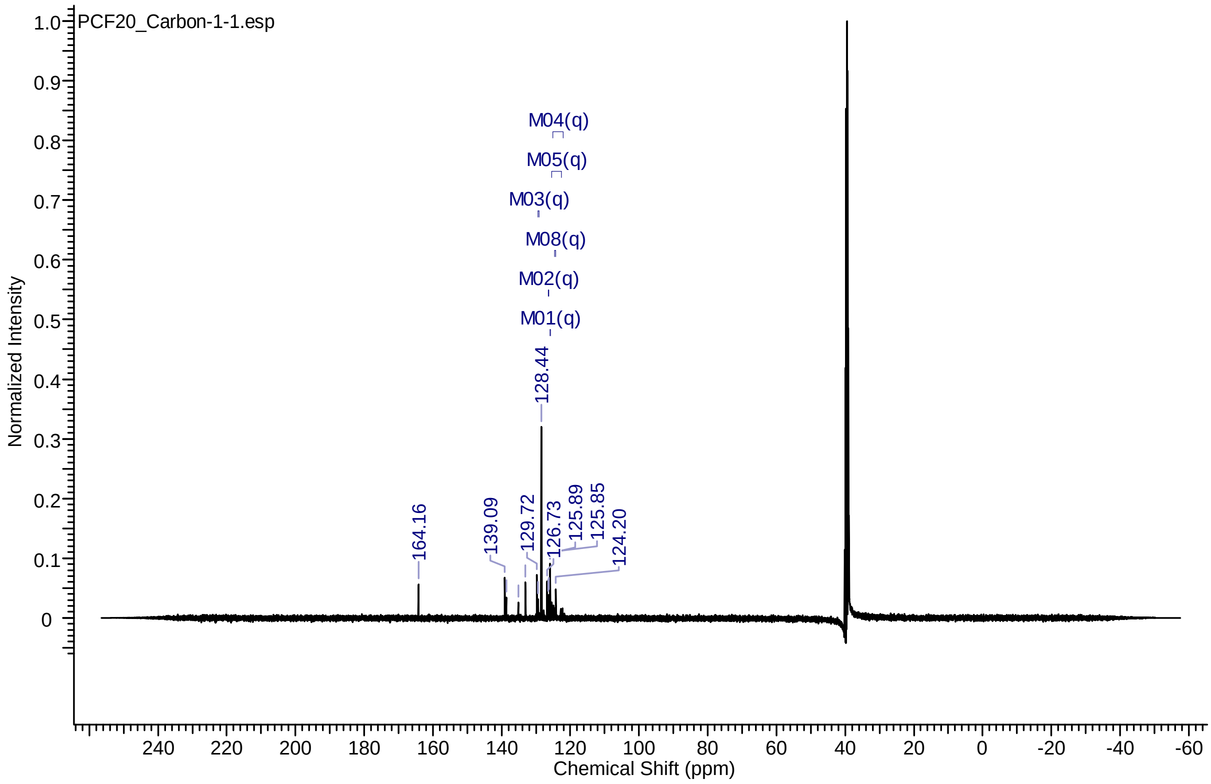


**Figure S28.** $^{13}$C-NMR (DMSO-$d_6$) spectrum of (2*E*)-*N*-[2-(trifluoromethyl)phenyl]-3-[4-(trifluoromethyl)phenyl]prop-2-enamide (**2h**).

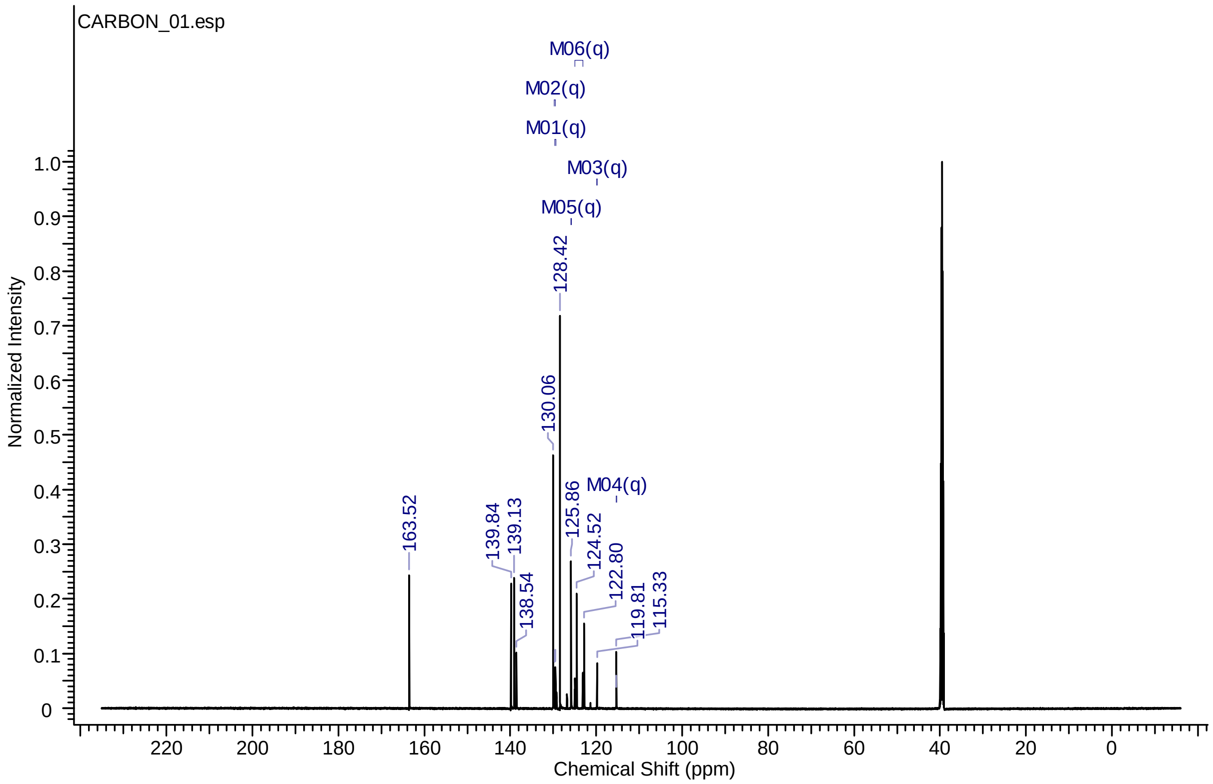


**Figure S29.** $^{13}$C-NMR (DMSO-$d_6$) spectrum of (2*E*)-*N*-[3-(trifluoromethyl)phenyl]-3-[4-(trifluoromethyl)phenyl]prop-2-enamide (**2i**).

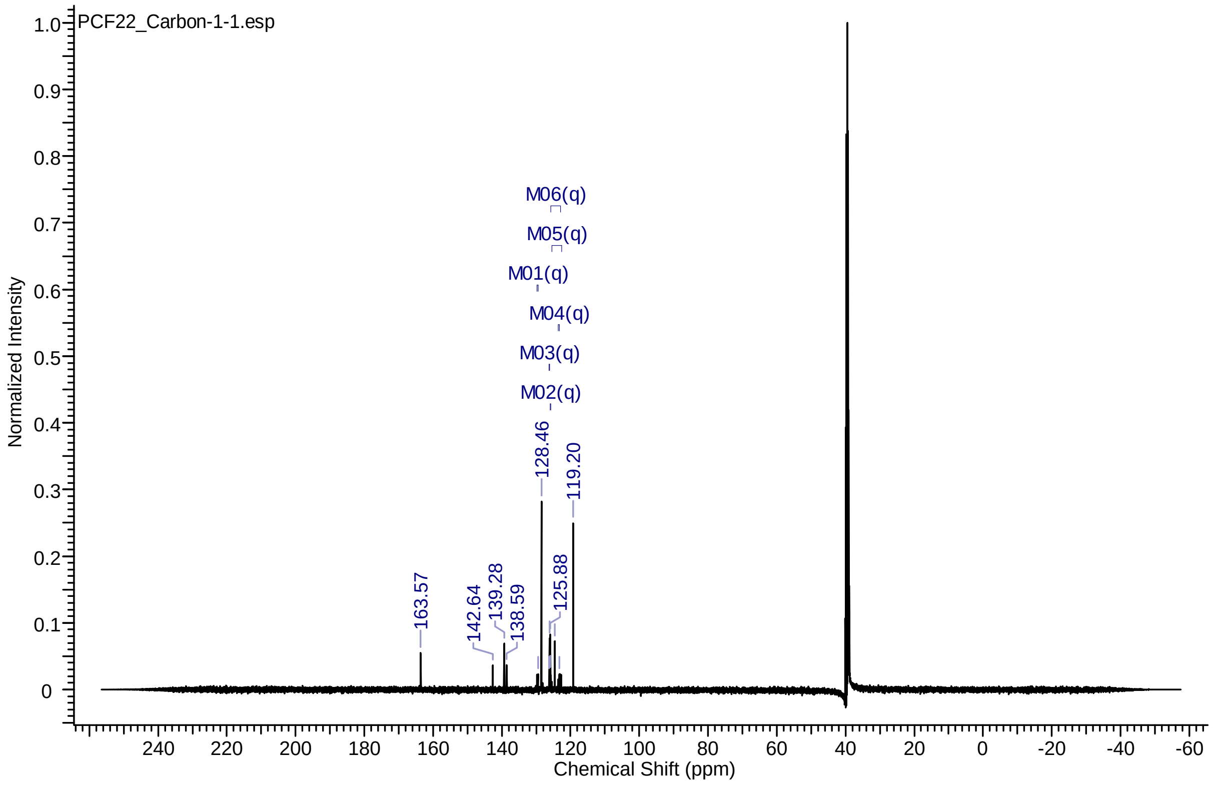


**Figure S30.** $^{13}$C-NMR (DMSO-$d_6$) spectrum of (2*E*)-*N*,3-bis[4-(trifluoromethyl)phenyl]prop-2-enamide (**2j**).

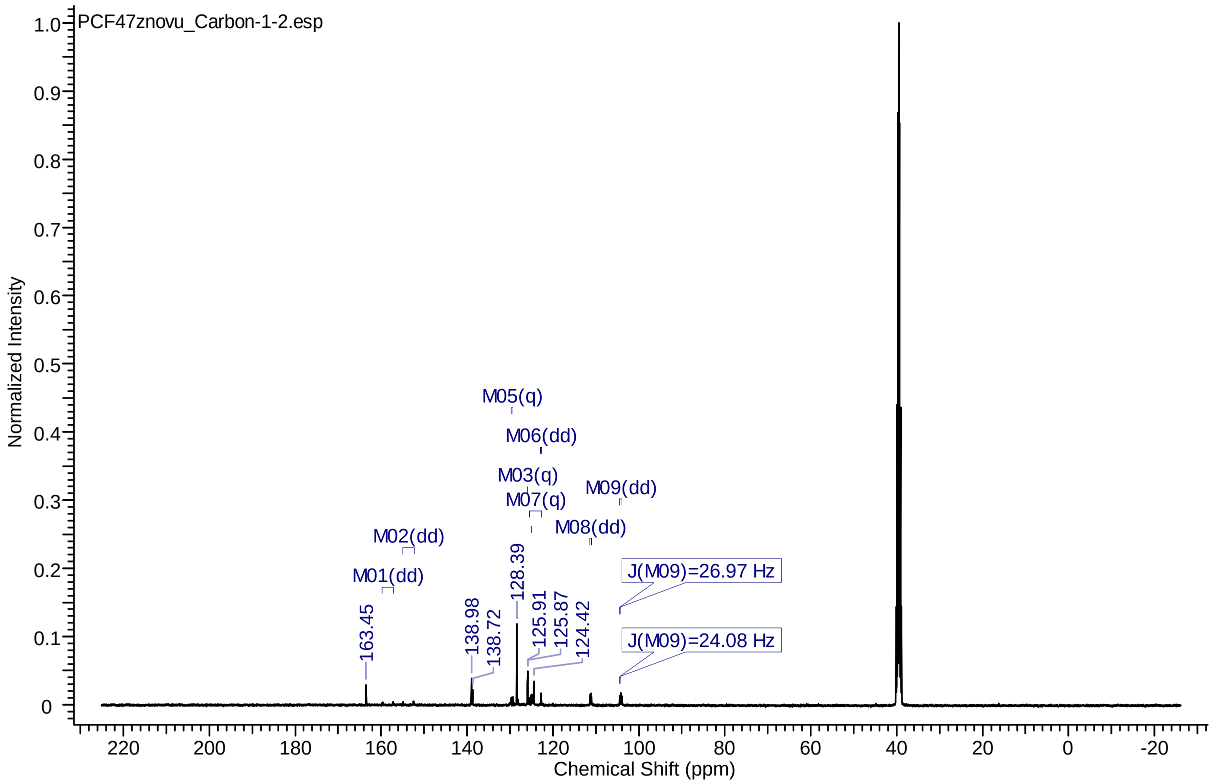


**Figure S31.** $^{13}$C-NMR (DMSO-d$_6$) spectrum of (2*E*)-*N*-(2,4-difluorophenyl)-3-[4-(trifluoromethyl)phenyl]prop-2-enamide (**2k**).

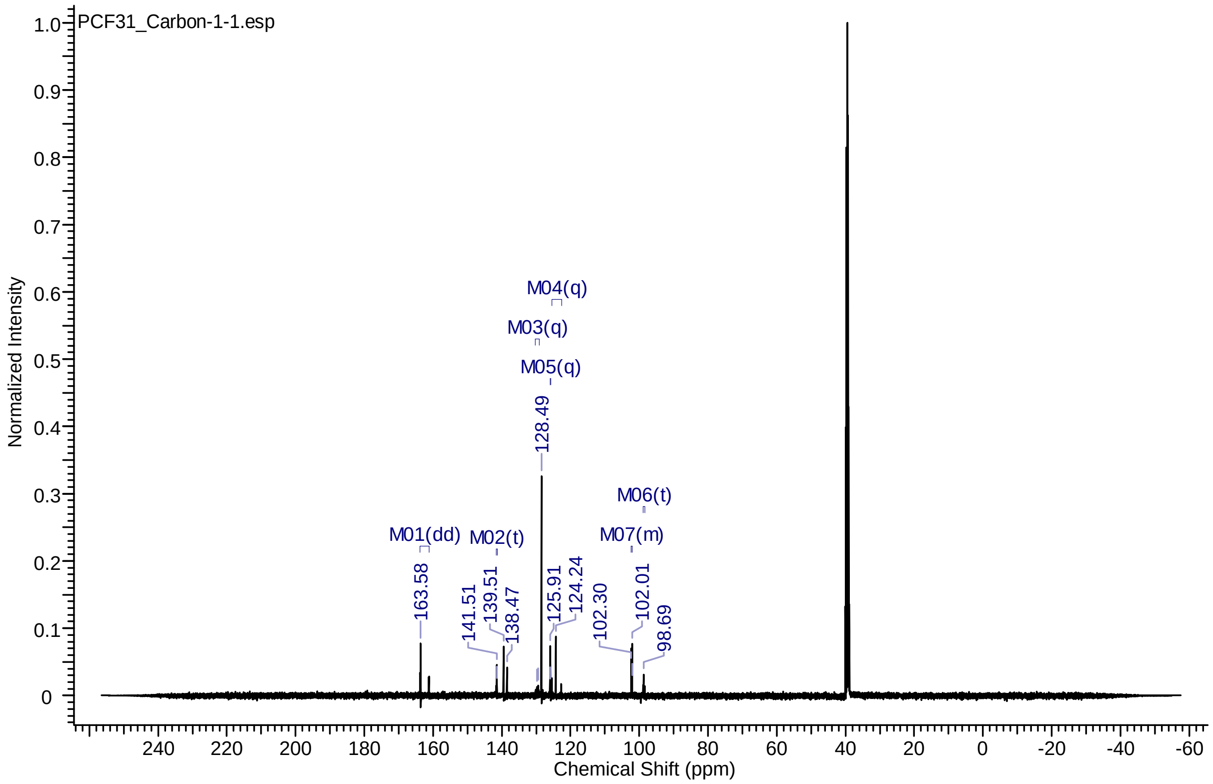


**Figure S32.** $^{13}$C-NMR (DMSO-d$_6$) spectrum of (2*E*)-*N*-(3,5-difluorophenyl)-3-[4-(trifluoromethyl)phenyl]prop-2-enamide (**2l**).

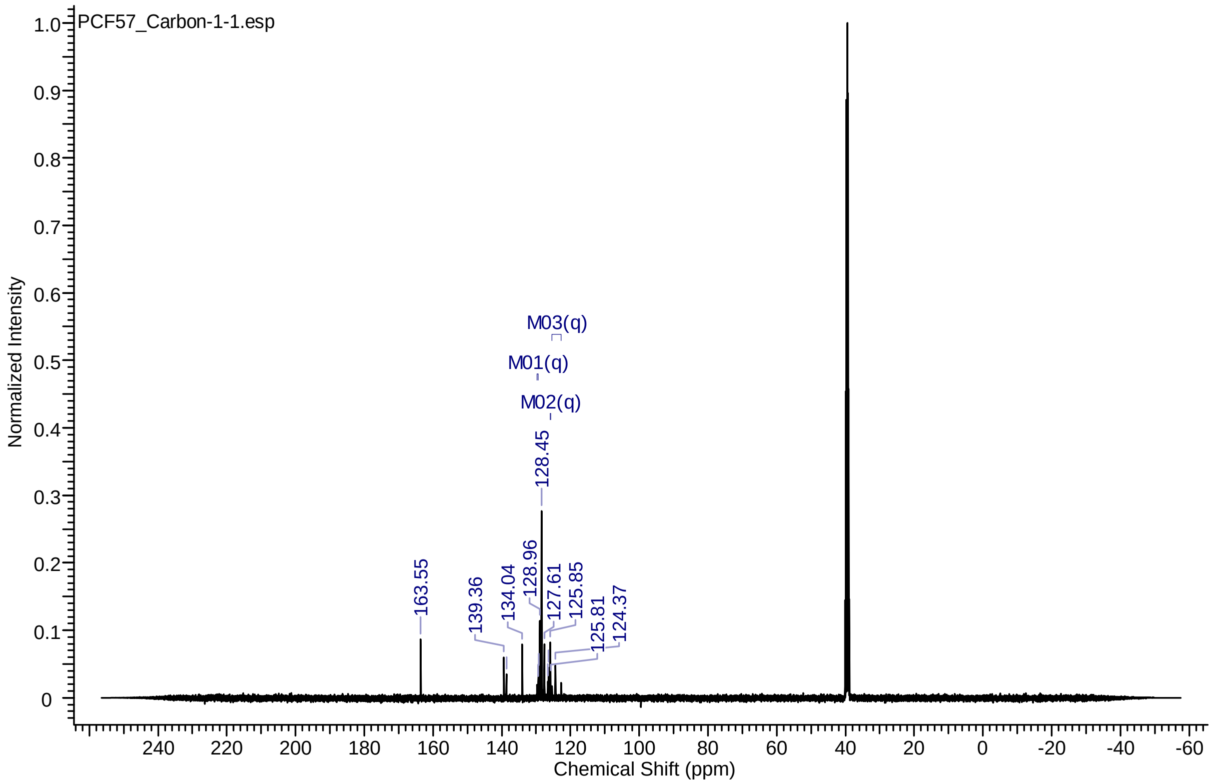


**Figure S33.** $^{13}$C-NMR (DMSO-d$_6$) spectrum of (2*E*)-*N*-(2,4-dichlorophenyl)-3-[4-(trifluoromethyl)phenyl]prop-2-enamide (**2m**).

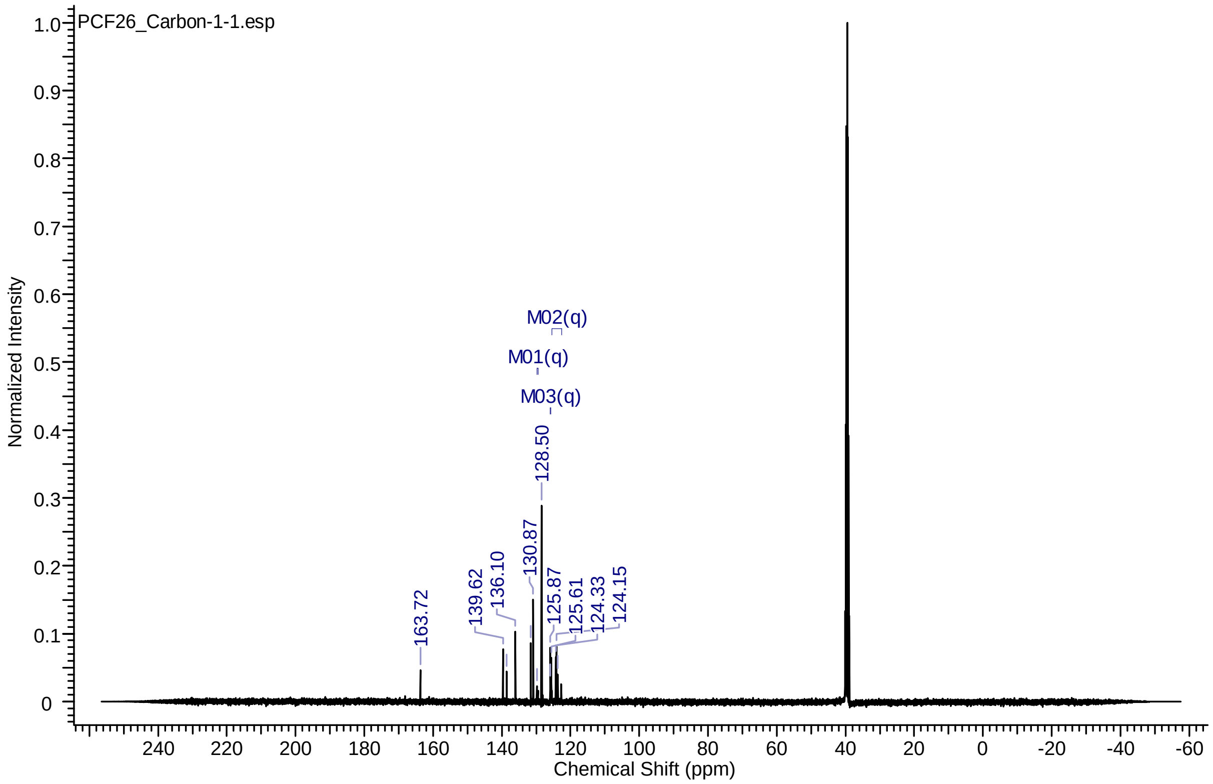


**Figure S34.** $^{13}$C-NMR (DMSO-d$_6$) spectrum of (2*E*)-*N*-(2,5-dichlorophenyl)-3-[4-(trifluoromethyl)phenyl]prop-2-enamide (**2n**).

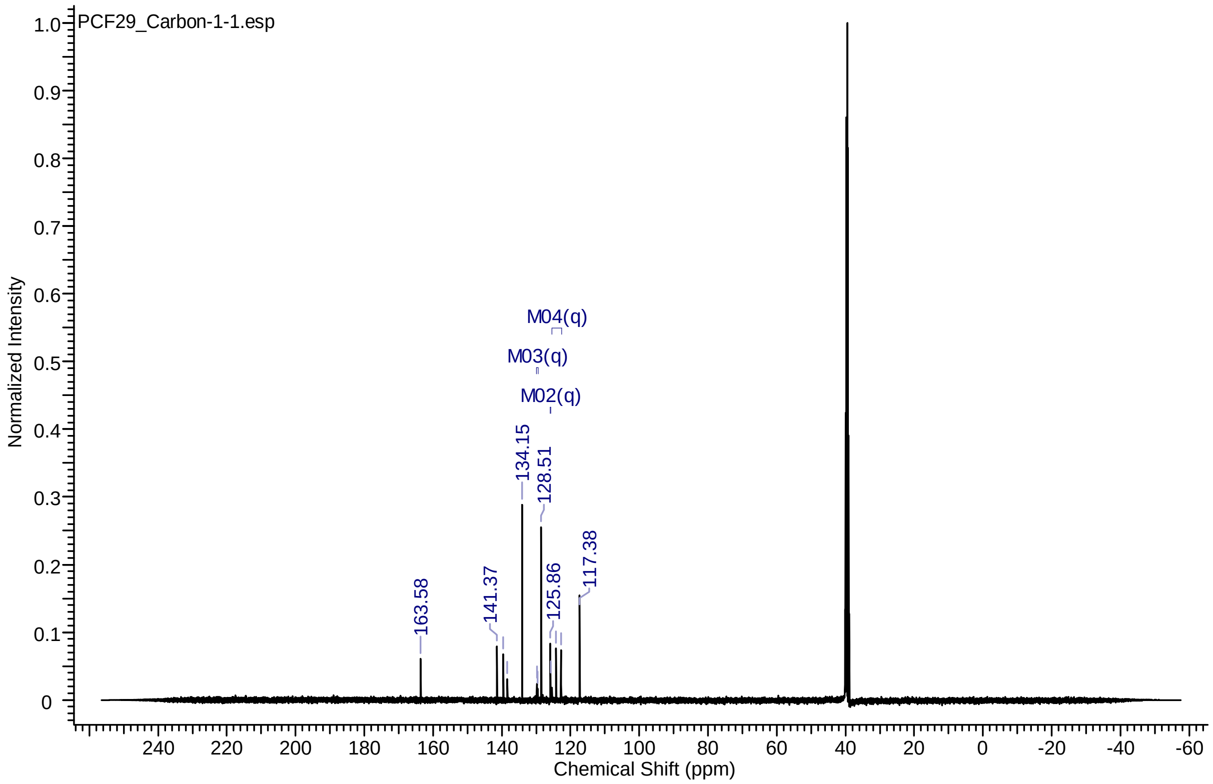


**Figure S35.** $^{13}$C-NMR (DMSO-d$_6$) spectrum of (2*E*)-*N*-(3,5-dichlorophenyl)-3-[4-(trifluoromethyl)phenyl]prop-2-enamide (**2o**).

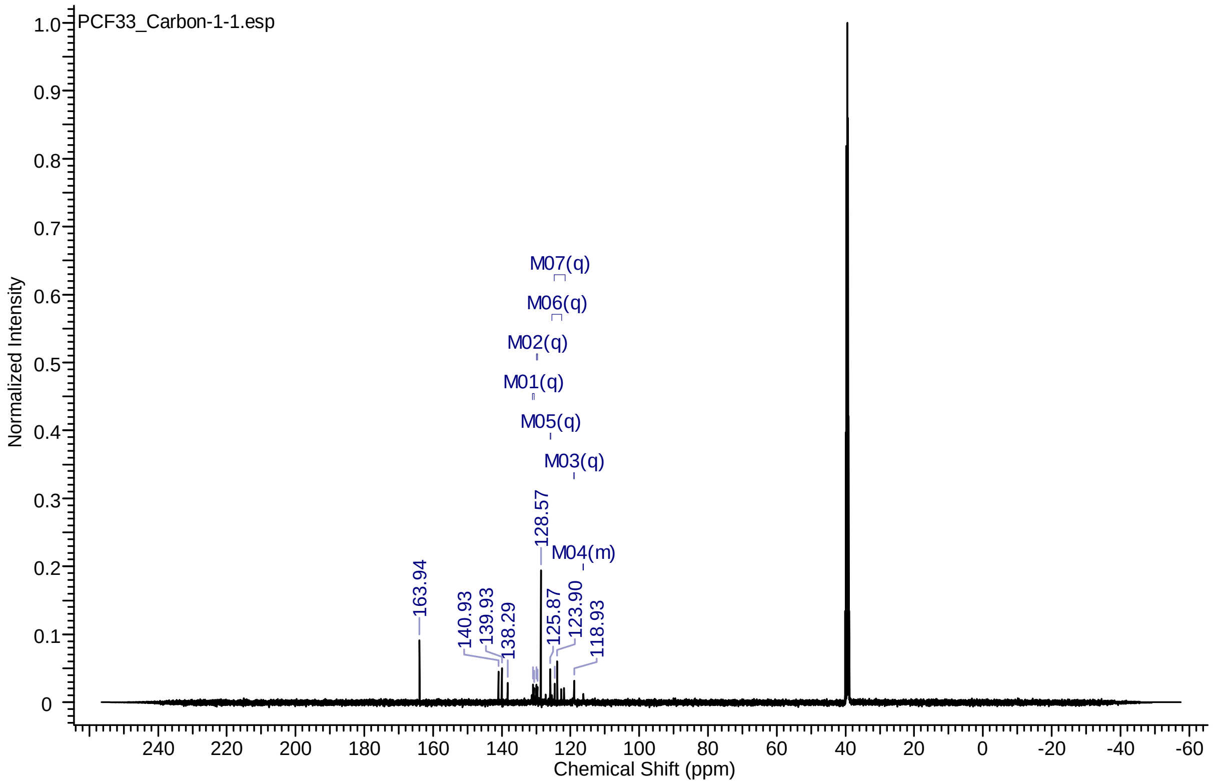


**Figure S36.** $^{13}$C-NMR (DMSO-d$_6$) spectrum of (2*E*)-*N*-[3,5-bis(trifluoromethyl)phenyl]-3-[4-(trifluoromethyl)phenyl]prop-2-enamide (**2p**).
